# Dealing with multi‐source and multi‐scale information in plant phenomics: the ontology‐driven Phenotyping Hybrid Information System

**DOI:** 10.1111/nph.15385

**Published:** 2018-08-28

**Authors:** Pascal Neveu, Anne Tireau, Nadine Hilgert, Vincent Nègre, Jonathan Mineau‐Cesari, Nicolas Brichet, Romain Chapuis, Isabelle Sanchez, Cyril Pommier, Brigitte Charnomordic, François Tardieu, Llorenç Cabrera‐Bosquet

**Affiliations:** ^1^ MISTEA, INRA, Montpellier SupAgro, Université de Montpellier Montpellier 34060 France; ^2^ LEPSE, INRA, Montpellier SupAgro, Université de Montpellier Montpellier 34060 France; ^3^ UE DIASCOPE, INRA, Montpellier SupAgro, Université de Montpellier Montpellier 34060 France; ^4^ INRA, UR1164 URGI – Research Unit in Genomics‐Info INRA de Versailles‐Grignon Route de Saint‐Cyr Versailles 78026 France

**Keywords:** data integration, data science, Information System, interoperability, knowledge, ontology, open source, phenomics

## Abstract

- Phenomic datasets need to be accessible to the scientific community. Their reanalysis requires tracing relevant information on thousands of plants, sensors and events.
- The open‐source Phenotyping Hybrid Information System (PHIS) is proposed for plant phenotyping experiments in various categories of installations (field, glasshouse). It unambiguously identifies all objects and traits in an experiment and establishes their relations via ontologies and semantics that apply to both field and controlled conditions. For instance, the genotype is declared for a plant or plot and is associated with all objects related to it. Events such as successive plant positions, anomalies and annotations are associated with objects so they can be easily retrieved.
- Its ontology‐driven architecture is a powerful tool for integrating and managing data from multiple experiments and platforms, for creating relationships between objects and enriching datasets with knowledge and metadata. It interoperates with external resources via web services, thereby allowing data integration into other systems; for example, modelling platforms or external databases.
- It has the potential for rapid diffusion because of its ability to integrate, manage and visualize multi‐source and multi‐scale data, but also because it is based on 10 yr of trial and error in our groups.

## Introduction

In recent years, plant phenomics has produced massive datasets involving millions of images in experiments performed in the field and in controlled conditions, concerning hundreds of genotypes at different phenological stages (Furbank & Tester, 2011; Fiorani & Schurr, 2013). These datasets also involve the outputs of hundreds of sensors for tens of variables characterizing plants, soil and air (Salehi *et al*., 2015; Negin & Moshelion, 2016; Rebetzke *et al*., 2016). They often also involve ‐omic data associated with imaged plants (Hannemann *et al*., 2009; Großkinsky *et al*., 2017). Plant phenomics is increasingly multi‐source and multi‐scale, with joint analyses of information originating from different phenotyping platforms and fields. Taken together, these datasets are unprecedented resources for identifying and testing novel mechanisms and models (Tardieu *et al*., 2017). They are extremely expensive, and also contain so much information that the group who has collected a dataset most often has not all the required skills, resources and scientific questions to perform every relevant analysis they may allow. Hence, there is an increasing need to make them available to a range of users, allowing reanalyses and combination with other datasets to generate new knowledge (Adam‐Blondon *et al*., 2016; Arend *et al*., 2016; Leonelli *et al*., 2017).

The reuse of data frequently presents the difficulty of insufficient information besides phenotypic data themselves (Hannemann *et al*., 2009; Gkoutos *et al*., 2017). Most measured traits differ between experiments for a given genotype because of environmental conditions (Massonnet *et al*., 2010; Malosetti *et al*., 2013). Hence, phenotypic datasets in each experiment need to be associated with detailed environmental information for meta‐analyses. Furthermore, each plant or sub‐plot senses different environmental conditions within a given field, glasshouse or growth chamber (Granier *et al*., 2006; Cabrera‐Bosquet *et al*., 2016), so keeping track of the position of each plant or plot is essential. This is widely accepted for field experiments in view of the large variability of traits and yield within and between fields, which can be accounted for by using mixed models (van Eeuwijk *et al*., 2010; Bustos‐Korts *et al*., 2016). Paradoxically, this is less accepted in controlled conditions in which the spatial distribution of environmental conditions and the *x*–*y* positions of plants are seldom stored in databases. Uncertainties about plant management (e.g. times of sampling, imaging, or irrigation times) and identification (genotype, seed lot) are also frequent obstacles to meta‐analyses. Finally, it increasingly appears that data analyses themselves are difficult to reproduce if each step, from sensor to trait, has not been traced (Pradal *et al*., 2017; Tardieu *et al*., 2017).

Reconciling phenotypic information in the field and in controlled conditions is a crucial challenge. Whereas it is accepted that measuring yield in controlled conditions is most often nonrelevant (Poorter *et al*., 2016), the high‐throughput measurement of physiological variables in the field is often impossible; for example, the precise measurement of water or nutrient fluxes through the plant, or of architectural features of root or shoot systems. Such measurements are possible in controlled conditions, opening the way to the dissection of the genetic architecture of physiological traits (Mairhofer *et al*., 2013; Cabrera‐Bosquet *et al*., 2016; Coupel‐Ledru *et al*., 2016; Kalogiros *et al*., 2016; Alvarez Prado *et al*., 2018). Combining data in field and controlled conditions is possible, and provides valuable information for analysing and predicting the genotype × environment interaction of both traits and yields (Reymond *et al*., 2003; Lacube *et al*., 2017; Tardieu *et al*., 2018). An essential feature of phenomic information systems is therefore to facilitate these trans‐scale joint analyses of experiments in field and controlled conditions.

A major challenge in plant phenomics is therefore to design information systems able to organize and store heterogeneous datasets including thousands of objects as different as, for example, images, spectra, time courses of variables, parameters of image analysis, *x*–*y* positions of plants/plots, biomass, or yield. Finding and accessing data originating from multiple sources (including contextual information associated with individual plants, plots or sensors) and taking into account spatial and temporal relationships between objects (i.e. plants, organs, sensors and phenotyping facilities) is central for both real‐time monitoring of experiments and for post‐experiment interpretation of measured traits (Cabrera‐Bosquet *et al*., 2012). The challenge is still larger if information systems aim to organize data originating from different groups, different scales and different infrastructures with FAIR (findable, accessible, interoperable and reusable) requirements (Wilkinson *et al*., 2016) for tracing data, but also protocols, methods and workflows, in such a way that scientists who did not perform experiments can reuse data. Recently, several papers have recommended standardization protocols and enrichment of datasets with metadata (Arend *et al*., 2014, 2016; Junker *et al*., 2014; Krajewski *et al*., 2015; Ćwiek‐Kupczyńska *et al*., 2016) and scientific workflows (Pradal *et al*., 2017). However, this information is rarely incorporated into information systems.

The use of open and extensible database schemas based on Ontology Web Language (OWL) (Grau *et al*., 2008) allows formalized description and contextual information of objects involved in experiments (Li *et al*., 2013; Krajewski *et al*., 2015; Ćwiek‐Kupczyńska *et al*., 2016; Le Ngoc *et al*., 2016). Tools using ontologies and semantics are available in functional genomics and systems biology (Jones *et al*., 2007; Gkoutos *et al*., 2017). Ontology‐centred architectures such as Xeml Lab (Hannemann *et al*., 2009), Podd (Li *et al*., 2013) and Silex (Information System for Experiment, https://www6.montpellier.inra.fr/mistea_eng/Projects/Silex) have been proposed for plant phenomic studies. However, most published or commercial databases for plant phenomics are still specifically designed to handle and store data from particular installations or species (Fabre *et al*., 2011; Nagel *et al*., 2012; Klukas *et al*., 2014; Coppens *et al*., 2017). Hence, the wide variety of phenotypic, environmental and contextual data is spread in a range of databases, lab books and individual text/spreadsheet files, thereby complicating the traceability and access to experimental results and associated metadata.

Here, we present a suite of methods, synthesized in the open‐source Phenotyping Hybrid Information System (PHIS) for integrating and sharing multi‐source and multi‐scale data (in particular those obtained in both controlled and field conditions), and semantic annotation of experiments with knowledge and metadata. This system is available to the public community and has been deployed in installations in both field and controlled conditions. Its main interest is that most of its properties have been built based on trial and error over 10 yr of phenotyping practices in groups specialized in either information technology or in phenotyping. For better legibility, we restrict examples in this paper to two installations located in Montpellier.

## Materials and Methods

### PHIS architecture

**Figure 1:**
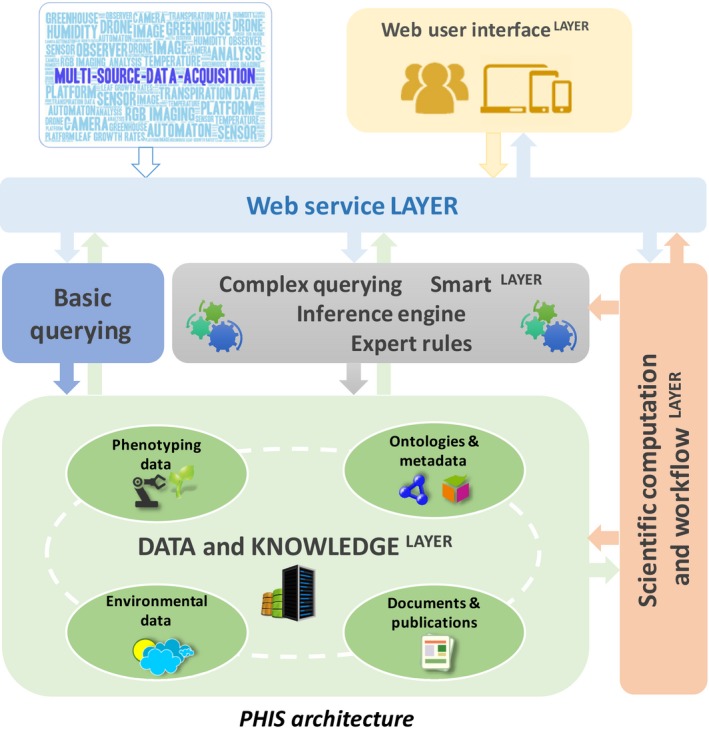
The Phenotyping Hybrid Information System (PHIS) architecture consisting of five major components structured in different layers, which include (1) a web user interface, (2) a data and knowledge storage layer, (3) a web service layer, (4) a smart layer and (5) a scientific computation and workflow layer.

PHIS is a hybrid information system derived from Silex, a collaborative project aiming to provide software components for experimental data and knowledge management between different research groups. The PHIS architecture consists of five components structured in layers; namely, a web user interface, a data and knowledge layer, a web service layer, a smart layer and a scientific computation and workflow layer (Fig. 1).

### Web user interface

PHIS is accessed through an interactive web user interface (http://www.phis.inra.fr) after user login. A user belongs to one or several groups and has access to data with different access rights (admin, owner or guest). Administration level gives full access to all content, owners have access to their own datasets and to public data, and guests have access to public data only. The web user interface contains three main menus, which are dynamically adapted depending whether the experiment is performed in the field or in controlled conditions. The Experimental Organisation menu contains information about the experimental resources, including projects, experiments, infrastructure, plants, germplasm, devices, events, phenotypic and environmental variables and object tracking tools (see Supporting Information Notes [Link], [Link], [Link] for technical details). The Data menu contains images, graphic visualization of phenotypic and environmental traits, as well as access to data analysis and workflows. A search engine allows advanced querying for entities like projects, experiments, domain specific objects (plants, sensors, etc.), images and genotypes using uniform resource identifiers (URIs) and/or filtering options (Notes S4). The Tools menu contains installation‐specific widgets, namely, a URI generator, a label quick response code (QR‐code) generator, access to the closed‐circuit television of facilities, a vocabulary and access to the web service application programming interface (API; Notes S5, S6). The Administration menu contains tools for managing users, groups, experimental facilities and control of data settings (variables, units and methods). The web user interface is implemented in PHP and HTML5, CSS3 and JavaScript (jQuery library, http://jquery.com). The skeleton of the application is developed with the Yii2 framework (http://yiiframework.com).

### Data and knowledge layer

PHIS contains phenotypic, experimental and environmental data. Tests were performed on field and controlled‐condition experiments. Field experiments were hosted at DIAPHEN (https://www.phenome-fppn.fr/phenome_eng/Facilities/Montpellier-Field) at INRA Mauguio (southeast of France, 43°36ʹ N, 03°58ʹE) over 20 ha, which provides access to high‐throughput phenotyping tools including soil and aerial vectors (carrying RGB, multi‐ and hyperspectral cameras and spectroradiometers) as well as a series of sensors for characterizing environmental conditions. Controlled‐conditions experiments were hosted at Montpellier Plant Phenotyping Platforms, M3P (https://www6.montpellier.inra.fr/lepse/M3P), which implements a series of tools with up to 500 sensors in parallel, three‐dimensional imaging cabins and automatisms (Sadok *et al*., 2005; Granier *et al*., 2006; Cabrera‐Bosquet *et al*., 2016).

Phenotypic data for field and platform experiments include online (i.e. automatically recorded) images, growth and transpiration kinetics and manually recorded phenotypic measurements. Experimental data include protocols, description of variables and plant material. Environmental data include sensor outputs (air temperature and humidity, light, soil tensiometers) or variables inferred using algorithms at high temporal and spatial resolution (e.g. local light and temperature). Currently, these installations have generated 20 million images and 250 million phenotypic measurements performed in > 4000 genotypes and 25 species, and 154 million environmental measurements, involving 86 terabytes.

Structured data (e.g. environmental data and standardized phenotypic variables) are stored using PostgreSQL and MongoDB. ‘Weakly’ structured data (e.g. plant observations and image analyses data) are stored using NoSQL technology (MongoDB). Raw images, thumbnails and analysed images (e.g. segmented images) are stored on a distributed storage system (iRODS) (https://irods.org/) (Rajasekar *et al*., 2010) with a replication mechanism and built‐in scripts that permanently check the consistency with the associated metadata. Metadata and semantic annotations are stored taking into account both the ontologies, implemented in OWL (https://www.w3.org/OWL/), and the knowledge resulting from experimental observations, which is formalized as instances represented using the Resource Description Framework (RDF) format, and stored in a RDF4J Triple Store (http://rdf4j.org). Triple data is a data entity (i.e. subject predicate object) like ‘plant736 participatesIn ExperimentA’ and a Triple Store is a database system dedicated to the storage and the retrieval of triples through semantic queries (SPARQL). The Triple Store also allows ontology‐based inferences, and provides web services accessible using SPARQL queries. The application ontologies are stored in an open‐access repository, AgroPortal (http://agroportal.lirmm.fr/; Jonquet *et al*., 2018); see Notes S7 for technical details. Data access is achieved by the web service layer or by performing CSV extraction.

### Web service layer

A web service layer enables interoperability and data exchange with other applications and systems. This service facilitates the maintenance of the information system and provides a simplified interface to the smart layer and to the data and knowledge layer. The web service is based on RESTFul (representational state transfer) developed using Swagger framework (https://swagger.io/), and all services are available by using URIs. It is developed in Java with Jersey implementation of JAX‐RS (Java API for RESTful Web Services) standard. It implements installations’ relevant services of the Breeding API (http://www.brapi.org/), which specifies a standard interface for plant phenotype databases to serve data to crop breeding applications. Web service outputs use the data‐interchange format JSON (JavaScript Object Notation; see Notes S6 for technical details).

### Smart layer, scientific computation and workflow layer

The Smart layer allows data to be interpretable for other communities by referring to external resources such as standardized semantic resources and reference or species‐specific ontologies as described in the Planteome project (Cooper *et al*., 2018). References are managed using the Simple Knowledge Organization System (Miles & Bechhofer, 2009) that allows support of standardized and advanced queries by using ontologies. These queries and the necessary inferences (subsumtion, transitivity, functional, etc.) are obtained by using an RDF4J engine included in the Triple Store. The scientific computation and workflow layer provides advanced visualization and statistical computing, including the automatic report generation, based in R (R Core Team, 2015), and enables computational analysis and workflows through the scientific platform Galaxy (https://galaxyproject.org/). JavaScript libraries are also used for user friendly and interactive data exploration, including detection of inconsistencies and manual semantic annotation. Export tools are available in different formats for graphic (PDF, JPG, PNG, SVG) and numeric data (HTML, csv, txt, xlsx, PDF and JSON). PHIS can integrate scientific workflows such as infraphenogrid (Pradal *et al*., 2017), which provides provenance functionalities. Technical details are provided as Notes S4.

### Application example

PHIS features are illustrated here by using phenotypic data obtained in two experiments involving 59 common maize (*Zea mays* L.) hybrids performed in both the field (DIAPHEN) and glasshouse (PHENOARCH) installations. Data are available at http://www.phis.inra.fr/under, an Open Source license (CC‐BY‐NC‐SA). The field experiment contains *c*. 10 000 scientific objects and 178 sensors, 10 000 images, 70 000 phenotypic observations, 20 000 annotations and 0.5 million environmental measurements. The glasshouse experiment contains 2204 scientific objects, 242 sensors, *c*. 2 million images, 10 million phenotypic observations, 15 000 annotations and > 4 million environmental measurements. Detailed information of the experiments, installations and measurement of environmental conditions are described in Cabrera‐Bosquet *et al*. (2016) and Brichet *et al*. (2017).

### Availability and requirements

The source code and user and developer documentation of the latest version of PHIS are available at https://github.com/OpenSILEX under a GNU Affero General Public License version 2. PHIS requires Java JRE or JDK v.1.7, PHP 5.6, PostgreSQL 10.1, RDF4j 2.2.1, MongoDB 3.4.4 and R 3.3.1 and runs on Linux, Mac and Microsoft Windows platforms.

## Results and Discussion

### A common information system for field and controlled‐condition experiments

**Figure 2:**
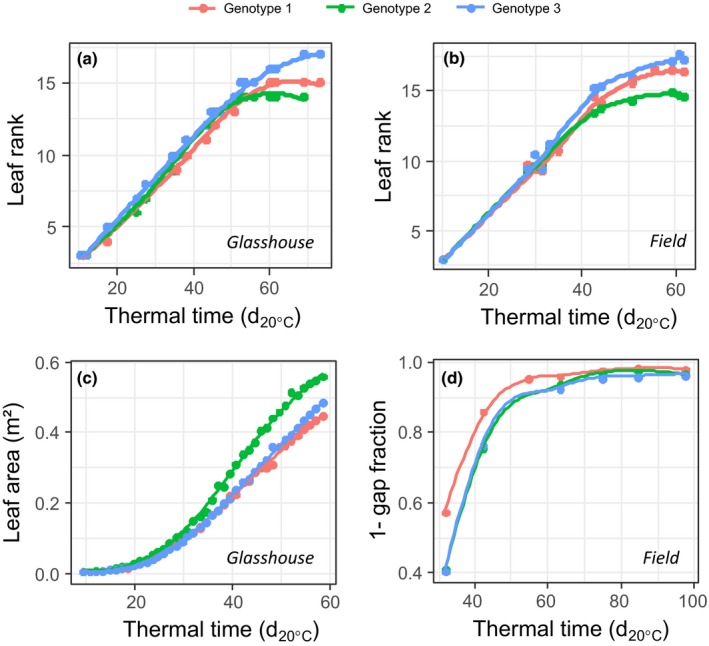
Example of trait variables that either (a, b) easily map between experiments in glasshouse and field or (c, d) do not map because of methodological difficulties. (a, b) Leaf appearance rate as function of thermal time for three genotypes in (a) glasshouse and (b) field experiments. (c, d) The progression of leaf area, either measured directly in the glasshouse (c) or via gap fraction (see Fig. 3b) in the field for the same three genotypes. For each genotype, points are the mean of nine and three replicates for the glasshouse and field experiments respectively.

**Figure 3:**
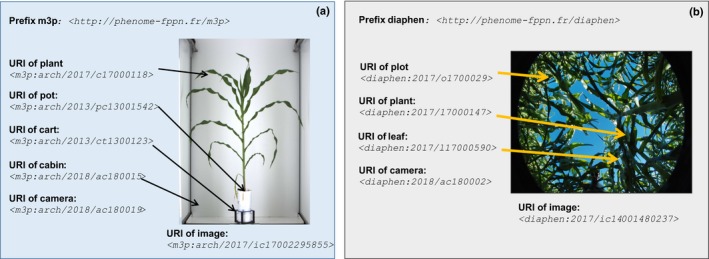
An example on the use of unique resource identifiers (URIs) for identifying all the objects present in single images taken in (a) glasshouse and (b) field experiment. (b) Exemplification of the concept of gap fraction in Fig. 2 and text; namely, the proportion of sky that is viewed in this picture. In the glasshouse, an image <*m3p:arch/2017/ic17002295855*> of a given plant <*m3p:arch/2017/c17000118*> that is placed in a pot <*m3p:arch/2013/pc13001542*> and a cart <*m3p:arch/2013/ct1300123*> is acquired in a cabin <*m3p:arch/2018/ac180015*> with an RGB camera <*m3p:arch/2018/ac180019*>. Note that Localinfra_a and installation1 in the text are represented by M3P and arch here, to match with supplementary information. In the field, an image <*diaphen:2017/ic14001480237*> of a plot <*diaphen:2017/o1700029*> containing a plant *diaphen:2017/17000147*> and a leaf <*diaphen:2017/l17000590*> is acquired using a hemispherical camera <*diaphen:2018/ac180002*>. The prefixes *m3p:* and *diaphen:* preceding URIs stand for < http://www.phenome-fppn.fr/m3p> and <http://www.phenome-fppn.fr/diaphen> respectively.

Combining field and controlled conditions in a common information system requires first ensuring that environmental conditions are measured in a compatible way, with common units and protocols, and second designing common ontologies of traits for both types of datasets. The first condition only requires the attention of experimenters, without large theoretical difficulty (Reymond *et al*., 2003; Lacube *et al*., 2017). The second condition is straightforward for traits that have a common definition at different scales, such as leaf appearance rate or leaf number per plant (Fig. 1a,b). However, other traits have different meanings and measurement procedures in field and controlled conditions, such as ‘plant height’, often defined as the highest green pixel corresponding to a plant in controlled conditions, vs the mean canopy height in the field. In the same way, ‘leaf area’ is most often defined based on direct measurement on a three‐dimensional plant representation in controlled conditions vs inversion of a model of light interception based on the gap fraction in the field (the gap fraction is the proportion of area seen as sky in pictures from below, as in Fig. 3b). Whereas the progression of phenological stages is similar for a given genotype in field and controlled conditions (Fig. 2a,b), the progression of leaf area measured either directly (controlled conditions, Fig. 2c) or via the gap fraction (field, Fig. 2d) showed markedly different temporal patterns. This illustrates the fact that mapping ontologies in field and controlled conditions is not only a question of standardization, but requires a theoretical study for linking concepts, in particular with the involvement of other traits such as plant architecture in the case of leaf area. Hence, an information system for both field and controlled conditions needs to optimize somewhat contradictory requirements; namely, using ontologies and methods that can be used for both types of datasets, taking into account the difficulties mentioned earlier, while keeping the interface sufficiently simple for the user.

The simplicity issue has been resolved by the design of the interface that automatically redirects menus and functionalities depending on whether the experiment is performed in the field or in controlled conditions. The difficulties associated with the commonality of tools, ontologies and workflows are therefore kept in the background. They are addressed with tools examined further in this paper.

### Tracking all objects in phenotyping experiments via object identification and ontology description

Tracking all objects involved in an experiment may seem unnecessary in simple experiments where unique correspondences exist between, for example, each plant and its position in a glasshouse or between each genotype and a plot in the field. In our own experience, automatic tracking is essential when thousands of plots, plants or sensors are dealt with. If not specifically reported in the information system, the replacement of a sensor at a given position (e.g. meteorological sensor or soil tensiometer) is not obvious in the outputs of an environmental database. In glasshouse experiments, a plant can be replaced by another plant at the same position and vector (e.g. pot, cart) during an experiment, potentially generating confusion. Because each sensor has its own calibration, each pot has intrinsic characteristics (weight, volume, age) and each position in the glasshouse or in the field has its local environmental conditions, it is crucial to track these objects and their relationships. All objects, therefore, need to be identified in order to keep the necessary information associated with them (e.g. positions over time, successive calibration for sensors, origin for plants). For example, in Fig. 3(a), a plant, a pot, a vector (here a cart placed on a conveyor belt) and a given *x*–*y* position need to be considered independently for keeping track of possible events in a glasshouse experiment. In the same way, tracking a specific organ in a field experiment involves a leaf belonging to a plant within a plot (Fig. 3b).

**Figure 4:**
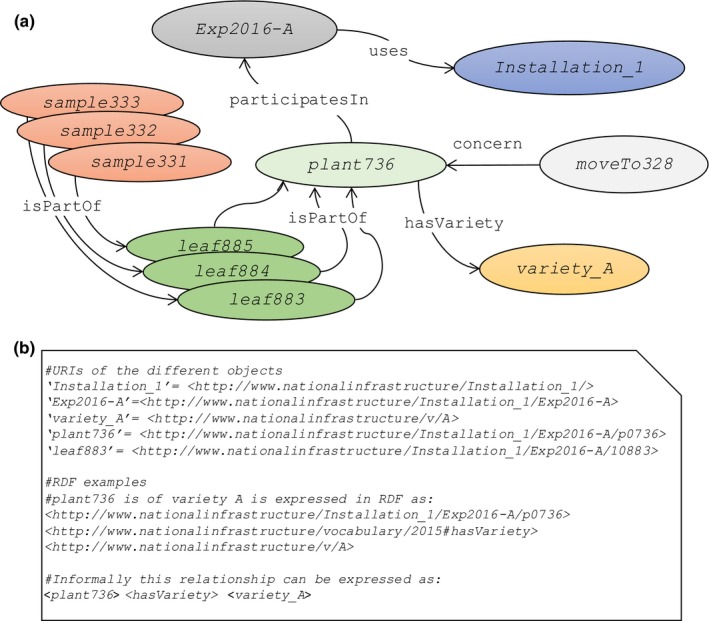
(a) Representation of a semantic graph describing the different objects involved in a phenotyping experiment and their relationships, using ontologies and semantics. (b) The relational instances between objects are formalized via semantic links using the resource description framework (RDF) data model that uses the triple data entity ‘subject–predicate–object’. Elliptical boxes are the objects represented as instances, and arrows represent the relational instances formalized in ontologies. Boxes with different colours represent different object types. Note that this is a simplified representation for relations and names of objects (e.g. names of objects presented with labels instead of unique resource identifiers (URIs)). Full URIs of objects and RDF examples are exemplified in (b).

In PHIS, object identification is based on a URI, which is a strings of characters used to identify an object in an unambiguous way (Fig. 3). This ensures traceability in space and time, whilst a typical identification by numbers (e.g. ‘plant 736’) refers to different plants in different experiments and installations. In the glasshouse experiment described here, the plant 736 in installation1 has the URI *<* http://www.Nationalinfra/Localinfra_a/Installation1/2017/c17000736 *>* (Fig. 4). The plot 206 in the field experiment located in installation2 has the URI *<* http://www.Nationalinfra/Localinfra_b/Installation2/2017/017000206 *>*. In these examples, URIs share the same prefix because installation1 and ‐2 belong to the same national infrastructure *<* http://www.Nationalinfra/>, followed by the identification of the local infrastructure *Localinfra_a* or *_b*, the installation considered and then the year, experiment and plant or plot identification (see PHIS Vocabulary menu for standardized definitions of these terms). URIs can be accessed from any web service client, thereby allowing the different objects involved in each experiment to be unambiguously and specifically identified (i.e. installation, plot, plant, plant organ, plant sample, sensor, variable).

### Links between objects, between events and between traits via semantic graphs and ontologies used in PHIS

The relations between objects need to be represented adequately in a high‐throughput context. For instance, if thousands of sample tissues have been collected on different leaves of different plants, the information ‘sample 884 belongs to the leaf 7 of plant 736’ may be lost if kept in a spreadsheet. The same occurs for the information that plant 736 has been moved from the position (*x*_1_, *y*_1_) to (*x*_2_, *y*_2_) during the experiment, making it impossible to connect this plant to local environmental conditions it has experienced over time. Semantic graphs (Berners‐Lee *et al*., 2001) allow automatic retrieving of this information (Fig. 4). The innumerable combinations of objects and events during an experiment are represented with parsimonious information, based on transitivity. For example, the notions ‘samples 331 to 333 belong to plant 736’ and ‘samples 331 to 333 have been collected on individual leaves 883 to 885’ are represented via a single predicate <*isPartOf*>. Because of the transitivity in semantic graphs, the system connects these samples to all objects already connected to plant 736. For instance, the information that samples come from an experiment of 2016 in *Installation1* belonging to a national infrastructure and that they belong to a plant of variety A are automatically retrieved via the predicates <*participatesIn*> and <*hasVariety*> respectively (Fig. 4). The information that the sample comes from a plant that moved from position (*x*_1_, *y*_1_) to (*x*_2_, *y*_2_) on day *i* is automatically retrieved via the link to plant 736, itself related to an event via the predicate <*concerns*> and the subject <*moveTo328*> that provides the date, site and old and new positions in the glasshouse.

**Figure 5:**
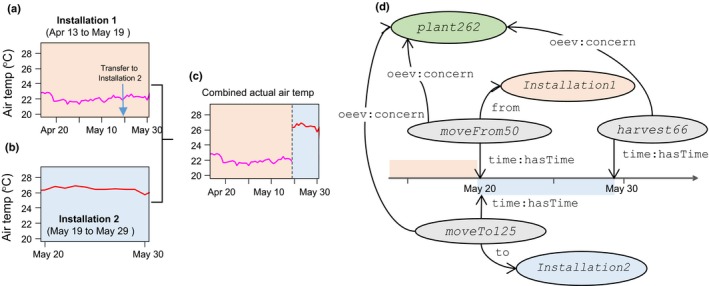
Example of object tracking using the Ontology for Experimental Phenotypic Objects (OEPO) and Ontology of Experimental Events (OEEv) ontologies for following plant 262 over time in two installations. (a) Air temperature conditions in Installation 1 where biovolume is measured from 13 April to 19 May. (b) Air temperature conditions in Installation 2. (c) Air temperature sensed by plant 262 resulting from combining air temperatures of Installations 1 and 2. (d) Associated informal semantic graph representing such event. Boxes with different colours represent different object types (green for plant material, orange and blue for installations and grey for events). Names in this figure are neutral for better legibility. Corresponding names for consulting the online information system are ‘phenoarch’ for ‘installation1’ and ‘phenodyn’ for ‘installation2’.

The links between objects in Fig. 4 are based on two application ontologies proposed here, and compliant with the standards of OWL. The Ontology for Experimental Phenotypic Objects (OEPO) describes objects involved in phenotyping experiments (e.g. infrastructure, devices, germplasm, scientific objects) and defines specialization hierarchy between them according to the specificities of the installations and experiments (see Notes S7 for technical details). The Ontology of Experimental Events (OEEv), characterizes events that occur during an experiment; for example, moving of plants, dates of sowing, application of a given treatment, harvesting, measurements or sampling for ‐omic measurements, or any category of technical problem (see Notes S7). For instance, the *Trouble* concept distinguishes *Breakdown* (sensor or conveyor), *Dysfunction* (sensor fault, irrigation trouble) and *Incident* (a pot falls down, a leaf is blocked in an imaging cabin, lodging of a plot, human error, etc.). As described in the associated semantic graph (Notes S7), an event can be associated with objects (e.g. plant, plot, sensor) and with the user who has annotated the event, and the occurrence date can be tracked along with every relevant detail. This information can be retrieved, plotted on graphs and used, for instance, to detect anomalous data or to calculate new variables. For example, plants are often transferred, during a single experiment, between installations or compartments with different environmental conditions. Fig. 5 represents a case in which plant 262 is sequentially monitored in two installations over 50 d (Fig. 5a,b). The experiment takes place in Installation 1 for daily measurements of biovolume and transpiration, and then temporarily moved to Installation 2 for 10 d for more precise measurements (19–29 May, blue area in Fig. 5a,b), before being harvested. Environmental conditions sensed by plants differ between the two installations, so a proper tracking tool is essential. In our experience, manual tracking is extremely tedious if environmental, phenotypic and management data are spread in distinct databases, lab books or individual text/spreadsheet files, especially when different groups are involved. As shown in the associated semantic graph (Fig. 5d), tracking the presence of the plant 262 in Installation 2 can be formalized to automatically link environmental data associated with a given plant in each installation, without the need to specify the plant location at a given time (Fig. 5c). The full example together with technical information is provided in Notes S3.

It is noteworthy that the PHIS‐specific OEPO and OEEv application ontologies formalize the installation entities and allow dynamic configuration of PHIS. These ontologies refer to objects and events that can be specific to either field or controlled conditions. The mapping of ontologies between types of installations can be simple; for example, the *x*–*y* position of a plant in a glasshouse exactly corresponds in the field to GPS coordinates. Other objects and events can be specific; for example, a plant may fall in controlled conditions, while lodging can occur in the field. In this last case, there is no need to establish a correspondence between events. Hence, OEPO and OEEv application ontologies involve objects and events that are common to all installations, whereas other objects and events are specific to one category of installations and need to be defined by the groups that drive these installations, but also require coordination and standardization between installations. The (precise) ontologies OEPO and OEEv have been mapped whenever possible to existing ontologies; for example, the Ontology for Biomedical Investigations (Bandrowski *et al*., 2016), the Plant Experimental Conditions Ontology (http://purl.bioontology.org/ontology/PECO), the Plant Ontology (http://plantontology.org/; Ilic *et al*., 2007; Walls *et al*., 2012; Cooper *et al*., 2013), the Plant Phenotype Experiment Ontology (http://purl.org/ppeo), and others such as the AGROVOC (Caracciolo *et al*., 2013), the Relations Ontology (Smith *et al*., 2005) and the Semantic Sensor Network Ontology (http://purl.oclc.org/NET/ssnx/ssn). The FAO/Bioversity Multi Crop Passport Descriptors is also used for germplasm identification (Alercia *et al*., 2015; Yeumo *et al*., 2017).

**Figure 6:**
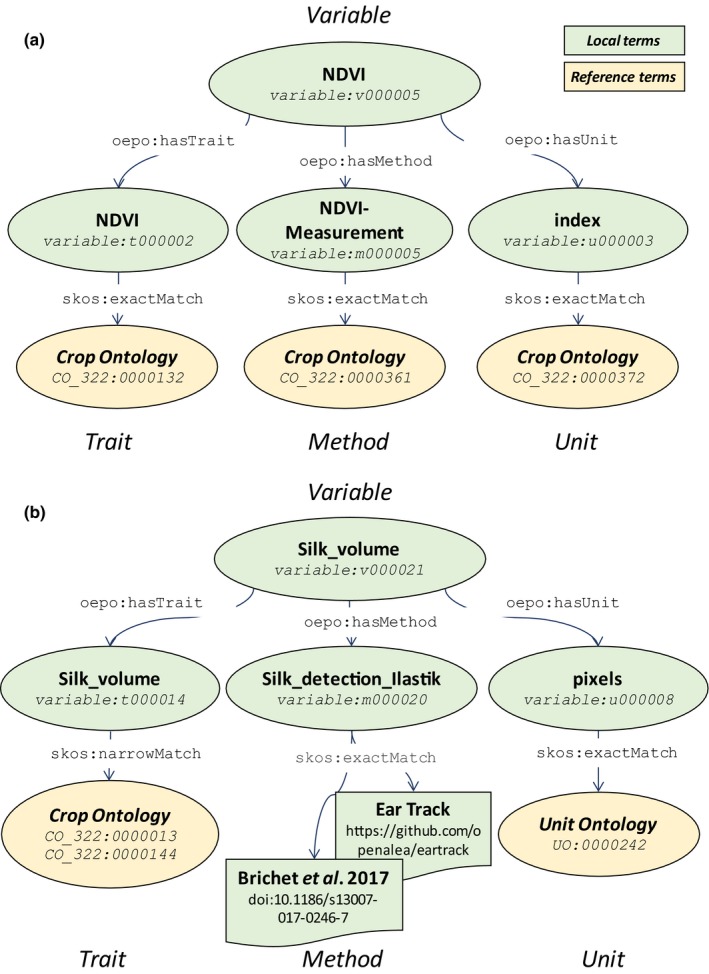
(a) Example of description in the Phenotyping Hybrid Information System (PHIS) of the widely used and standardized term ‘normalized difference vegetation index’ (NDVI) using the Crop Ontology ‘trait–method–unit’ data model and ontology mapping. (b) Description of the local term ‘Silk_volume’ derived from maize ear images captured in the glasshouse in PHIS using ontology mapping and local methods. The prefixes *oepo:*,* skos:* and *variable:* preceding the unique resource identifiers stand for *<* http://www.phenome-fppn.fr/vocabulary/2018/oepo *#>*, *<* http://www.w3.org/2004/02/skos/core *#>* and *<* http://www.phenome-fppn.fr/m3p/variable *#>* respectively.

The same applies to trait ontologies that can either be mapped on existing ontologies for some traits (Cooper *et al*., 2018), or need more complex correspondence for others (Fig. 2). For instance, well established and standardized traits such as the canopy normalized difference vegetation index, as well as the method and the units used for this term, are referenced in PHIS using the standards defined in the Crop Ontology (CO_322:0000880) (Fig. 6a). On the contrary, local terminologies exist in PHIS to deal with the specificities of each installation. For instance, the local term *Silk_volume* derived from maize ear images captured in the glasshouse (Brichet *et al*., 2017) is related to two existing ontological terms, *silk growth* and *silk length*, defined in the Crop Ontology CO_322:0000144 and CO_322:0000013, as well as to *pixel* units of measurement described in the Unit Ontology UO_0000242 (Fig. 6b), whereas it has been measured using local methods not referenced in existing ontologies (Fig. 6b). Mapping for traits, methods and units is done in PHIS, for example, to the Crop Ontology (http://cropontology.org/; Shrestha *et al*., 2012), the Plant Trait Ontology (http://www.obofoundry.org/ontology/to.html), the PATO (http://www.obofoundry.org/ontology/pato.html) and the Unit Ontology (http://www.ontobee.org/ontology/UO).

We are aware that considerable work remains to be done for connecting the ontologies of traits, objects and events used in different installations. The correspondences between field and controlled conditions presented earlier were simplified by the fact that the groups involved exchanged for years on a day‐to‐day basis. The mapping of ontologies implemented here is based on the premises that not all terms used at one scale can map to another term at another scale, and that it is almost inevitable that a common term is used with different meanings by different communities. Several initiatives work on this problem; for example, the MIAPPE initiative (http://www.miappe.org/), with which PHIS works intensively (Krajewski *et al*., 2015; Ćwiek‐Kupczyńska *et al*., 2016). Until this effort is fully successful, a pragmatic approach needs to be used. This may be at the cost of a transitory lack of correspondence between terms used in different installations.

### Data annotation

**Figure 7:**
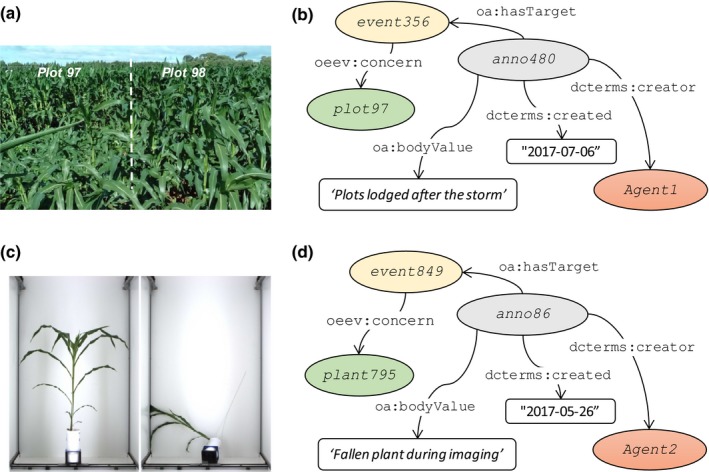
Examples of data annotation in the Phenotyping Hybrid Information System for describing (a) a lodging event occurring in the field (*installation1*) and (c) an accident during image acquisition in the glasshouse (*installation2*). (b, d) Informal semantic graphs representing the information associated with each event (users, installations, plots, plants, description) using the Ontology of Experimental Events ontology and the web annotation data model. Names in this figure are neutral for better legibility. Instances, classes and literals are depicted as coloured ellipses, white rectangles and white lozenges respectively. Relationships and properties are depicted as black lines. Note that this is a simplified representation. Corresponding names for consulting the online information system are rchapuis for ‘Agent1’ and ‘lcabrera’ for ‘Agent2’, ‘diaphen’ for ‘installation1’ and ‘phenoarch’ for ‘installation2’.

Classically, events occurring during experiments are recorded in laboratory notebooks so they are hardly available for real‐time monitoring and data interpretation. For instance, the annotation *plot 110 is chlorotic* is of nearly no use in a high‐throughput context if recorded in a notebook: it took a full week for one person to review all annotations in notebooks and spreadsheets corresponding to one experiment (S. Alvarez Prado, pers. comm.). This tedious work is most often omitted, so annotations are finally not taken into account. The semantic annotation allows linking additional information (relation, comment, document, etc.) to objects (e.g. plot, plant, plant organs, sensors, experiments) so the user can visualize data or images associated with events or annotations in order to take them into account in data analysis. For instance, declared incidents may include technical problems related to cameras or mechanical problems, or events related to the management of the experiment or environmental conditions. Fig. 7(a) illustrates the lodging of a plot after a heavy rain, and Fig. 7(c) illustrates a plant fallen during the acquisition of images in the imaging cabin in the glasshouse. As presented in Fig. 7(b,d), users 1 and 2 declared the incidents *event356* and *event849* that involved several plots or plants. A description of the event (text, image or video) can be attached; for example, ‘Plots lodged after the storm’ (Fig. 7b). This allows the user either to request the characteristics of a given plant or to request the list of all plots or of all leaves originating from plots that have suffered lodging in the experiment. The full example together with technical information is described in Notes S2.

### Expert annotation during data analysis

**Figure 8:**
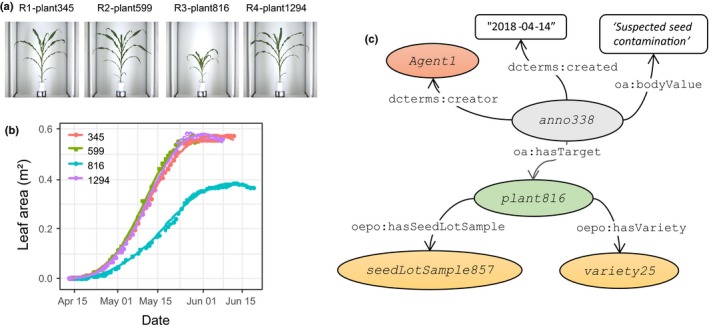
Example of expert annotation using the Ontology of Experimental Events ontology. (a) RGB side images of four replicates of the same genotype in a common experiment. (b) Plant leaf area curves resulting from image analysis of these four plant replicates. (c) Associated informal semantic graph that shows how an expert user *Agent1* declares an expert annotation *Annotation338* that concerns the detected anomalous plant *plant816*. Instances, classes and literals are depicted as coloured ellipses, white rectangles and white lozenges respectively. Relationships and properties are depicted as black lines. Names in this figure are neutral for better legibility. Corresponding names for consulting the online information system are lcabrera for *Agent1*, and *<* http://www.phenome-fppn.fr/m3p/arch/2017/c17000816 *>* for *plant816*.

Some anomalies can only be detected at the data processing step. For instance, a plant supposed to belong to a given genotype might grow much more slowly than its replicates. This can be automatically pointed out by classical clustering methods or annotated manually after expert user validation (Bernal‐Vasquez *et al*., 2016). The latter is shown in Fig. 8, where a suspicious plant (probably related to seed contamination) is detected out of the four replicates of the same genotype in a common experiment after a visual analysis of either images (Fig. 8a) or growth curves (Fig 8b). Clicking on the curves in Fig. 8(b) allows the displaying of images of the four replicates. In this case, the user can see that the third replica, in addition to having smaller leaf area, presents a different architecture, thereby making it still more suspicious. Data annotation associated with specific algorithms is therefore a help to data cleaning.

As described by the semantic graph presented in Fig. 8(c), the expert user declares an annotation *Annotation338* that concerns the detected anomalous plant *plant816*. The OEPO and OEEv ontologies allow tracking this event by going backwards in order to find the origin of the incident, which may be related to seed contamination (Fig. 8c). In this example, the seeds used for this experiment came from a particular seed lot (*seedLotSample857*), generating the suspicion that other errors may have occurred in the same seed lot. The information associated with this seed lot is gathered in the information system via the web interface. More generally, images, videos and media files associated with any event can be uploaded to the information system for further interpretation of phenotyping data. The full example, together with technical information and further examples, is provided in Notes S2.

### Advanced data querying, data visualization and scientific computing through interaction between the different component layers of PHIS

**Figure 9:**
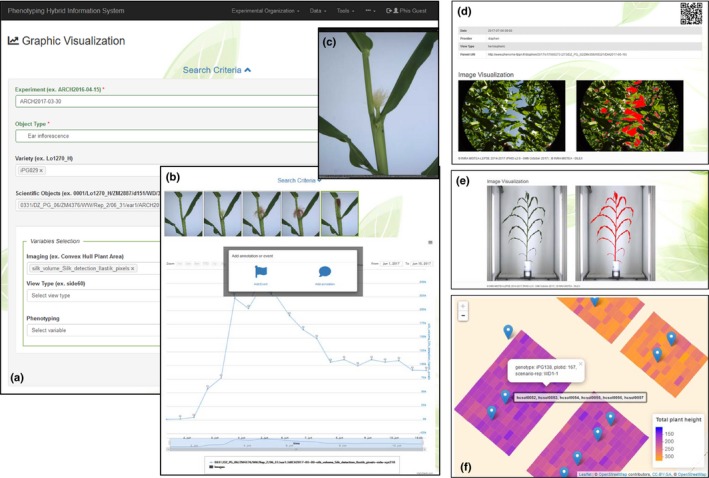
Examples of advanced data visualization. (a) Data querying for dynamic visualization of traits resulting from image analyses based on different filters (object type, varieties, plants, image angles). (b) Interactive graphics for exploring dynamic variables over time (silk volume and image thumbnails) and annotating points with comments and events. (c) Pop‐up of an ear image corresponding to a particular data point in the graph. Raw images, segmented images and metadata can be displayed in both (d) field and (e) glasshouse experiments. (f) The interaction with the knowledge layer allows projection of variables using GPS coordinates associated with plants or plots and sensors in field experiments.

The user may want to retrieve ‘plants with leaf area higher than 0.6 m^2^ and/or plant height higher than 1500 mm’, ‘images of plots having suffered lodging’ or ‘show all sensors that display temperatures higher than 40°C’ (Notes S4). This is accomplished by using the inference engine based on the semantics and rules represented in the OEPO and OEEv ontologies, thereby linking the knowledge stored in the Triple Store to the information distributed among the different storage systems. Interaction between different layers of PHIS (Fig. 1) also provides advanced visualization features for displaying images, dynamic graphs of static or time courses of phenotypic and environmental variables that are automatically adapted to the particular experimental settings, and variables such as glasshouse (Fig. 9a) or field (Fig. 9d). For instance, the user may request a dynamic visualization of image analyses and watering results based on different filtering options (image angles, genotypes, plants, treatments; Fig. 9a). Such interactive figures allow the exploration of dynamic variables over time (e.g. plant area or water use). Graphs can be zoomed into a particular time window: clicking on a data point automatically displays the images associated with this point together with the associated annotations (Fig. 9b,c). Raw images, segmented images and metadata can be displayed in both field (Fig. 9d) and glasshouse experiments (Fig. 9e). The interaction with the knowledge layer allows the projection of variables using GPS coordinates associated with plants or plots in field experiments (Fig 9d) and linking data with environmental sensor outputs.

PHIS includes extensible scientific computing modules based on R packages for calculating elaborated variables and generating experimental summaries and reports. Automatic reporting is based on data query through the interface and R integration in text processing (R Markdown). Basic reports include daily, weekly or final overviews of experiments, with standard statistics and graphic visualization of averaged queried traits. Extended reports may include specific calculation of traits and environmental conditions associated with a given plant or genotype. In particular, new variables can be calculated for a given experiment based on the joint use of phenotypic and environmental data together with associated metadata to plants and traits (e.g. events, methods). For instance, the progression of thermal time for each individual plant or plot can be calculated taking into account the local air or leaf temperatures, the dates of sowing and the method to perform such calculation (see Notes S4).

### Integration of external data and interoperability with external installations and resources

The dialogue with external applications and information systems is managed through the web service API that allows integration of data from external databases and resources, export to computing and modelling platforms and integration of phenomic data into other systems. Web services provide flexible and powerful capabilities for the integration of a diverse and multi‐source amount of data, including structured environmental and phenotypic data acquired by the different sensors of the installation (stored in a PostgreSQL database), the images stored in the iRODS system, nonstructured data such as elaborated variables stored in the MongoDB, and rich metadata and knowledge stored in the Triple Store (Notes S6). The versatile use of web services allows one to virtually integrate data from any external client, therefore allowing PHIS to be adapted into other infrastructures. For instance, environmental data from a network of field meteorological stations (http://w3.avignon.inra.fr/carto/) and soil sensors (http://www.agriscope.fr/) are integrated in PHIS via the web service API. Similarly, features extracted from image analyses via Python are routinely integrated in PHIS through the web services, and an R client allows different analysis pipelines (Notes S6).

PHIS can also export data to external databases, in particular those dedicated to genetic analyses or modelling. For example, export to the GnpIS information system (https://urgi.versailles.inra.fr/gnpis; Steinbach *et al*., 2013), a member of the ELIXIR European infrastructure, allows genome‐wide association studies based on the phenotypic datasets organized in PHIS via the collaborative Breeding API. Export to the modelling platform OpenAlea (Pradal *et al*., 2008, 2015) has allowed calculation of the light interception and radiation‐use efficiency of hundreds of maize plants using data obtained in the M3P installation (Cabrera‐Bosquet *et al*., 2016). Finally, data search and advanced queries can be performed to remote databases thanks to the web service API and the inference engines that use the semantics and rules represented in the ontologies. These import and export APIs facilitate the interoperability and data sharing capabilities in the context of Open Data Science (Halewood *et al*., 2018); for example, in the context of European projects EPPN^2020^ (https://eppn2020.plant-phenotyping.eu/) Trans‐PLANT (http://transplantdb.eu/), ELIXIR‐EXCELERATE (https://www.elixir-europe.org/excelerate/plants) or EMPHASIS (http://emphasis.plant-phenotyping.eu/).

Taken together, the functionalities present in PHIS may allow assembling a number of datasets from different installations in controlled and field conditions (including phenotypic, environmental and contextual information), resulting in an unprecedented amount of information that can be reused, combined and reanalysed to generate new knowledge. This can be of particular interest for covering the necessities of most phenotyping installations not having the appropriate tools for storing, organizing and managing phenomic data, as well as for data management strategies for networks of installations. Nevertheless, PHIS can also be adapted to local software and databases and used as a mapping layer enabling interoperability between information systems.

## Author contributions

P.N., A.T., N.H., F.T. and L.C‐B. planned and designed the research, A.T. designed the software architecture and J.M‐C., V.N., I.S. and N.B. contributed to software development, C.P. tested web services, L.C‐B. and R.C. performed the experiments, and L.C‐B., R.C. and N.B. analysed data. P.N., A.T., N.H., B.C., F.T. and L.C‐B. wrote the manuscript.

## Supporting information

Please note: Wiley Blackwell are not responsible for the content or functionality of any Supporting Information supplied by the authors. Any queries (other than missing material) should be directed to the *New Phytologist* Central Office.

**Notes S1** Experimental Organisation menu of PHIS web user interface.

**Notes S2** Annotations and events.

**Notes S3** Object tracking menu of PHIS web user interface.

**Notes S4** Data menu of PHIS web user interface.

**Notes S5** Tools menu of PHIS web user interface.

**Notes S6** Web Service API.

**Notes S7** OEPO and OEEv ontologies and annotations.
