# Supplementary material for: Dealing with multi‐source and multi‐scale information in plant phenomics: the ontology‐driven Phenotyping Hybrid Information System
Source: New Phytol. 2018 Aug 28;221(1):588–601. doi: 10.1111/nph.15385 (PMC6585972; doi:10.1111/nph.15385)
Supplement: Supplementary file 1 — Notes S1 Experimental Organisation menu of PHIS web user interface. [file NPH-221-588-s001.pdf]

Experimental Organization

The Experimental Organisation menu contains information about the experimental resources including projects, experiments, installations, plants, germplasm, devices, events, phenotypic and environmental variables and object tracking tools.

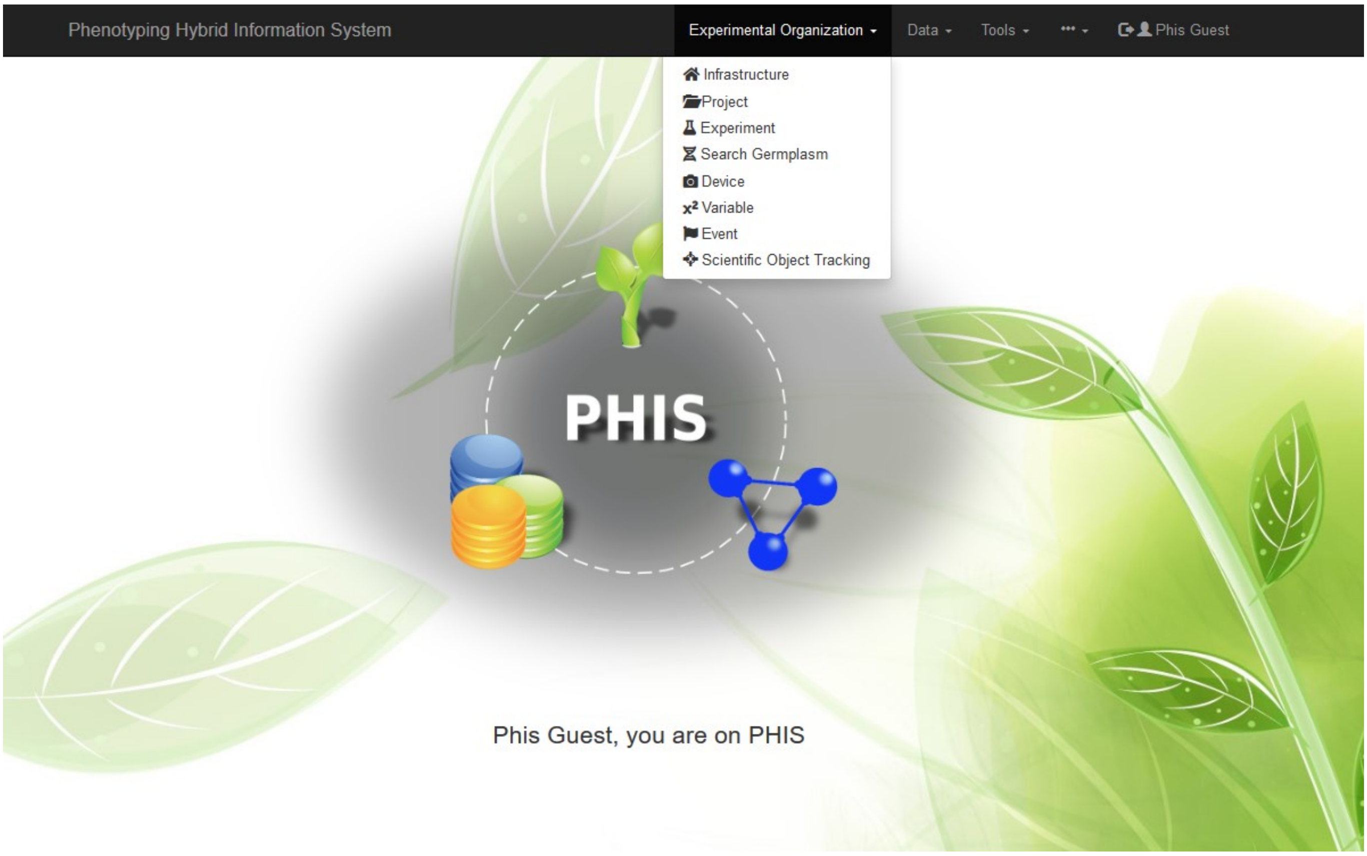

Projects

What are projects?

A research project is the higher level of organisation in PHIS. It is generally the object of a contract or a grant agreement between the laboratory performing the research activities and funding agencies providing the financial support. A project can hold many experiments from various experimental campaigns. Besides, several projects can participate to the same experimentation.

What are project properties?

Projects fact sheets are all public, i.e. visible for every PHIS user, including those unrelated to them. However, experimental data included in a project is also necessarily related to a given experiment. This data is accessible only to users who belong to a group (either owner or guest) authorized to access the experimentation it is related to: access rights are managed by experiment and not by project. Please see the **Restrictions to a group** section of this documentation for further details on data access matters.

The complete list of projects is available in the `Projects` menu, accessible from PHIS top navigation bar. The projects list is preceded by a research bar enabling the user to filter projects by their acronym, their main source of funding, and their start and end dates. There's no need to use the same case as the expected results: a lowercase search returns every results matching the characters, disregarding whether those match

are lowercase or uppercase. Further information on a given project is available on its information sheet, accessed to from the projects list through the eye-icon on the right-hand side of this project row.

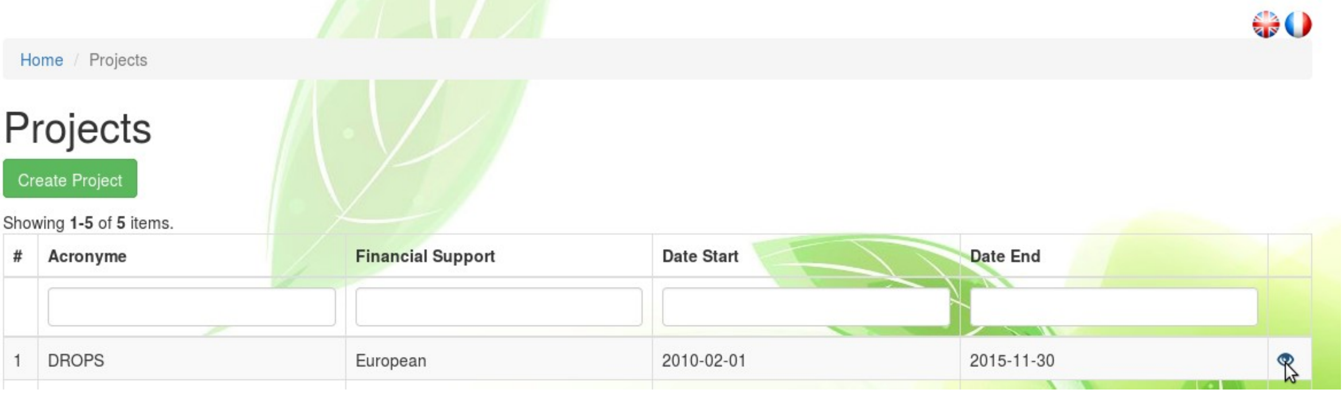

Create a project

Within the `Projects` menu, one can create a new project with the `Create Project` button. Tool tips are provided for most fields: they appear on the left hand side when one drags the pointer on those fields.

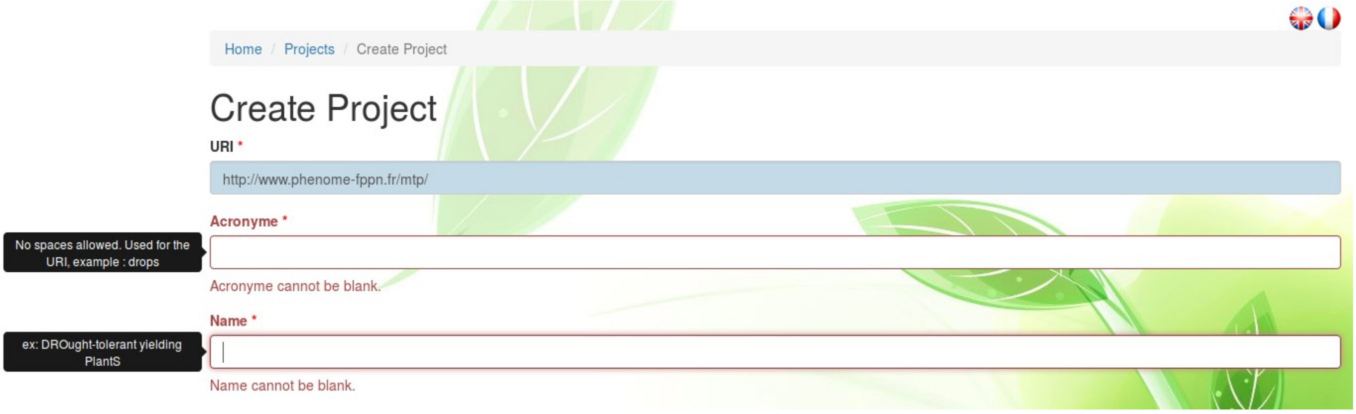

The mandatory fields, followed by a red asterisk, are:

- acronyme
- name
- date (start / end)

However, it is highly recommended to fill every field. If possible, fields requiring plain text (keywords, description) should be answered to in English.

`URI`. The project URI is automatically created using the acronym provided by the user. The URI creation respects the **w3 recommandations**.

`Acronym`. Short and meaningful, the acronym will be the preferred way to refer to the created project. The acronym provided here is usually the one of the research project. A project acronym cannot include spaces. Once the project created, its acronym can no longer be modified.

`Name`. Full name of the project, it will seldom be refered to but allows a project creator to explicit the acronym. For instance, *RAPeSeed Oil content anD Yield under low Nitrogen input* would be an adequate name for the project whose acronym is *RAPSODYN*.

`Subproject Of`. If the new project is part of an existing project (requisitly known to PHIS), one can select the name of the given mother-project within the exhaustive list of projects registered to the PHIS instance where the new project is created.

`Subproject Type`. The subproject type can optionnaly be provided. It can be either an existing type to be selected from the list of proposed project types, either a new type provided by the user. In the later case, one has to type the new subproject type (e.g. *phd thesis* or *internship*) in the search field of **Subproject Type** and then click on the newly proposed type.

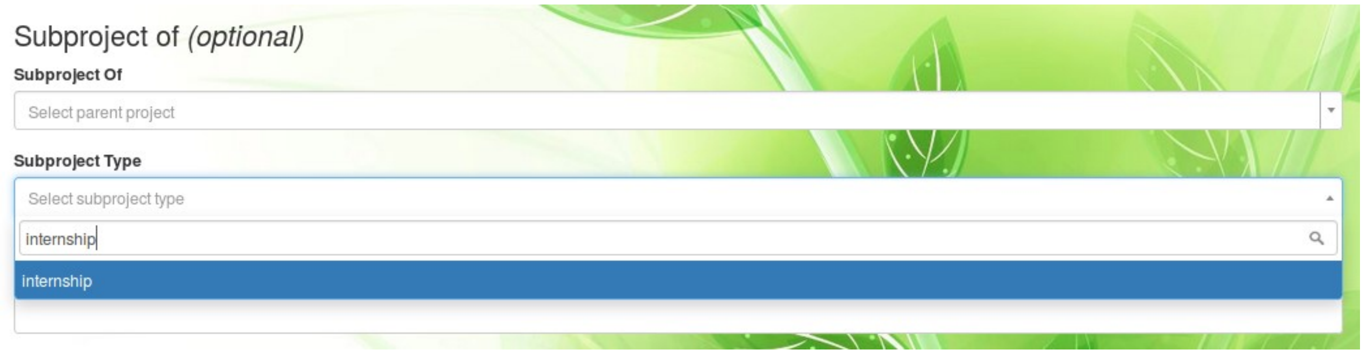

`Objective`. A short synthesis of the project objectives is to be provided in plain text, preferably in English. A more complete description will be asked in the **Description** field.

`Financial Support`. Organism, institution or funding agency providing the financial support of the research project. Only one organism supporting financially the project can be cited for now. Same as the **Subproject Type** field, the answer can be both from a predefined list or a new one (the user has to type the new organism name and then click on the search result matching the organism name).

`Financial Name`. As indicated in the tool tip, this field is meant for the user to provide the identification of the financial support document (e.g. a grant agreement number).

`Date Start` and `Date End` are to be provided in the format YYYY-MM-DD (year-month-day), directly or through the calendar view. If the end date of the project is unknown, the start date can be used also as **Date End** temporarily.

`Scientific Contacts`. Email adresses of the scientists involved in the project. The email adresses refer uniquely to persons existing on PHIS. If unavailable in the predefined list, emails can be added to PHIS from the `Persons` menu, prior to the new project creation. Please see the **Persons** section of this documentation for further details.

`Administrative Contacts`. Email adresses of the administrative personnel linked to the project (e.g. human ressources people). The email adresses refer uniquely to persons existing on PHIS.

`Project Coordinators`. Email adresses of the project coordinators. The email adresses refer uniquely to persons existing on PHIS.

`Website`. If available, URL of the website of the project.

`Keywords`. Keywords, preferably in English, characterizing the project. Each keyword should be separated by a comma and not include any capital letter, e.g. *drought, maize*.

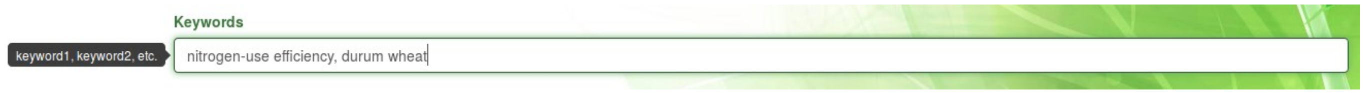

`Description`. Complete plain text description of the project, preferably provided in English language. Additionally to the description, additional information can be provided as uploaded files, after the project has been created.

Infrastructure

The Infrastrucutre menu contains information about the different Infrastructure including, `European`, `National` and `Local Infrastructure`, and information about the different `Installations`.

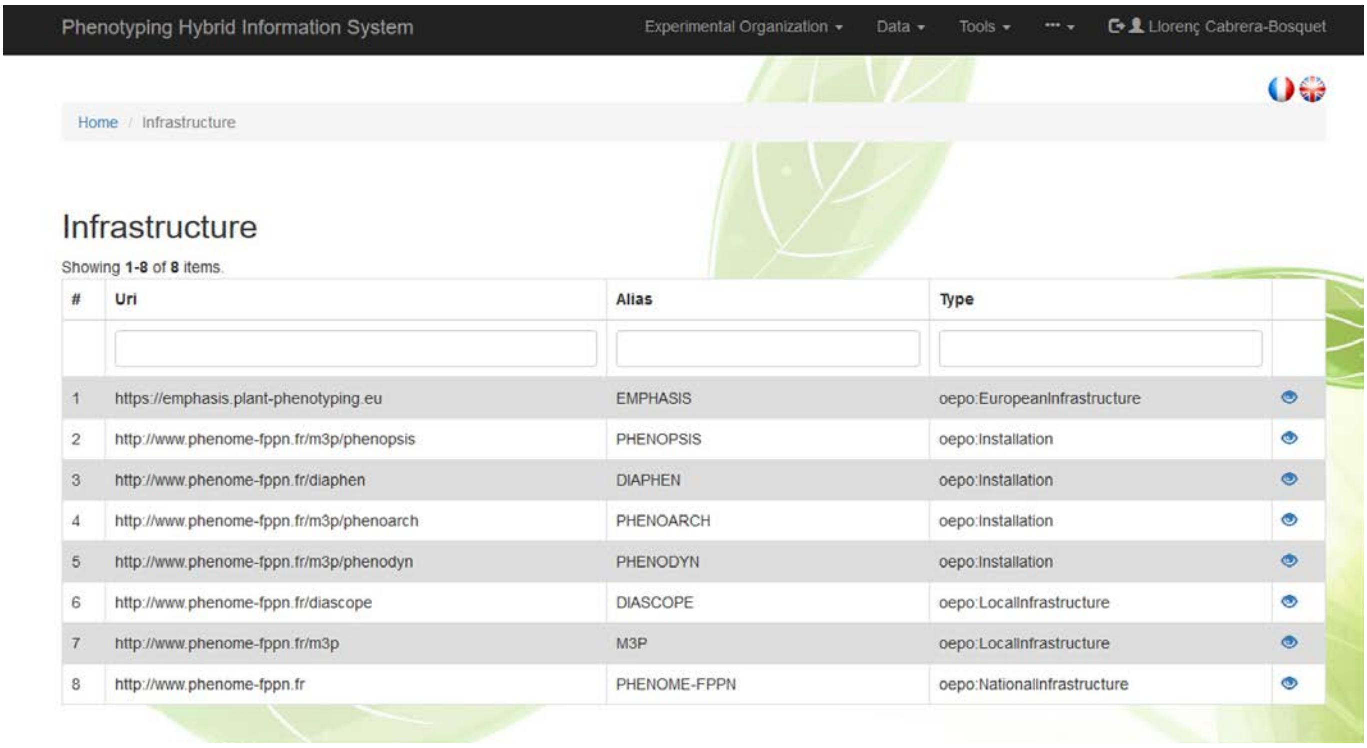

#	Uri	Alias	Type
1	https://emphasis.plant-phenotyping.eu	EMPHASIS	oepo:EuropeanInfrastructure
2	http://www.phenome-fppn.fr/m3p/phenopsis	PHENOPSIS	oepo:Installation
3	http://www.phenome-fppn.fr/diaphen	DIAPHEN	oepo:Installation
4	http://www.phenome-fppn.fr/m3p/phenoarch	PHENOARCH	oepo:Installation
5	http://www.phenome-fppn.fr/m3p/phenodyn	PHENODYN	oepo:Installation
6	http://www.phenome-fppn.fr/diascope	DIASCOPE	oepo:LocalInfrastructure
7	http://www.phenome-fppn.fr/m3p	M3P	oepo:LocalInfrastructure
8	http://www.phenome-fppn.fr	PHENOME-FPPN	oepo:NationalInfrastructure

Information associated to each infrastructure can be explored. For instance information about the Local Infrastructure M3P (http://www.phenome-fppn.fr/m3p) and events concerning this infrastructure can be browsed:

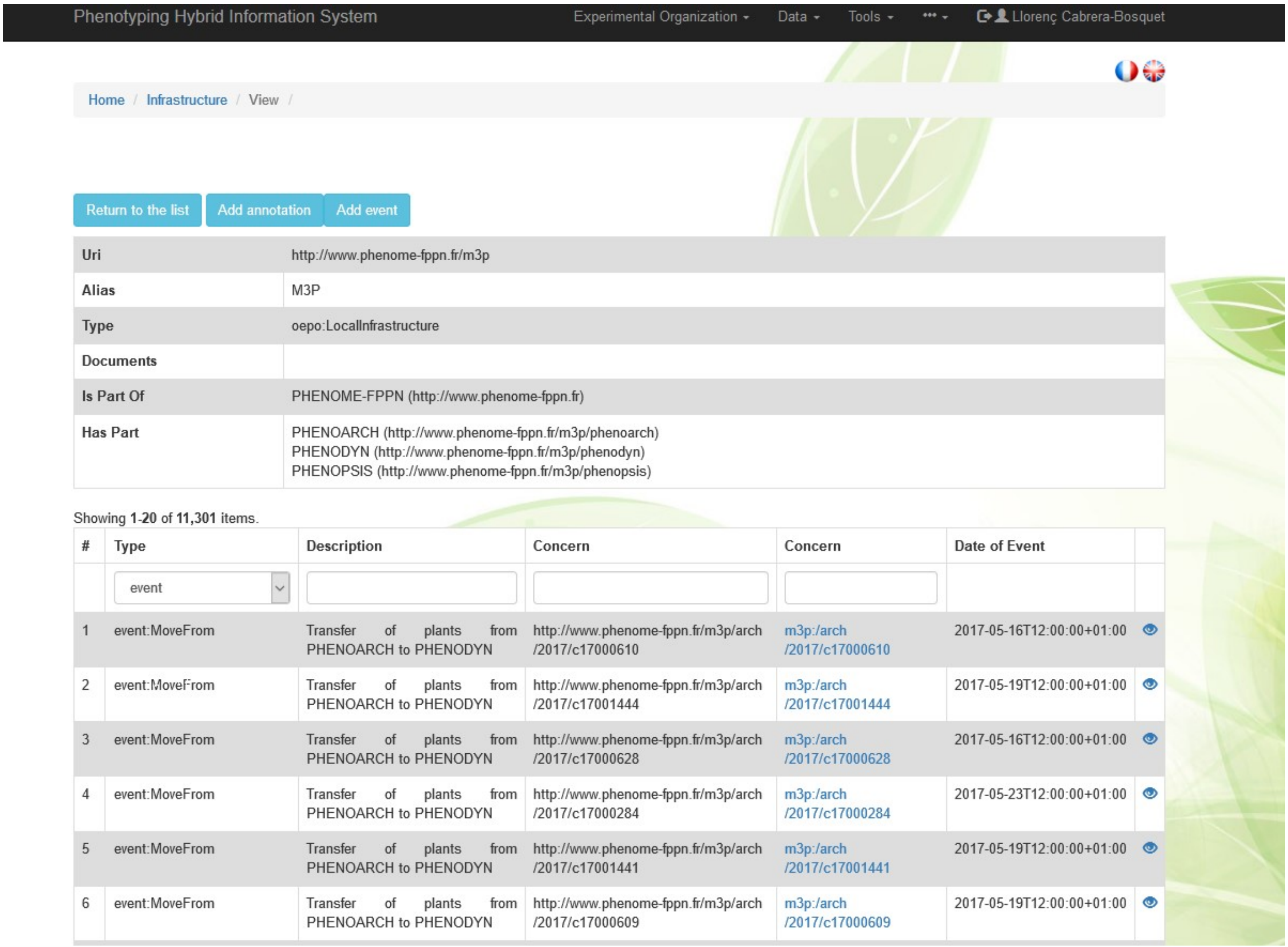

Annotations and events can be associated to eavh object by using the `Add annotation` and `Add event` buttons.

Experiments

What are experiments?

Field plant phenotyping experimentations are refered to in PHIS as **Experiments**. Experiments in PHIS form self-sustained organizational units occuring in a delimited and known time frame. Every scientific objects and environmental data stored in PHIS field have to be related to an experiment. Experiments include both raw and cleaned data. The data types found in PHIS experiments are including but not limited to :

- phenotypic data
- environmental data
- analysis, workflows and their associated results documents
- giving a deeper understanding of the experiments What are

experiment properties?

Contrary to projects, experiments information sheets are not public, but accessible only to the PHIS members which are part of the groups involved in these experiments. Please go to **Access rights** for further details on this matter.

From the `Experiments` menu, a PHIS user has access to the list of experiments it has the rights to access to. Every experiment on this list is characterized by :

- its **URI** which identies it uniquely its
- **Alias**, a short internal denomination
- its stat and end dates
- the experimental installation in which is has been performed
- the campaign it is part of, i.e. the year

From **Home / Experiments**, one can click on the eye icon on the right to see a given experiment information sheet.

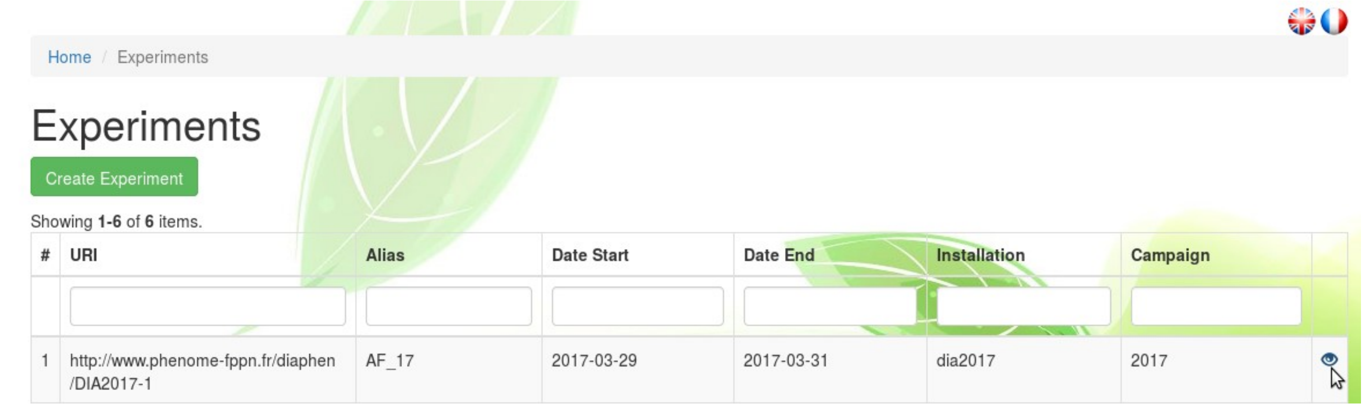

Further information on the experiment is provided on its information sheet, such as the groups of users that have been given access to the experiment's data. For more detail on experimental data access restrictions, see the section **Restrictions to a group**.

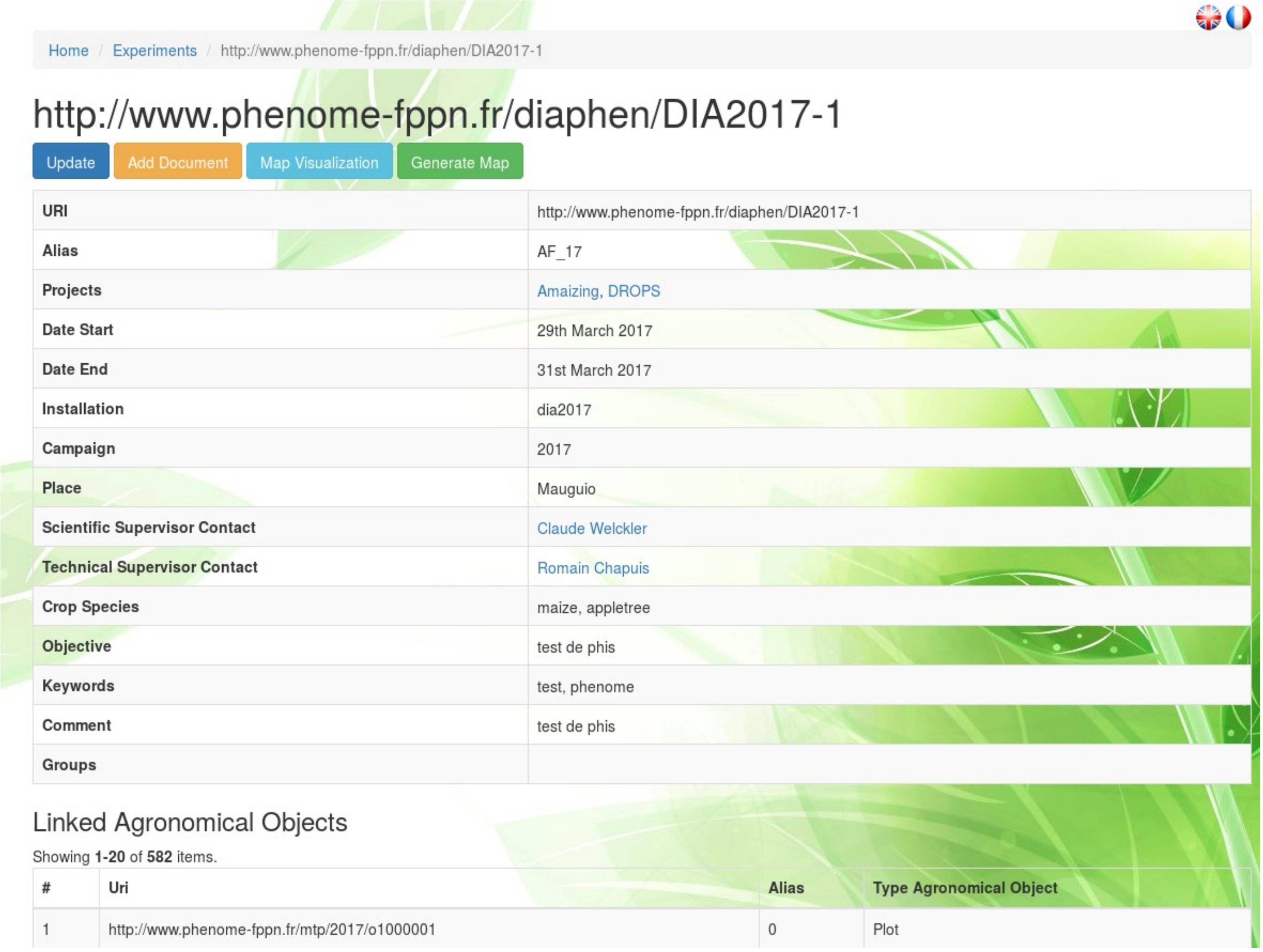

On top of the experiment information sheet, several buttons are displayed. The buttons `Map Visualization` and `Generate Map` enable the visualization of the scientific objects (e.g. plots) of the experiment on a map centered on the experiment intallation location. Jump to the section Map Visualization for more information

on experiment maps. The use of the other buttons, `Update` and `Add document`, is described at the end of the next section entitled Create an experiment.

Create an experiment

Within the `Experiments` menu, a PHIS user (not available for guests) can create a new experiment with the `Create Experiment` button.

Tool tips are provided for some fields: they appear on the left hand side when one drags the pointer on those fields.

The mandatory fields, followed by an asterisk, are:

- projects date
- (start / end)
- campaign

However, it is highly recommended to fill every field. If possible, fields requiring plain text (keywords, description) should be answered to in English.

`URI`. The project URI is automatically created by PHIS web service which uses the acronym of the PHIS instance from where the experiment is created (e.g. *DIA* for Diaphen) and the **campaign** provided by the user (e.g. *2018*).

`Alias`. Internal name of the experiment, usually provided in all caps.

`Projects`. Name of the project(s) the new experiment is part of. A single experiment can be linked to several projects. One can select the name of the given projects only within the exhaustive list of projects registered to the PHIS instance where the new experiment is created.

`Date Start` and `Date End` are to be provided in the format YYYY-MM-DD (year-month-day), directly or through the calendar view. It it compulsory to give a value to the **Date End** field in order to create an experiment. If the end date of the experiment is unknown, the start date can be reused in the **Date End** field. Typical **Date Start** values correspond to a sowing date, while **Date End** typically concur with an harvesting date.

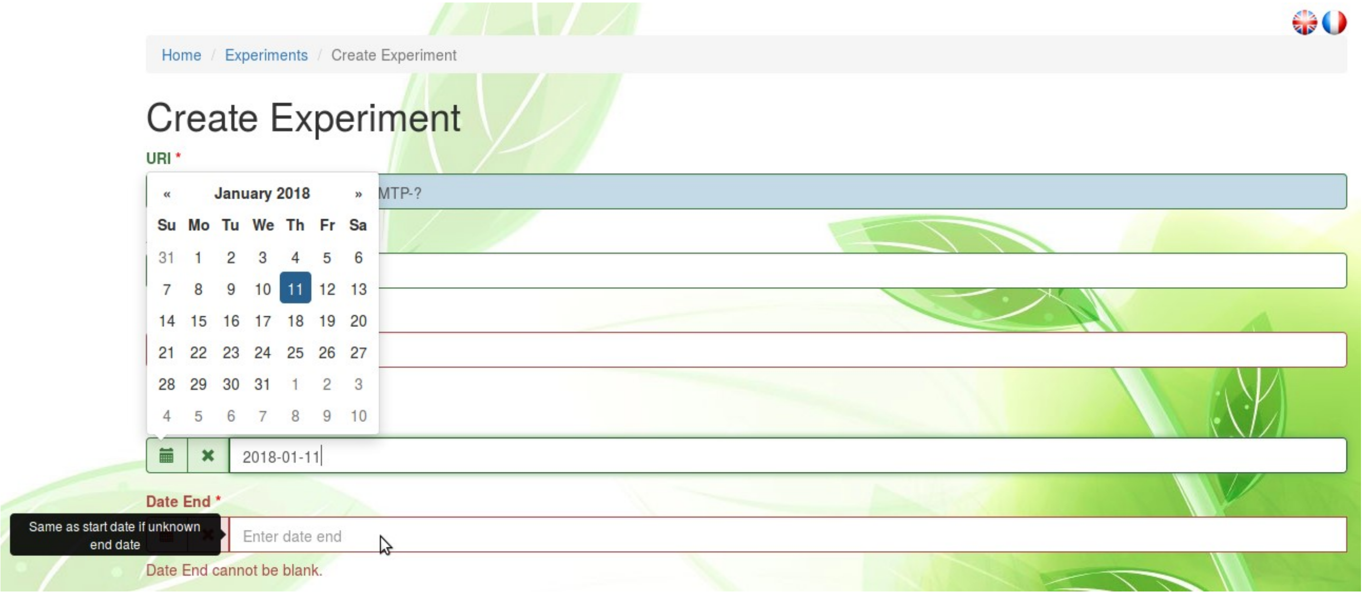

`Installation`. Name or ID of the installation where the experiment is carried. No specific format is yet required for submitting an installation name. *todo*.

`Campaign`. Year (format YYYY) in which the experiment has been carried, or the year of the harvest/end of the experiment in case it has been carried on several years (*to be confirmed*). Once the experiment created, the information on the campaign in which it has been carried can no longer be modified.

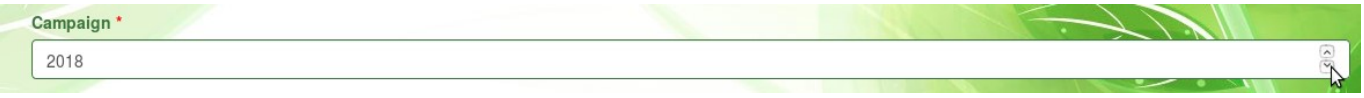

`Place`. Locality or town name used internally to situate the installation location. This field will later be removed in the upcoming developments of PHIS.

`Scientific supervisors`. Email adresses of the experiment supervisor(s). The email adresses refer uniquely to persons existing on PHIS. If unavailable in the predefined list, emails can be added to PHIS from the `Persons` menu, prior to the new project creation. Please see the **Persons** section of this documentation for further details.

`Technical supervisors`. Email adresses of the technicians and scientists (including phd students, interns, etc.) involved in the experiment implementation. The email adresses refer uniquely to persons existing on PHIS.

`Crop Species`. Common name of the crop species which are the object of the experiment. Each crop species names must be separated by a comm. Preferably provided in English, without any capital letters. A link to the internal ontology is under development.

`Groups`. List of the PHIS users groups authorized to access the new experiment. This field is crucial when creating an experiment. Only a PHIS user belonging to at least one of the groups specified in this field will be authorized to access the newly created experiment. An experiment assigned to no group is by default set as public. Please go to **Access rights** for further details on this matter.

`Objective`. A short synthesis of the experiemnt objectives is to be provided in plain text, preferably in English.
A more complete description will be asked in the **Comment** field.

`Keywords`. Keywords characterizing the experiment. Each keyword, should be separated by a comma and not include any capital letter, e.g. *nitrogen use efficiency, rapeseed*.

`Comment`. Complete plain text description of the experiment, preferably provided in English language. Additionally to the description, detailed knowledge on the experimentation can be provided through uploaded files, but only after the experiment has been created.

Completing the experiment creation within PHIS is then achieved by clicking on the `Create` button.

From **Home / Experiments**, one can click on the eye icon on the right to see the newly created experiment information sheet.

The `Add document` button at the top of the experiment sheet remains orange until a first document has been added, e.g. an experimental design. See the **Documents** section below for further information on documents.

Once created, the experiment specifics can be modified with the `Update` button.

Map visualization

Objects selection

scientific objects associated with a given experiment, for example plots, can be displayed on a map, thanks to their geopositioning informations. In PHIS, a map refers to a dynamic map where all the scientific objects of a given experiment are represented.

After having imported scientific objects linked to an experiment, the map associated with this experiment is created within the `Experiments` menu through the `Generate Map` button.

Once the map of an experiment has been created, there's no need to re-create it if no new scientific object has been linked to this experiment. In such cases, the access to the experiment map is realized within the `Experiments` menu through the `Map Visualization` button.

The map of an experiment is dynamic: one can zoom in and out with the **+** and **-** sign at the top left corner of the map, or simply using the mouse wheel. The map visualization also works with a touchscreen. Map rotation is performed pressing `Alt + Shift` while draging the mouse pointer. Selecting multiple contiguous scientific objects is performed pressing `Ctrl + Left Click` and then dragging the mouse pointer (still holding the mouse left click).

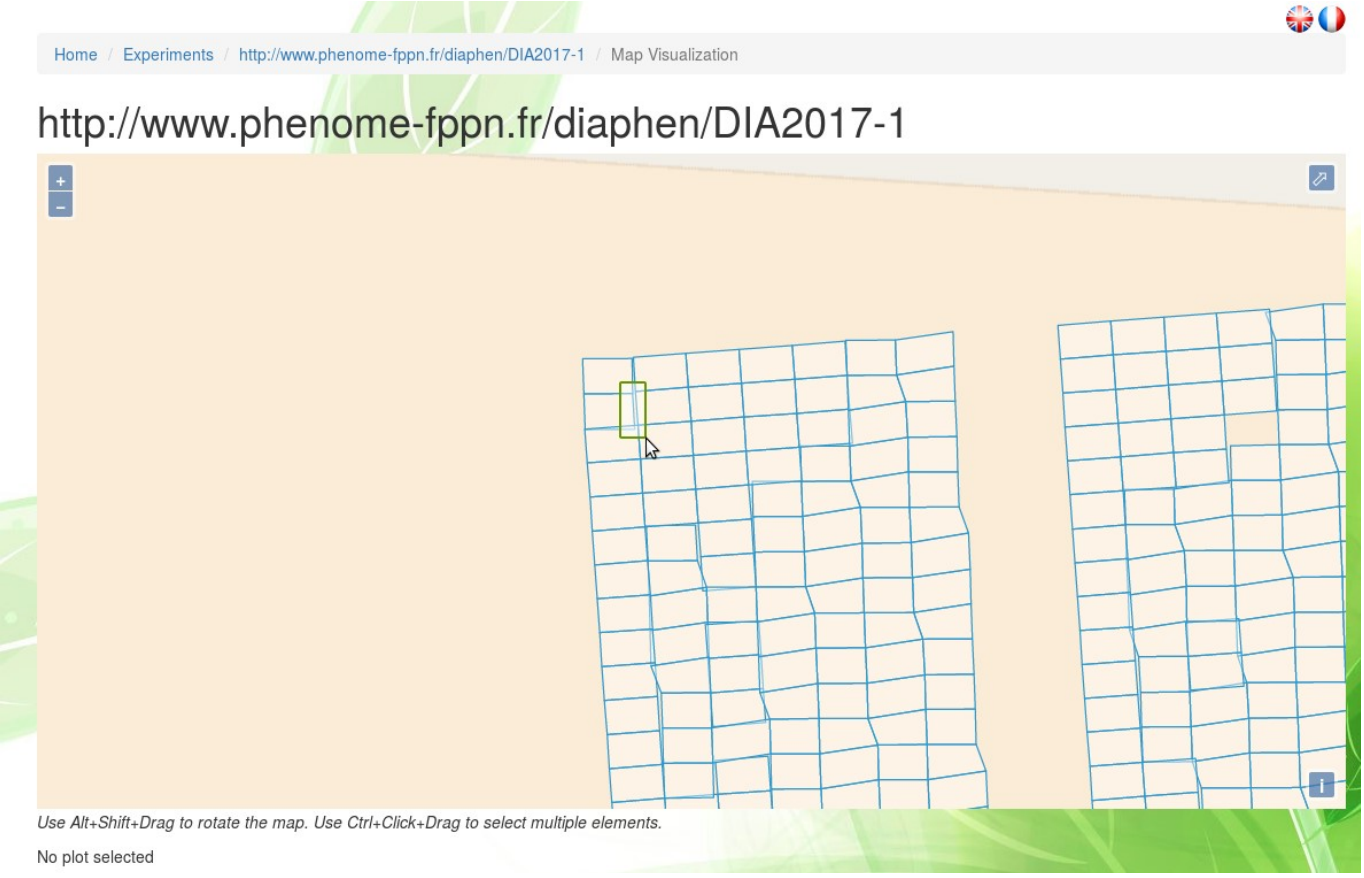

When scientific objects of a map are selected, their attributes (alias within the experiment, crop specied, variety, modality, repetition) are displayed in a table under the map.

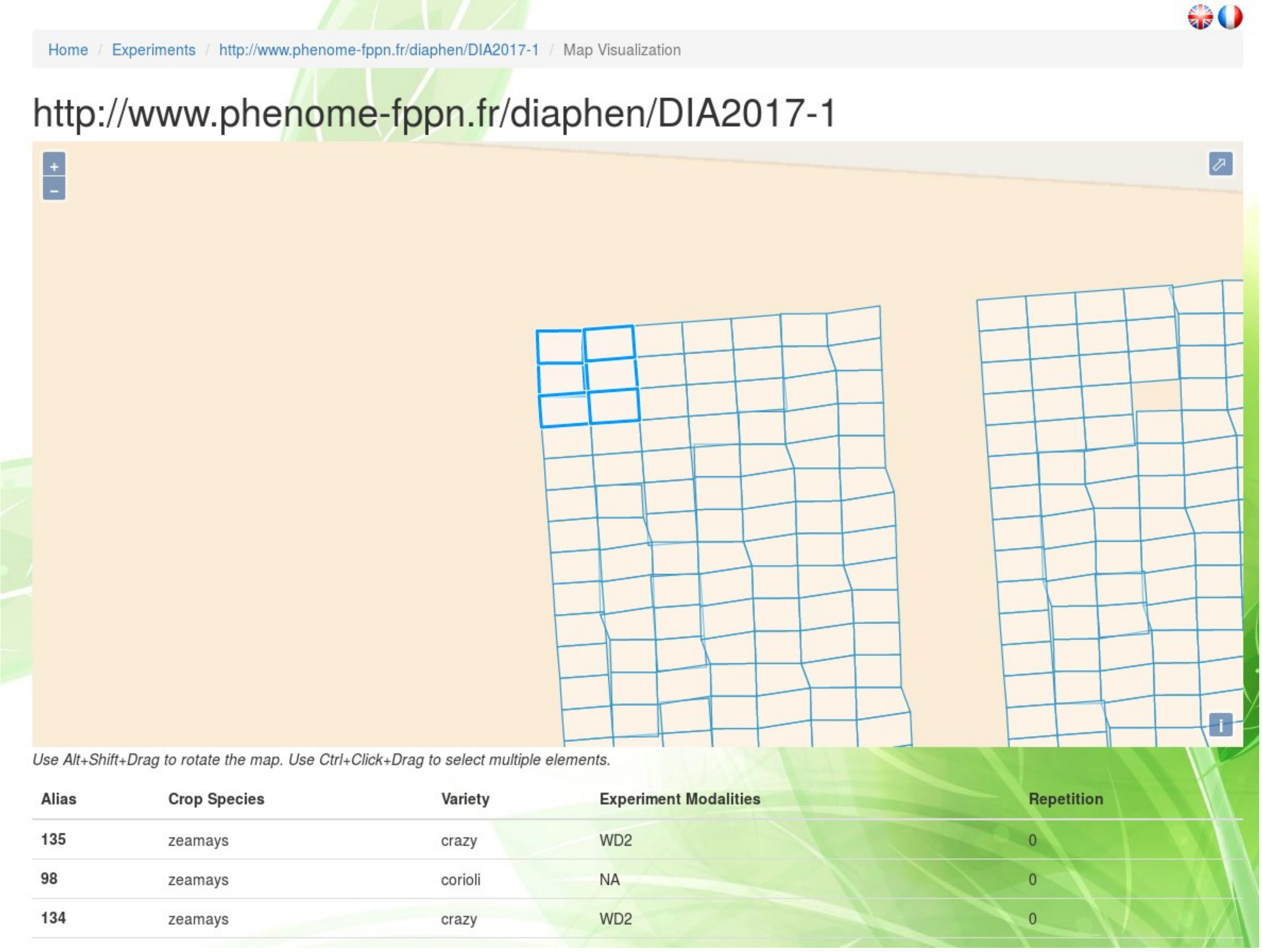

Alias	Crop Species	Variety	Experiment Modalities	Repetition
135	zeamays	crazy	WD2	0
98	zeamays	corioli	NA	0
134	zeamays	crazy	WD2	0

Graphics from datasets

Another feature provided by the `Map Visualization` menu is the possibility to plot graphics from datasets associated to an experiment. A first step is to select on the map scientific objects (e.g. plots) as explained **above**. In order to order to create clear graphics,one should avoid selecting too many objects at the same time. At that point, a new section, **Dataset(s) Visualization (On selected plot(s))**, appears under the map where the objects have been selected. The second step is to select the variable of the dataset from which a graphic should be produced. An optionnal filter for the graphic creation enables the user to use only data from a specific time window to produce the desired graphic.

`Quantitative Variable`. Mandatory field. Here is selected a variable of interest from a predefined list of all the variables defined in a given PHIS instance. Variables can be selected only one at a time. If the selected variable is associated to no dataset linked to the present experiment, then no graphic is plotted. In the next PHIS developments, variables associated a gien project or experimentation will be declared at the level of the project/experimentation.

`Date Start`. Optionnal. First date from which variable values are used to produce a graphic. Variable values associated to a previous date will not appear in the graphic.

`Date End`. Optionnal. Last date at which variable values are used to produce a graphic. Variable values associated to a later date will not appear in the graphic.

The last step is to press the `Search` button, which will lead to the creation of a graphic displaying according to a time axis the variable values associated to :

- the scientific objects selected on the map
- the variable selected in the **Quantitative Variable** field
- potentially the time window between **Date Start** and **Date End** if those fields have been filled out

On the new graphic, the time is displayed on the abscissa axis and the variable on the ordinate axis. Each points of the same color are associated to the same scientific object which is identified below the graphic by its URI. In future PHIS developments, objects alias instead of URIs will be used as curve labels. Variable values and curve associated to an scientific object can be masked by clicking on its associated curve label which at this point changes from black to grey. Clicking a second time on the label as the effect to show again the previously hidden curve.

Documents

What are PHIS documents?

In order to ease the comprehension of projects and experiments, it is possible to upload various documents PHIS and connect them to projects or experiments. The same document can be linked to several projects and experiments. A document can be related to no project nor experiment, but this is not recommanded. Documents that could help persons understand a project are typically (reasearch) contract, phd (or master) thesis, or various multimedia content such as photos or beamer presentations. Similarly, one could expect knowledge about an experimentation to be provided through documents that could be protocols, experimental designs, technical or scientific files, data files, research papers, etc. Examples of document formats are PDF, txt, csv, png images, etc. However, large files are not yet supported by PHIS: a document cannot exceed 2MB.

The documents uploaded to PHIS through the `Documents` section are meant for human consultation. However, metadata (intelligible to machines) must be provided for every new document. These metadata contain the document properties:

- title
- creator
- language
- creation date
- document type

Information of a document can later be changed, while the document itself cannot be modified.

Metadata are specified with **Dublin Core** recommendations. Metadata enables the documents to be stored in the PHIS triplestore. The expression of Dublin Core metadata using the Resource Description Framework is described **Here**.

Upload a document

Within the `Documents` menu, as well as from a project or experimentation information sheet, any PHIS user can upload a new document and specify its metadata with the `Create Document` button. No admin rights are required from a PHIS user to add a new document.

`Title`. Title of the uploaded document. No specific naming convention is required for filling this field. A document title does not have to match the name of the uploaded file it is imported from.

`Creator`. Name(s) of the document creator(s), separated by commas No specific naming convention is required for filling this field.

`Language`. Language in which the document is provided. In accordance to the **Dublin Core Element Set v1.1** document, the value of the language element is defined by **RFC 1766** which includes a two-character language code taken from the **ISO639** standard. The language code should be provided in lower case (e.g. *fr* for French, *en* for English, etc.).

`Creation Date`. Date of the document creation. If unknown, the current date (i.e. the date at the moment of the document upload into PHIS) can be used.

`Concerned Projects`. Project(s) for which the document is relevant. One can select the name of the given project(s) only within the exhaustive list of projects registered to the PHIS instance where the new document is uploaded.

`Concerned Experimentation`. Experiment(s) for which the new document is relevant. A PHIS user can select the name of the given experiment(s) only within the exhaustive list of experiments it has access to.

`Document Type`. The nature of the document's content. A single type has to be selected from a predefined list. Only one doucment type can be selected. PHIS controlled vocabulary of document types is defined in PHIS ontology. If an element seems to be missing from the proposed predefined list, please contact PHIS managers (see *README.md* for PHIS managers contact).

`File`. This field enables PHIS users to upload the new document from their computer through the `Browse` button. Only one document at a time can be created, since every document is identified uniquely through an automatically generated URI. The uploaded file cannot be empty (it has to exceed 0B). For now, a document cannot exceed 2MB, due to technical problems encountered with **Apache** server.

Once the file has been uploaded, do not click on the `Upload` button but `Create` underneath, only when all fields have been completed. The upload button is bound to be abandonned in the following PHIS development.

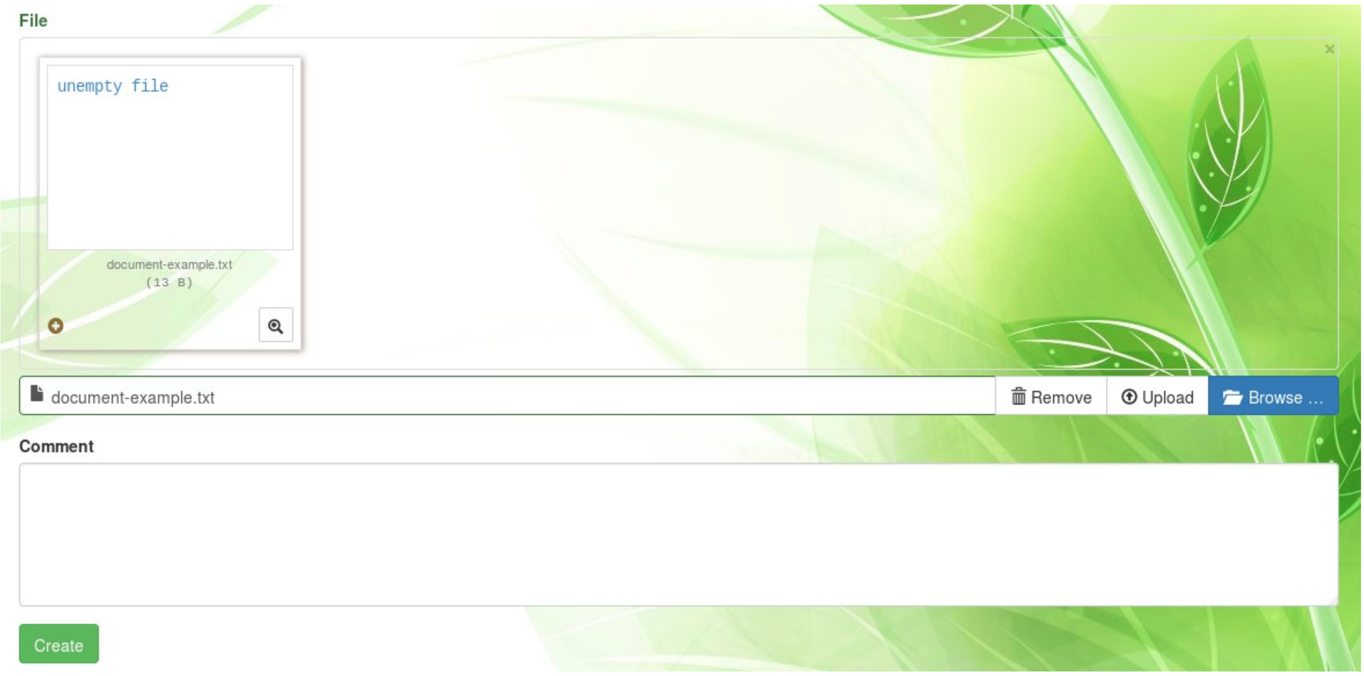

`Comment`. Complete plain text description of the new document, preferably provided in English language.

Click on the `Create` button to complete the document creation, i.e. the document upload and the specification of its metadata with Dublin Core standards.

The list of documents a given user has access to is avaible from the navigation bar through the `Tools > Documents` menu. From **Home / Documents**, one can click on the eye icon on the right to see a given document information sheet (metadata). From there, the document cannot be modified or deleted (in PHIS current version). However, the document can be downloaded with the `Download` button, and its metadata can be modified with the `Update` button.

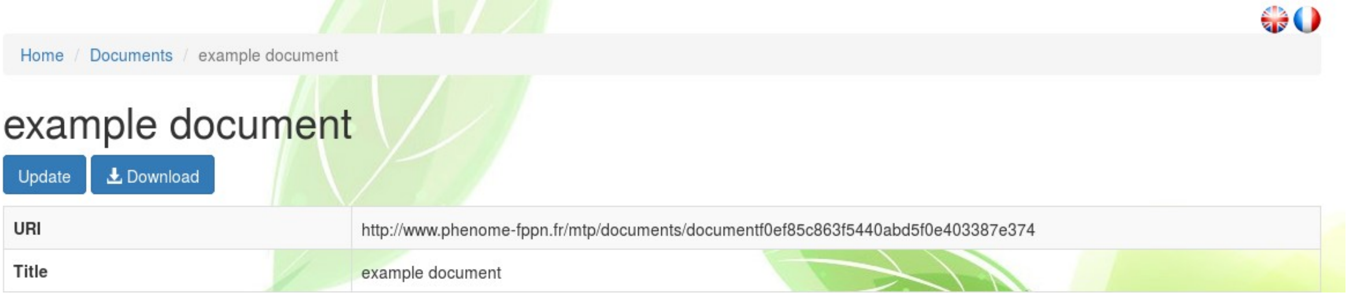

Scientific Objects

Object types

Plant phenotyping experiments all revolve around one entity of interest : plants ! PHIS experiments are focused on a given type of elements : **Scientific objects**, which are no more than declinations of plants, at various scales :

- **plant organ** : part of a given plant
- **plant** : single individual, displaying a single genotype, which is refered to as a **variety plot** :
- smallest spatial unit, a.k.a micro-plot (one treatment maximum can be applied on a plot)
- **block** : combination of plots, generally forming an environmentally homogeneous entity **field**
- : large spatial unit that includes plots and potentially blocks

These scientific objects, forming the basic units of experimentations, are observed through time, and consequently constitute the origin of phenotypic data. A given sceientific object is required to be associated with one experiment, and one only. Phenotypic data created in an experiment, whether is it directly measured, calculated or estimated, is necessarily linked to sceientific objects.

Every scieentific object is uniquely identified through a standardized URI. Metadata is associate to objects under the form of attributes : alias, experiment modality, etc. The data associated with these objects correspond with the values of phenotypic variables associated

The complete list of scientific objects is available in the `scientific Object Tracking` menu, accessible from PHIS top navigation bar.

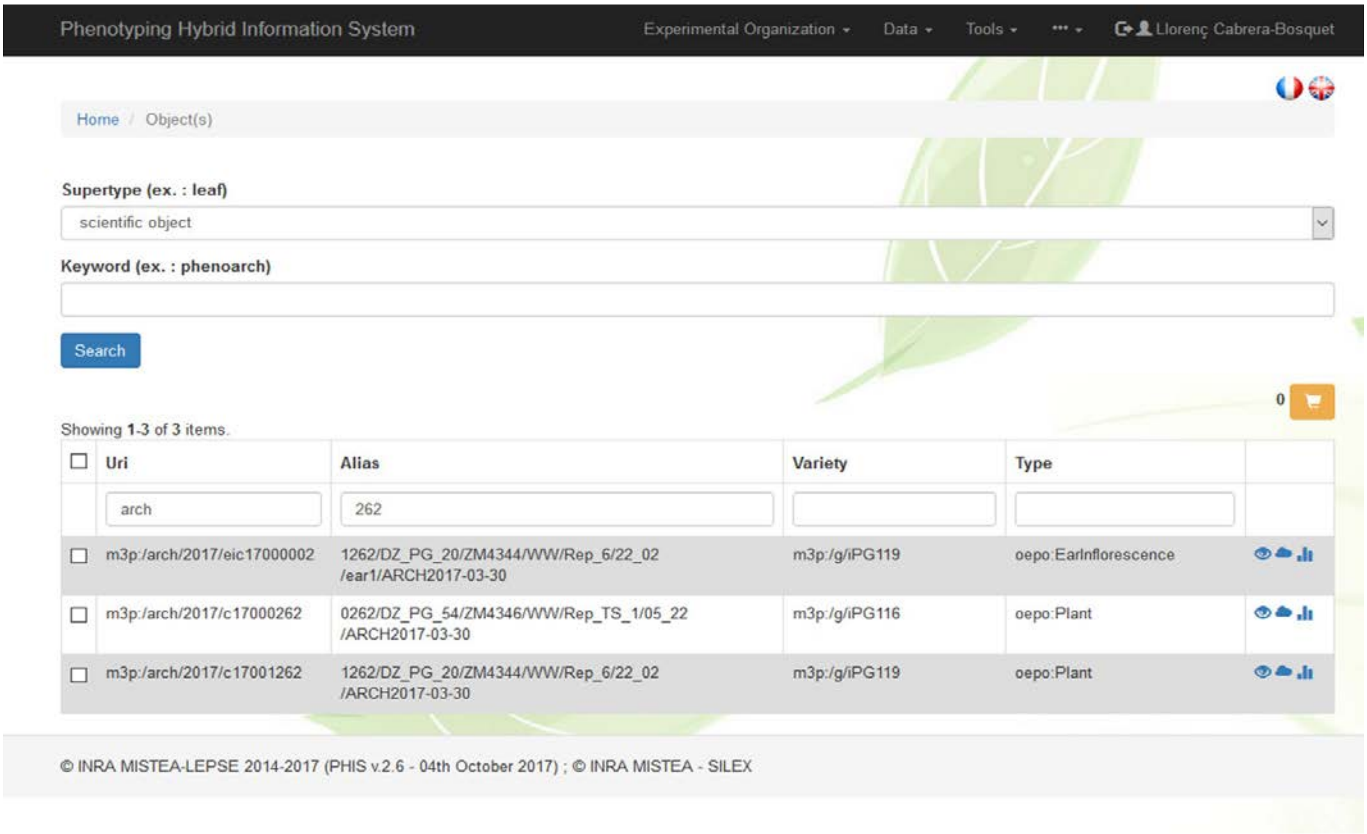

Scientific objects information can also be accessed through the `Experiments` menu. After having selected an experiment, the scientific objects linked to field experiments can be displayed on a map.Moreover, selecting objects on such a map provides additional information on the attributes of these objects. See the **Map Visualization** section for more information on that matter.

Variables

Variables properties

PHIS variables characterize **scientific objects** or their **environment**. Variables characterizing scientific objects are **phenotypic variables**, while variables characterizing the environment in which those scientific objects are studied are refered to as **environmental variables**. PHIS variables can be either directly mesured by a sensor, either computed from one or several variables.

Every variable produced by an experiment must have been previously created in PHIS, and every variable created in PHIS has to be defined unambuigusously. Consequently, when a user refers to a variable in an experiment, there is no ambiguity about the concept he is refering to. Moreover, the usage of unequivocal variables in experiments is a necessary step towards a more intelligible, reliable and reproducible Science.

PHIS variables are listed in the `Variables` menu.

Variable definition is based on the **Crop Ontology guidelines**. Therefore, PHIS variables are all unequivocally characterized by the following triplet:

- a single **trait**, either a phenotypic trait or an environmental feature, which is the subject of the new variable a single **method** of measurement or computation of the trait a single **unit** in which is
- expressed the value of the trait
-

Further information on a given variable is available on its information sheet, accessed to from the variables list through the eye-icon on the right-hand side of this project row.

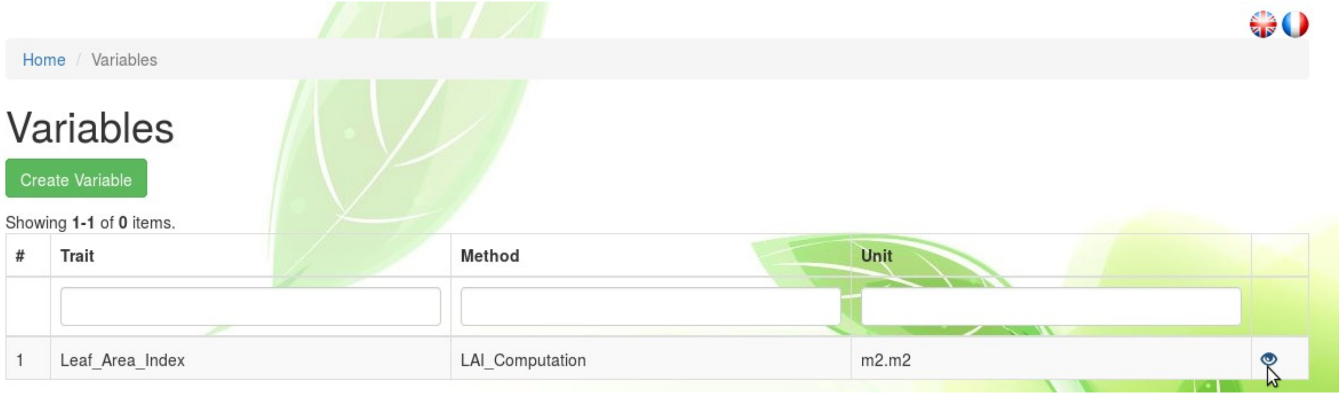

A variable information sheet provides knowledge on this variable, but also on the three elements that define it, namely the **trait**, **method** and **unit** related to this variable. The variable and those three defining features all display a **label**, that should be meaningful and unique, but does not have to be so, an **URI** which is however unique, a **Definition** (or **Comment**) meant for human comprehension, and **related references** meant for Semantic Web applications. Reference to external ontologies is achieved through **SKOS** standards

Create variables, traits, methods and units

Every variable found on PHIS has been previously created by a PHIS user. Within the `Variables` menu, one can create a new project with the `Create Variable` button.

`Variable label`. This field is automatically produced with the concatenation of the trait, method and unit labels, separated by underscores. The resulting variable label is not necessarily unique, but it would be better if it was. On the contrary, the automatically generated URI (not shown in the `Create Variable` menu) is unique.

`Trait`. If the trait associated to the new variable has already been created in PHIS, one can select it through the predefined list of the **Trait label** field. Otherwise, it has to be created, which can be achieved by clicking on the + green icon on the right-hand side of the **Trait label** field.

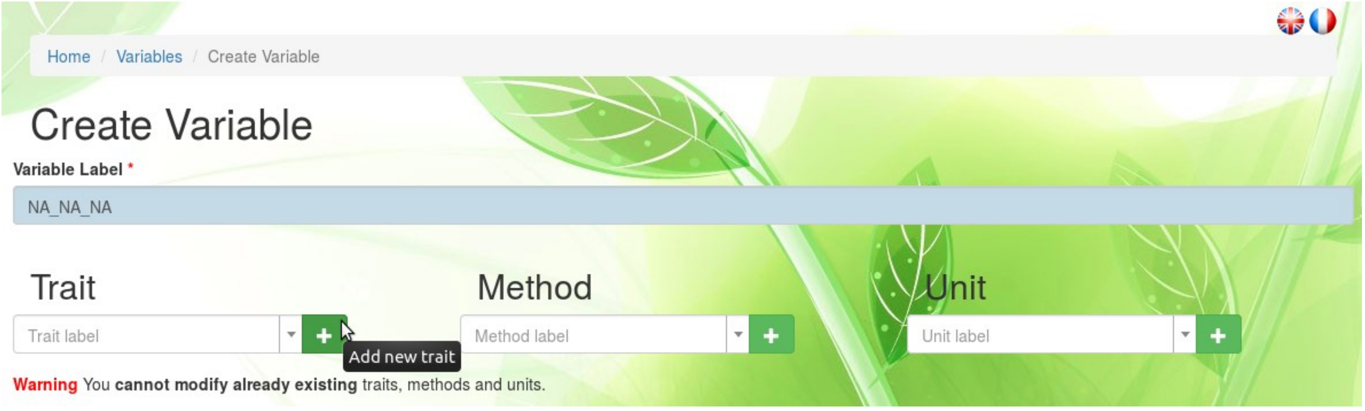

In the case of a new trait, do not fill the `Trait label` field but the `Internal label` one, below the - red icon that replaced the + green icon. This new trait label should be if possible meaningful, distinct from other trait labels, and underscores "_" should be avoided since trait, method and unit labels are concatenated to generate the new variable name with the format *Trait_Method_Unit*.

A `Comment` should be added, preferably in English, in order to explicit the trait specifics as clearly as possible.

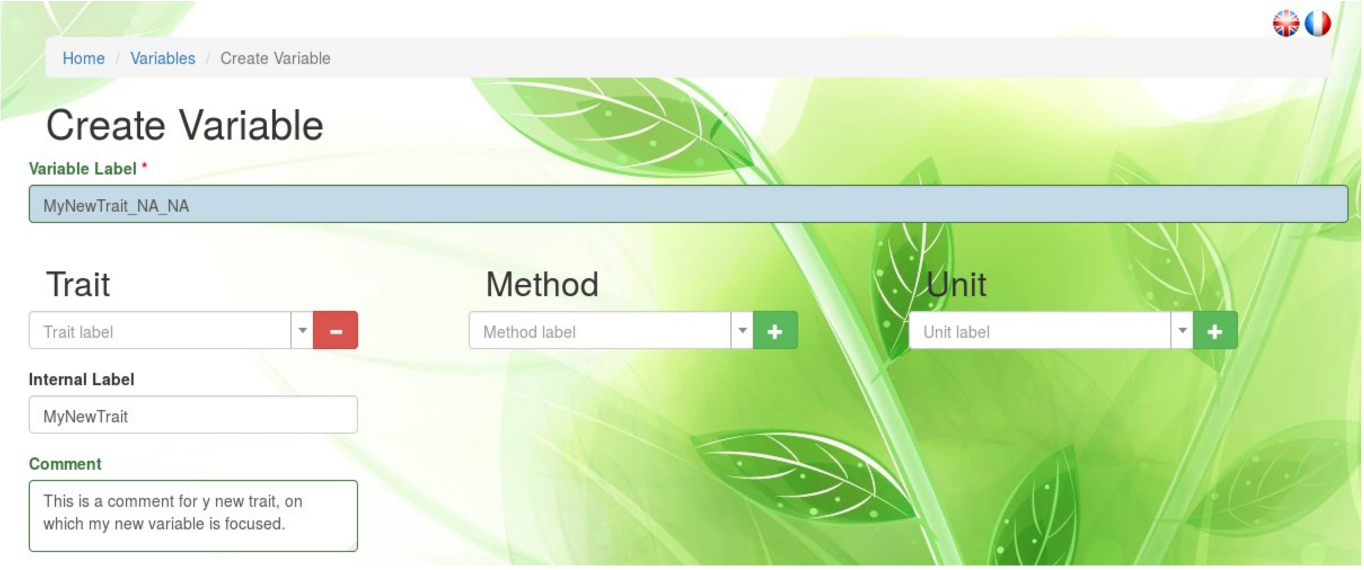

Method. If the method associated to the new variable has already been created in PHIS, one can select it through the predefined list of the **Method label** field. Otherwise, it has to be created, which can be achieved by clicking on the + green icon on the right-hand side of the **Method label** field.

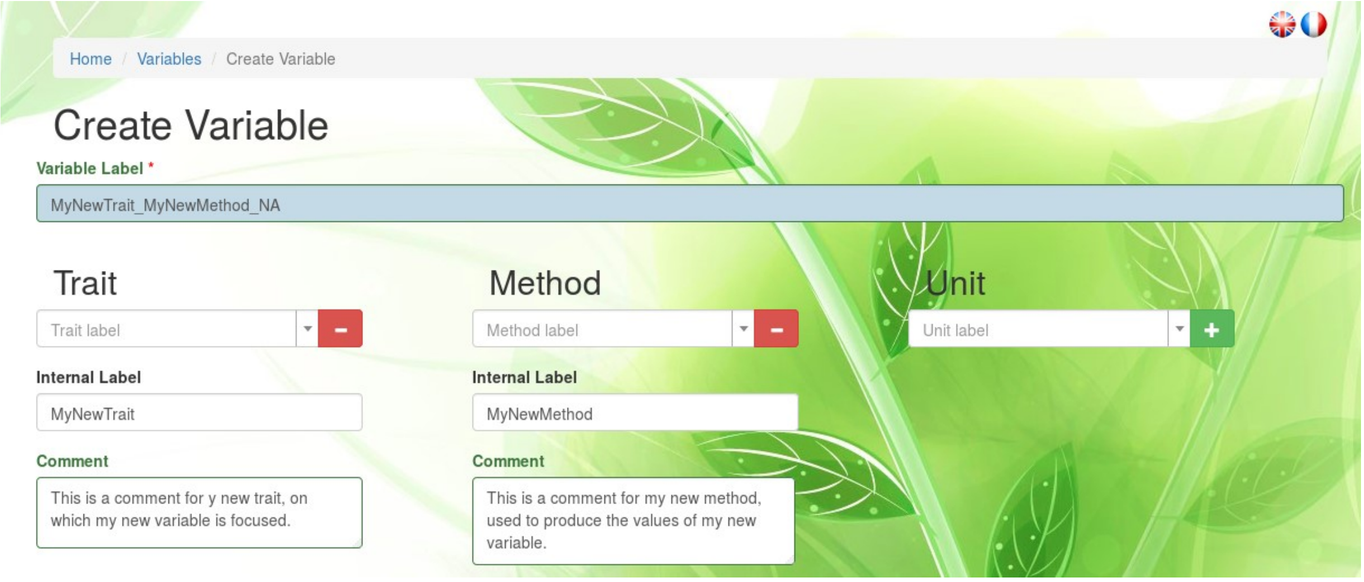

In the case of a new method, do not fill the Method label field but the Internal label one, below the red icon that replaced the + green icon. This new method label should be if possible meaningful, distinct from other method labels, and underscores "_" should be avoided.

A Comment should be added, preferably in English, in order to explicit the method specifics as clearly as possible.

Unit. If the unit associated to the new variable cannot be found on the Unit label predefined list, a new unit has to be created. This can be achieved by clicking on the + green icon on the right-hand side of the **Unit label** field. In the case of a new unit, do not fill the Unit label field but the Internal label one, below the - red icon that replaced the + green icon. This new unit label should be if possible meaningful, distinct from other unit labels, and underscores "_" should be avoided.

Otherwise, if the appropriate unit is already register in PHIS, then one only has to select it from the `Unit label` field, without clicking on the + green icon (or clicking on the - red icon if the + green icon had previously been clicked on).

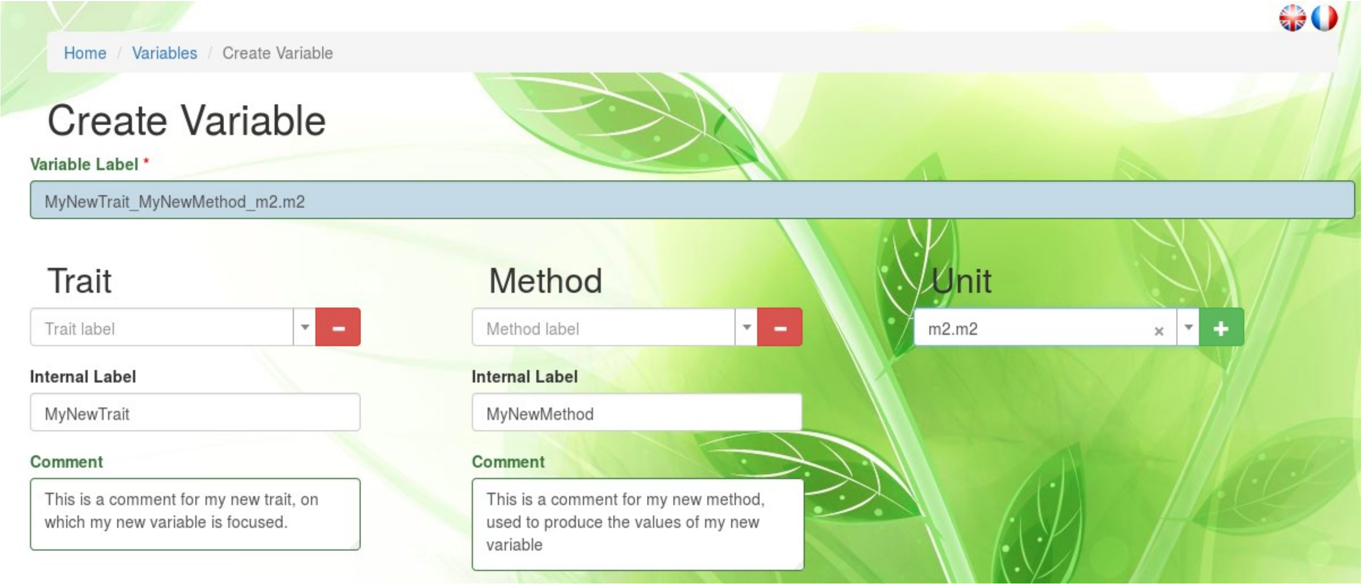

`Related References`. In order to unambiguously define the new variable, semantic relations are established by the user between the new variable and concepts already defined in reliable ontologies existing on the world wide web. These relations to external ontologies are established using **SKOS** (Simple Knowledge Organization System). `Entity` refers to the PHIS entity that will be associated to a concept found in an ontology. This **Entity** be either the new variable, trait, method or unit. It is not required to specify related concepts for traits, methods and units, however it is recommanded. `Relation` refers to the nature of the relation between the entity and the concept defined in an ontology. This semantic relation is provided using SKOS. The entity can either be, compared to an ontology concept, an **exact match**, a **close match**, **narrower** or **broader**.

SKOS mapping properties, `skos:closeMatch` and `skos:exactMatch`, are used to state alignement links between SKOS concepts, as indicated in the w3 SKOS Mapping properties web page :

- **exactMatch** : used to link two concepts, indicating a high degree of confidence that the concepts can be used interchangeably across a wide range of information retrieval applications. `skos:exactMatch` is a transitive property, and is a sub-property of `skos:closeMatch`. *Example:* *`<MyNewNDVIVariable> skos:exactMatch <CO_322:0000880>`* *asserts that the variable 'MyNewNDVIVariable' created in PHIS refers to the exact same concept as does the variable 'NDVI_M_idx' already defined in the Crop Ontology and uniquely identified as 'CO_322:0000880'* • **closeMatch** : used to link two concepts that are sufficiently similar that they can be used interchangeably in some information retrieval applications. In order to avoid the possibility of "compound errors" when combining mappings across more than two concept schemes, `skos:closeMatch` is not declared to be a transitive property. *Example:* *`<MyNewLAIVariable>`*

  `skos:closeMatch <http://www.cropontology.org/rdf/CO_321:0001254>` *asserts that the variable 'MyNewLAIVariable' created in PHIS is very similar to the variable 'LAI_Calc_Idx' already defined in the Crop Ontology and uniquely identified as 'CO_321:0001254'*

SKOS hierarchical properties `skos:broader` and `skos:narrower` are used to assert a direct hierarchical link between two SKOS concepts, as indicated in the w3 Semantic Relations web page :

- **broader** (label=*has broader*) : a triple `<A> skos:broader <B>` asserts that `<B>`, the object of the triple, is a broader concept than `<A>`, the subject of the triple. *Example:* `<MyNewPlantHeightTrait> skos:broader <CO_322:0000994>` *asserts that the trait 'MyNewPlantHeightTrait' created in PHIS refers to a concept that has a broader one: which is the concept refered to by the trait 'Plant height' already defined in the Crop Ontology and uniquely identified as 'CO_322:0000994'*
- **narrower** (label=*has narrower*) : a triple `<C> skos:narrower <D>` asserts that `<D>`, the object of the triple, is a narrower concept than `<C>`, the subject of the triple. `skos:broader` is `owl:inverseOf` the property `skos:narrower`. *Example:* `<MyNewStageEstimationMethod> skos:narrower <http://www.cropontology.org/terms/CO_322:0000905/>` *asserts that the method 'MyNewStageEstimationMethod' created in PHIS refers to a concept that has a narrower one, which is the concept refered to by the method 'Silking date - Estimation' already defined in the Crop Ontology and uniquely identified as 'http://www.cropontology.org/terms/CO_322:0000905/'*

By convention, `skos:broader` and `skos:narrower` are only used to assert a direct (i.e., immediate) hierarchical link between two SKOS concepts. This provides applications with a convenient and reliable way to access the direct broader and narrower links for any given concept. Note that, to support this usage convention, the properties `skos:broader` and `skos:narrower` are not declared as transitive properties.

`Reference URI` refers to the URI of the concept found on ontologies such as the ones suggested in the short list above the **Related References** field. The URI provided here is not necessarily the URL of the web page of the ontology on which the targeted concept is defined. Indeed, the URI of a given concept does not necessarily match with the URL of the web page where this concept has been found. `Hyperlink` (optionnal) refers to the URL where are located the related concept whose URI has been provided in the previous field.

When a variable is created, multiple references using SKOS can be stated, using the **+** white icon.

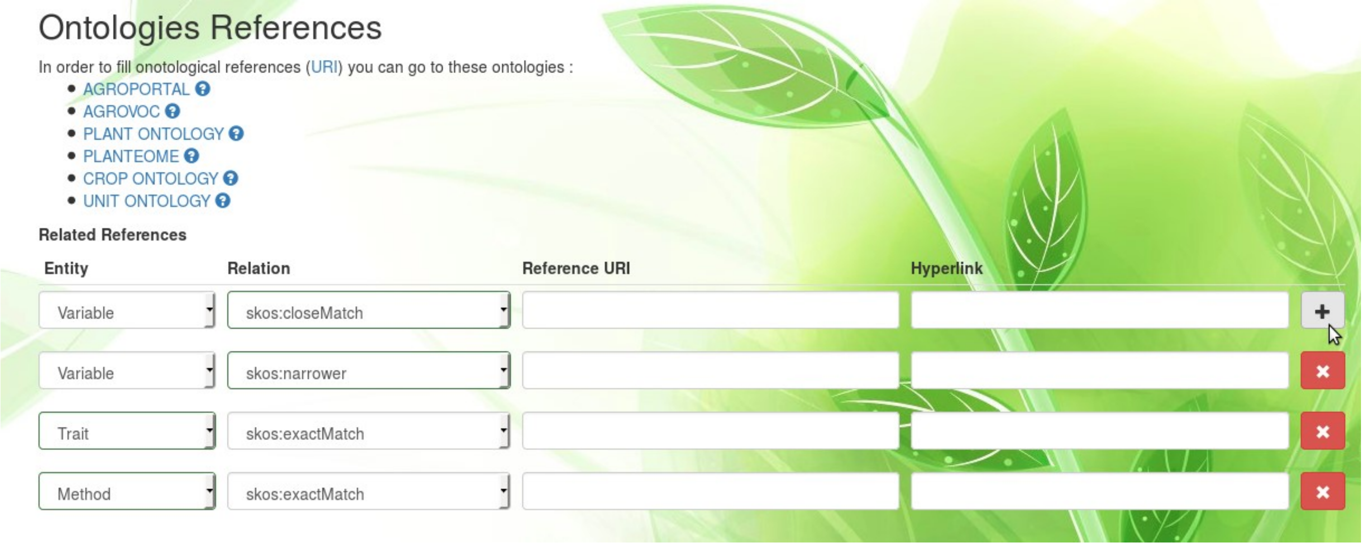

The main ontologies differ on the following features:

The **AgroPortal** project aims to offer a reference ontology repository for agronomy, reusing the NCBO BioPortal technology, as stated on the **FAO website**. The scientific outcomes and the experience of the biomdical domain are thus exploited and transposed into the agronomy domain, including plants, food, environment and possibly animal sciences.

AGROVOC is a controlled vocabulary covering all areas of interest of the United Nations Food and Agriculture Organization (FAO), including food, nutrition, agriculture, fisheries, forestry, environment, etc. It is published by the FAO and edited by a community of experts. More information is provided on the **FAO website**.

The **Plant Ontology (PO)** is a structured vocabulary and database resource that links plant anatomy, morphology, growth and development to plant genomics data. The URL provided **here** redirects visitors to the Planteome website since PO is developed by the Planteome Project, alongside with other reference ontologies listed in the **Planteome Ontologies webpage**.

The **Planteome** project is a centralized platform where reference ontologies for plants will be used to access plant genomics data.

The **Crop Ontology (CO)** current objective is to compile validated concepts along with their interrelationships on anatomy, structure and phenotype of crops, on trait mesurement and methods, as well as on germplasm with the multi-crop passport terms.

The **Unit Ontology** gathers metrical units for use in conjunction with **PATO** (Phenotype And Trait Ontology), which is a phenotypic quality ontology.

`Variable Definition`. Text defining clearly the concept underlying the new variable. This definition should ideally be provided in English.

Devices

The Devices menu contains information about the different devices (environmental sensors, cameras and actuators) declared in the information system.

Different filters including `URI`, `Device Alias`, `Device Type`, `Position` and `Variable` allow to refine search.

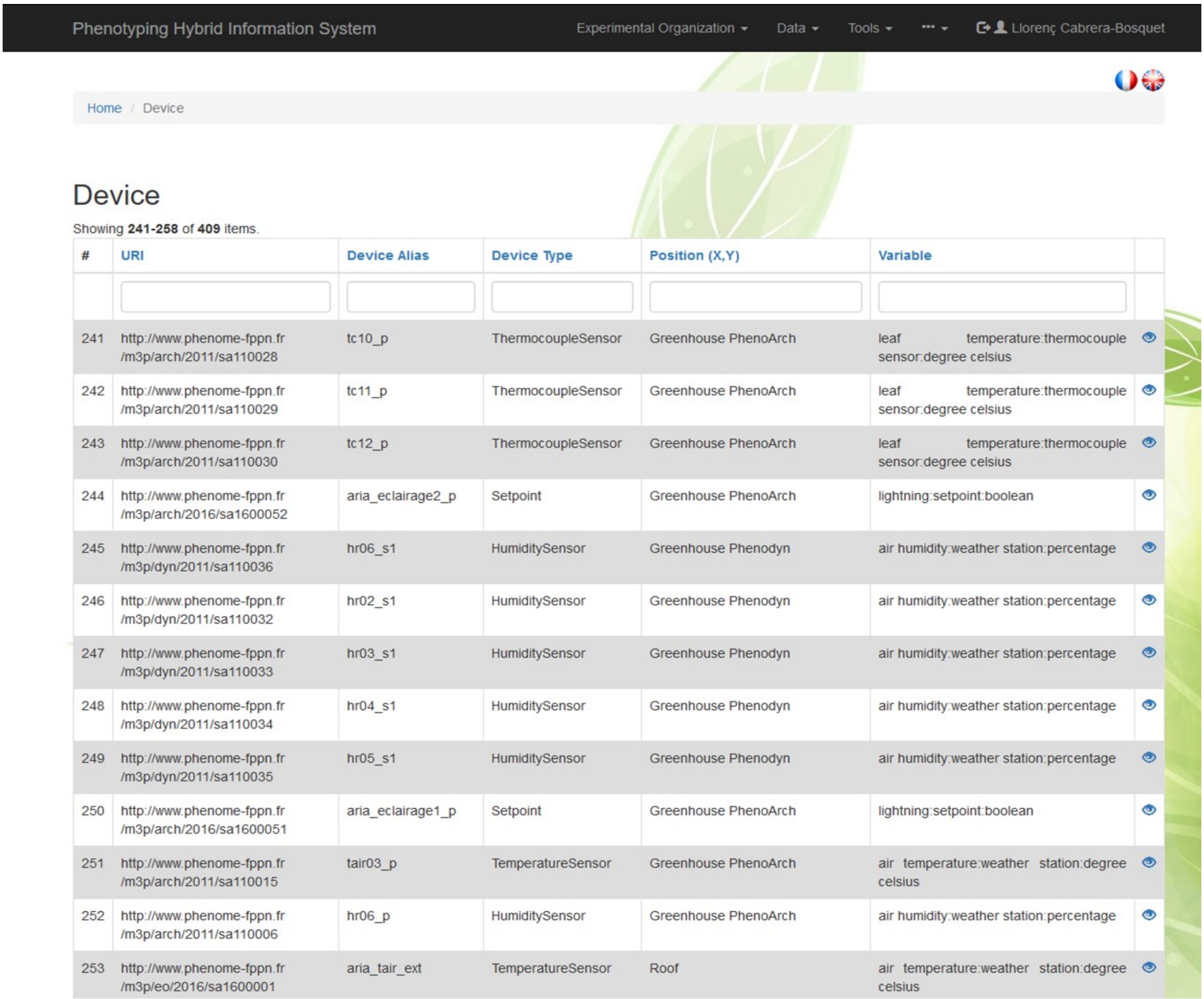

Device

Showing **241-258** of **409** items.

#	URI	Device Alias	Device Type	Position (X,Y)	Variable
241	http://www.phenome-fppn.fr/m3p/arch/2011/sa110028	tc10_p	ThermocoupleSensor	Greenhouse PhenoArch	leaf temperature:thermocouple sensor:degree celsius
242	http://www.phenome-fppn.fr/m3p/arch/2011/sa110029	tc11_p	ThermocoupleSensor	Greenhouse PhenoArch	leaf temperature:thermocouple sensor:degree celsius
243	http://www.phenome-fppn.fr/m3p/arch/2011/sa110030	tc12_p	ThermocoupleSensor	Greenhouse PhenoArch	leaf temperature:thermocouple sensor:degree celsius
244	http://www.phenome-fppn.fr/m3p/arch/2016/sa1600052	aria_eclairage2_p	Setpoint	Greenhouse PhenoArch	lightning:setpoint:boolean
245	http://www.phenome-fppn.fr/m3p/dyn/2011/sa110036	hr06_s1	HumiditySensor	Greenhouse Phenodyn	air humidity:weather station:percentage
246	http://www.phenome-fppn.fr/m3p/dyn/2011/sa110032	hr02_s1	HumiditySensor	Greenhouse Phenodyn	air humidity:weather station:percentage
247	http://www.phenome-fppn.fr/m3p/dyn/2011/sa110033	hr03_s1	HumiditySensor	Greenhouse Phenodyn	air humidity:weather station:percentage
248	http://www.phenome-fppn.fr/m3p/dyn/2011/sa110034	hr04_s1	HumiditySensor	Greenhouse Phenodyn	air humidity:weather station:percentage
249	http://www.phenome-fppn.fr/m3p/dyn/2011/sa110035	hr05_s1	HumiditySensor	Greenhouse Phenodyn	air humidity:weather station:percentage
250	http://www.phenome-fppn.fr/m3p/arch/2016/sa1600051	aria_eclairage1_p	Setpoint	Greenhouse PhenoArch	lightning:setpoint:boolean
251	http://www.phenome-fppn.fr/m3p/arch/2011/sa110015	tair03_p	TemperatureSensor	Greenhouse PhenoArch	air temperature:weather station:degree celsius
252	http://www.phenome-fppn.fr/m3p/arch/2011/sa110006	hr06_p	HumiditySensor	Greenhouse PhenoArch	air humidity:weather station:percentage
253	http://www.phenome-fppn.fr/m3p/eo/2016/sa1600001	aria_tair_ext	TemperatureSensor	Roof	air temperature:weather station:degree celsius

Detailed information of each device including the brand, model, together with technical documentation and events associated to each device (e.g. calibration, dysfunction) can be accessed by clicking on the eye icon placed on the right. Outputs of sensors can also be visualised.

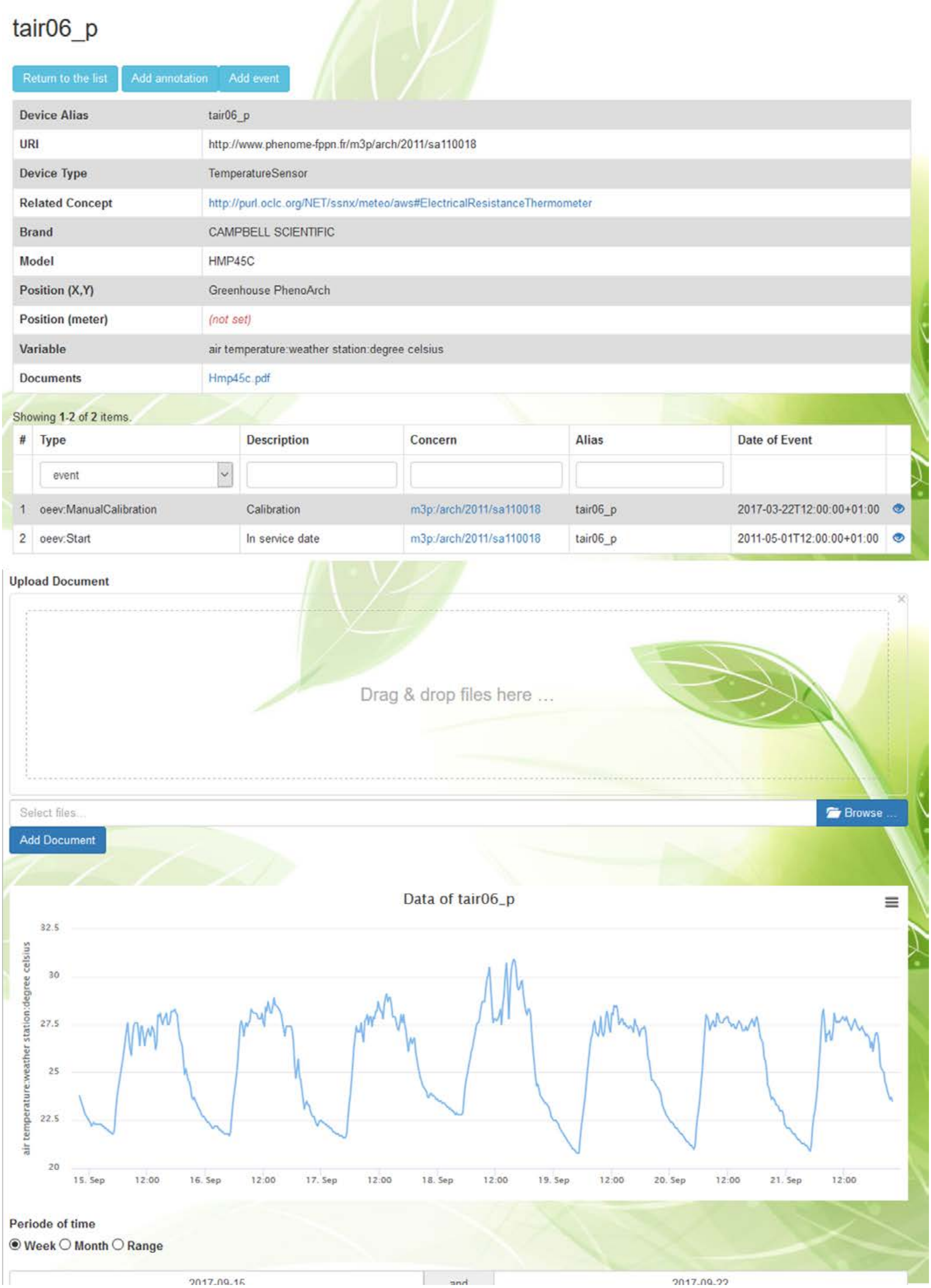

RDF triples can be explored including the events and environmental variables associated to each sensor.

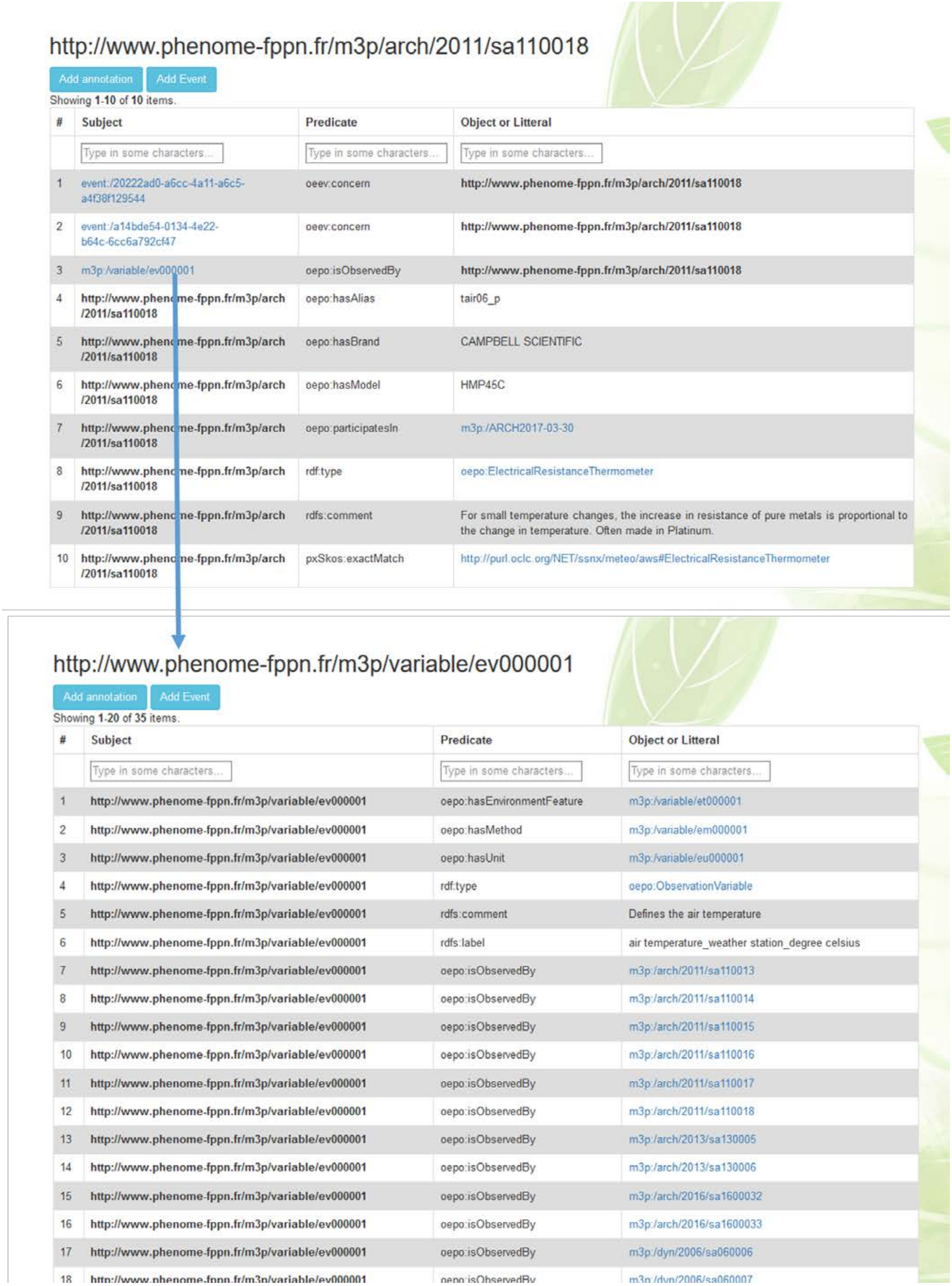

http://www.phenome-fppn.fr/m3p/arch/2011/sa110018

Add annotation Add Event

Showing 1-10 of 10 items.

#	Subject	Predicate	Object or Litteral
	Type in some characters...	Type in some characters...	Type in some characters...
1	event:/20222ad0-a6cc-4a11-a6c5-a4f38f129544	oeev:concern	http://www.phenome-fppn.fr/m3p/arch/2011/sa110018
2	event:/a14bde54-0134-4e22-b64c-6cc6a792cf47	oeev:concern	http://www.phenome-fppn.fr/m3p/arch/2011/sa110018
3	m3p:/variable/ev000001	oepo:isObservedBy	http://www.phenome-fppn.fr/m3p/arch/2011/sa110018
4	http://www.phenome-fppn.fr/m3p/arch/2011/sa110018	oepo:hasAlias	tair06_p
5	http://www.phenome-fppn.fr/m3p/arch/2011/sa110018	oepo:hasBrand	CAMPBELL SCIENTIFIC
6	http://www.phenome-fppn.fr/m3p/arch/2011/sa110018	oepo:hasModel	HMP45C
7	http://www.phenome-fppn.fr/m3p/arch/2011/sa110018	oepo:participatesIn	m3p:/ARCH2017-03-30
8	http://www.phenome-fppn.fr/m3p/arch/2011/sa110018	rdf:type	oepo:ElectricalResistanceThermometer
9	http://www.phenome-fppn.fr/m3p/arch/2011/sa110018	rdfs:comment	For small temperature changes, the increase in resistance of pure metals is proportional to the change in temperature. Often made in Platinum.
10	http://www.phenome-fppn.fr/m3p/arch/2011/sa110018	pxSkos:exactMatch	http://purl.oclc.org/NET/ssnx/meteo/aws#ElectricalResistanceThermometer

http://www.phenome-fppn.fr/m3p/variable/ev000001

Add annotation Add Event

Showing 1-20 of 35 items.

#	Subject	Predicate	Object or Litteral
	Type in some characters...	Type in some characters...	Type in some characters...
1	http://www.phenome-fppn.fr/m3p/variable/ev000001	oepo:hasEnvironmentFeature	m3p:/variable/et000001
2	http://www.phenome-fppn.fr/m3p/variable/ev000001	oepo:hasMethod	m3p:/variable/em000001
3	http://www.phenome-fppn.fr/m3p/variable/ev000001	oepo:hasUnit	m3p:/variable/eu000001
4	http://www.phenome-fppn.fr/m3p/variable/ev000001	rdf:type	oepo:ObservationVariable
5	http://www.phenome-fppn.fr/m3p/variable/ev000001	rdfs:comment	Defines the air temperature
6	http://www.phenome-fppn.fr/m3p/variable/ev000001	rdfs:label	air temperature_weather station_degree celsius
7	http://www.phenome-fppn.fr/m3p/variable/ev000001	oepo:isObservedBy	m3p:/arch/2011/sa110013
8	http://www.phenome-fppn.fr/m3p/variable/ev000001	oepo:isObservedBy	m3p:/arch/2011/sa110014
9	http://www.phenome-fppn.fr/m3p/variable/ev000001	oepo:isObservedBy	m3p:/arch/2011/sa110015
10	http://www.phenome-fppn.fr/m3p/variable/ev000001	oepo:isObservedBy	m3p:/arch/2011/sa110016
11	http://www.phenome-fppn.fr/m3p/variable/ev000001	oepo:isObservedBy	m3p:/arch/2011/sa110017
12	http://www.phenome-fppn.fr/m3p/variable/ev000001	oepo:isObservedBy	m3p:/arch/2011/sa110018
13	http://www.phenome-fppn.fr/m3p/variable/ev000001	oepo:isObservedBy	m3p:/arch/2013/sa130005
14	http://www.phenome-fppn.fr/m3p/variable/ev000001	oepo:isObservedBy	m3p:/arch/2013/sa130006
15	http://www.phenome-fppn.fr/m3p/variable/ev000001	oepo:isObservedBy	m3p:/arch/2016/sa1600032
16	http://www.phenome-fppn.fr/m3p/variable/ev000001	oepo:isObservedBy	m3p:/arch/2016/sa1600033
17	http://www.phenome-fppn.fr/m3p/variable/ev000001	oepo:isObservedBy	m3p:/dyn/2006/sa060006
18	http://www.phenome-fppn.fr/m3p/variable/ev000001	oepo:isObservedBy	m3p:/dyn/2006/sa060007

Germplasm

Germplasm menu allows searching for the different genetic material declared in PHIS. Different filters based on mathematical oprators allow refining the search by applying different constraints.

For instance, the query of maize genotypes displaying `leaf area > 0.8 m^2` gives two results:

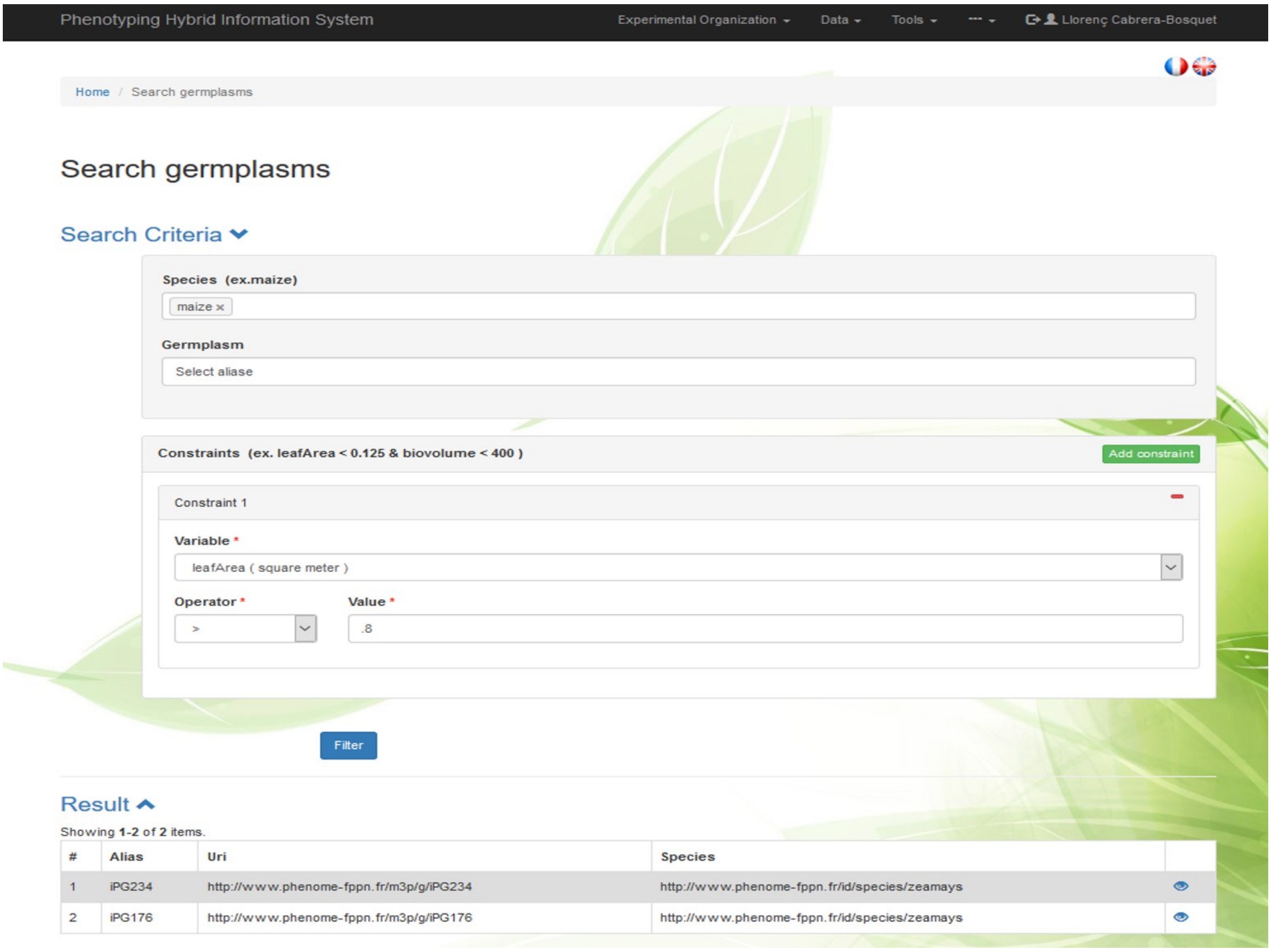

Further, by clicking on one of the results (e.g. genotype *iPG234*) the user can visualise those plants corresponding to this gentotype,and those that validate de search criteria (i.e. *leaf area > 0.8* m^2) are highlited in green:

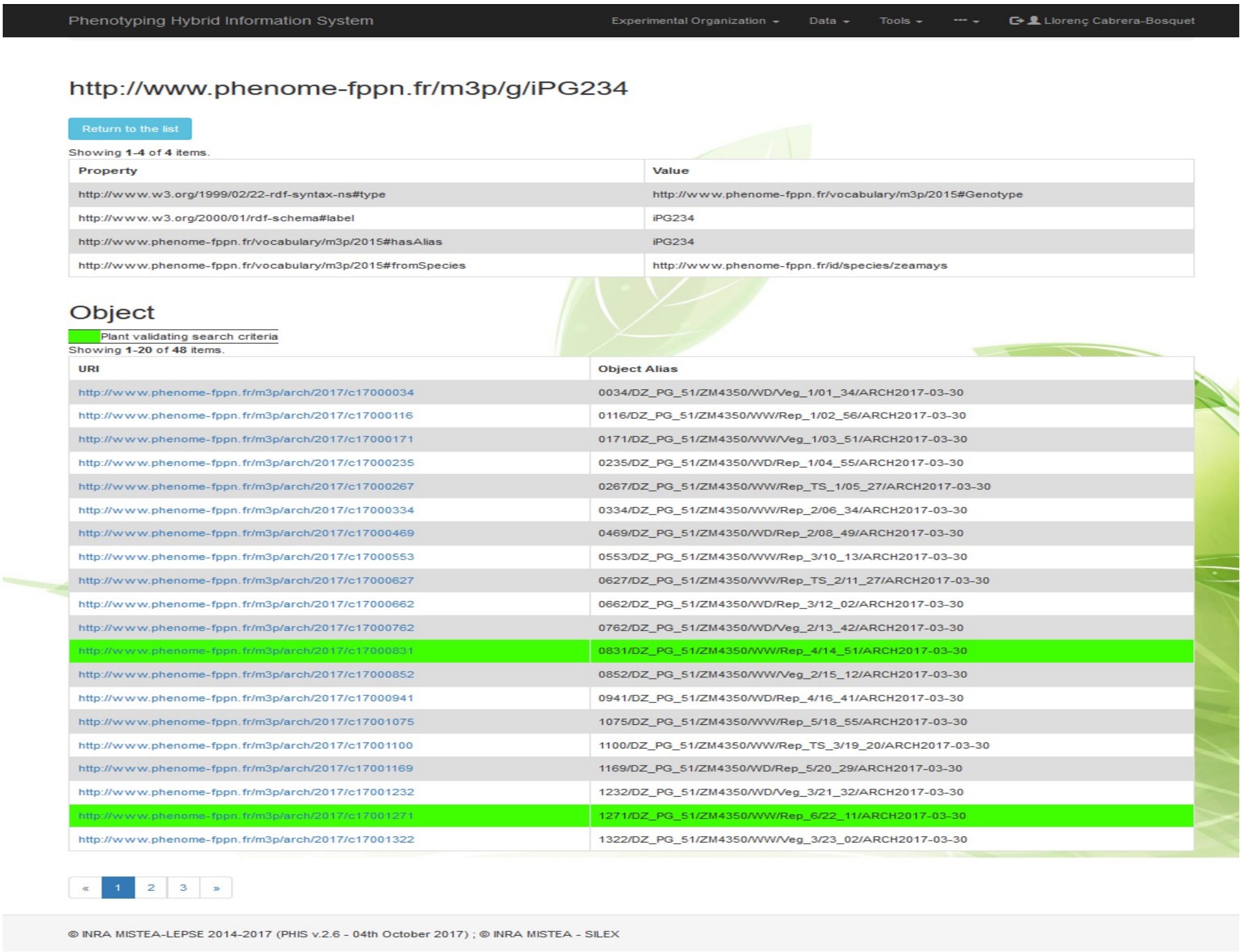

Phenotyping Hybrid Information System   Experimental Organization   Data   Tools   ---   Llorenç Cabrera-Bosquet

http://www.phenome-fppn.fr/m3p/g/iPG234

Return to the list

Showing 1-4 of 4 items.

Property	Value
http://www.w3.org/1999/02/22-rdf-syntax-ns#type	http://www.phenome-fppn.fr/vocabulary/m3p/2015#Genotype
http://www.w3.org/2000/01/rdf-schema#label	iPG234
http://www.phenome-fppn.fr/vocabulary/m3p/2015#hasAlias	iPG234
http://www.phenome-fppn.fr/vocabulary/m3p/2015#fromSpecies	http://www.phenome-fppn.fr/id/species/zeamays

Object

Plant validating search criteria

Showing 1-20 of 48 items.

URI	Object Alias
http://www.phenome-fppn.fr/m3p/arch/2017/c17000034	0034/DZ_PG_51/ZM4350/WD/Veg_1/01_34/ARCH2017-03-30
http://www.phenome-fppn.fr/m3p/arch/2017/c17000116	0116/DZ_PG_51/ZM4350/WW/Rep_1/02_56/ARCH2017-03-30
http://www.phenome-fppn.fr/m3p/arch/2017/c17000171	0171/DZ_PG_51/ZM4350/WW/Veg_1/03_51/ARCH2017-03-30
http://www.phenome-fppn.fr/m3p/arch/2017/c17000235	0235/DZ_PG_51/ZM4350/WD/Rep_1/04_55/ARCH2017-03-30
http://www.phenome-fppn.fr/m3p/arch/2017/c17000267	0267/DZ_PG_51/ZM4350/WW/Rep_TS_1/05_27/ARCH2017-03-30
http://www.phenome-fppn.fr/m3p/arch/2017/c17000334	0334/DZ_PG_51/ZM4350/WW/Rep_2/06_34/ARCH2017-03-30
http://www.phenome-fppn.fr/m3p/arch/2017/c17000469	0469/DZ_PG_51/ZM4350/WD/Rep_2/08_49/ARCH2017-03-30
http://www.phenome-fppn.fr/m3p/arch/2017/c17000553	0553/DZ_PG_51/ZM4350/WW/Rep_3/10_13/ARCH2017-03-30
http://www.phenome-fppn.fr/m3p/arch/2017/c17000627	0627/DZ_PG_51/ZM4350/WW/Rep_TS_2/11_27/ARCH2017-03-30
http://www.phenome-fppn.fr/m3p/arch/2017/c17000662	0662/DZ_PG_51/ZM4350/WD/Rep_3/12_02/ARCH2017-03-30
http://www.phenome-fppn.fr/m3p/arch/2017/c17000762	0762/DZ_PG_51/ZM4350/WD/Veg_2/13_42/ARCH2017-03-30
http://www.phenome-fppn.fr/m3p/arch/2017/c17000831	0831/DZ_PG_51/ZM4350/WW/Rep_4/14_51/ARCH2017-03-30
http://www.phenome-fppn.fr/m3p/arch/2017/c17000852	0852/DZ_PG_51/ZM4350/WW/Veg_2/15_12/ARCH2017-03-30
http://www.phenome-fppn.fr/m3p/arch/2017/c17000941	0941/DZ_PG_51/ZM4350/WD/Rep_4/16_41/ARCH2017-03-30
http://www.phenome-fppn.fr/m3p/arch/2017/c17001075	1075/DZ_PG_51/ZM4350/WW/Rep_5/18_55/ARCH2017-03-30
http://www.phenome-fppn.fr/m3p/arch/2017/c17001100	1100/DZ_PG_51/ZM4350/WW/Rep_TS_3/19_20/ARCH2017-03-30
http://www.phenome-fppn.fr/m3p/arch/2017/c17001169	1169/DZ_PG_51/ZM4350/WD/Rep_5/20_29/ARCH2017-03-30
http://www.phenome-fppn.fr/m3p/arch/2017/c17001232	1232/DZ_PG_51/ZM4350/WD/Veg_3/21_32/ARCH2017-03-30
http://www.phenome-fppn.fr/m3p/arch/2017/c17001271	1271/DZ_PG_51/ZM4350/WW/Rep_6/22_11/ARCH2017-03-30
http://www.phenome-fppn.fr/m3p/arch/2017/c17001322	1322/DZ_PG_51/ZM4350/WW/Veg_3/23_02/ARCH2017-03-30

« 1 2 3 »

© INRA MISTEA-LEPSE 2014-2017 (PHIS v.2.6 - 04th October 2017) ; © INRA MISTEA - SILEX

Multiple search criteria is supported, for instance, the query search for `genotype **iPG082**` displaying `leaf area > 0.4 m²` and `biovolume > 400g` displays all plants corresponding to this genotype validating these two criteria:

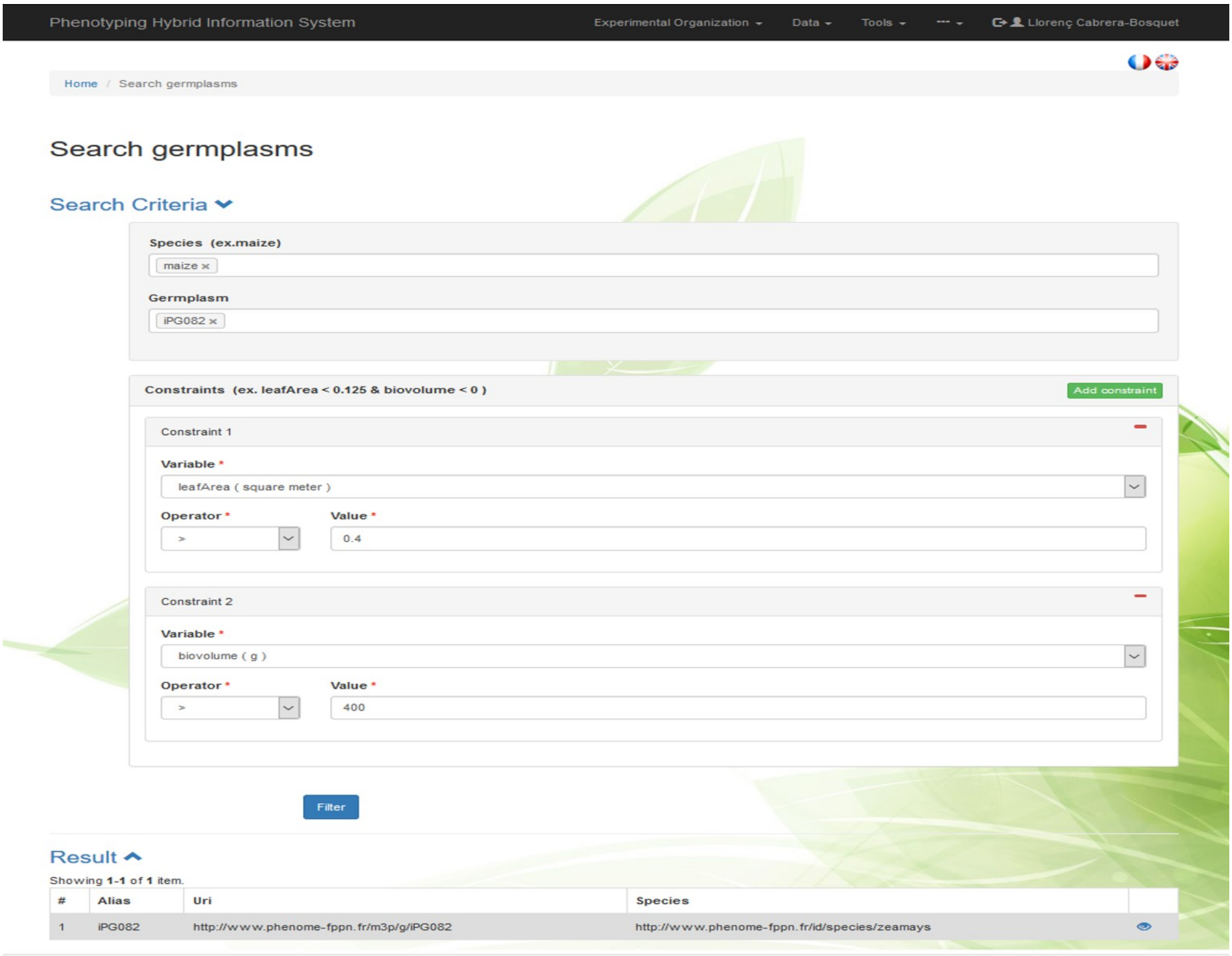
Phenotyping Hybrid Information System
Experimental Organization
Data
Tools
Llorenç Cabrera-Bosquet
Home / Search germplasms
Search germplasms
Search Criteria
Species (ex.maize)
maize
Germplasm
iPG082
Constraints (ex. leafArea < 0.125 & biovolume < 0)
Add constraint
Constraint 1
Variable *
leafArea (square meter)
Operator *
>
Value *
0.4
Constraint 2
Variable *
biovolume (g)
Operator *
>
Value *
400
Filter
Result
Showing 1-1 of 1 item.
Alias Uri Species
1 iPG082 http://www.phenome-fppn.fr/m3p/g/iPG082 http://www.phenome-fppn.fr/id/species/zeamays

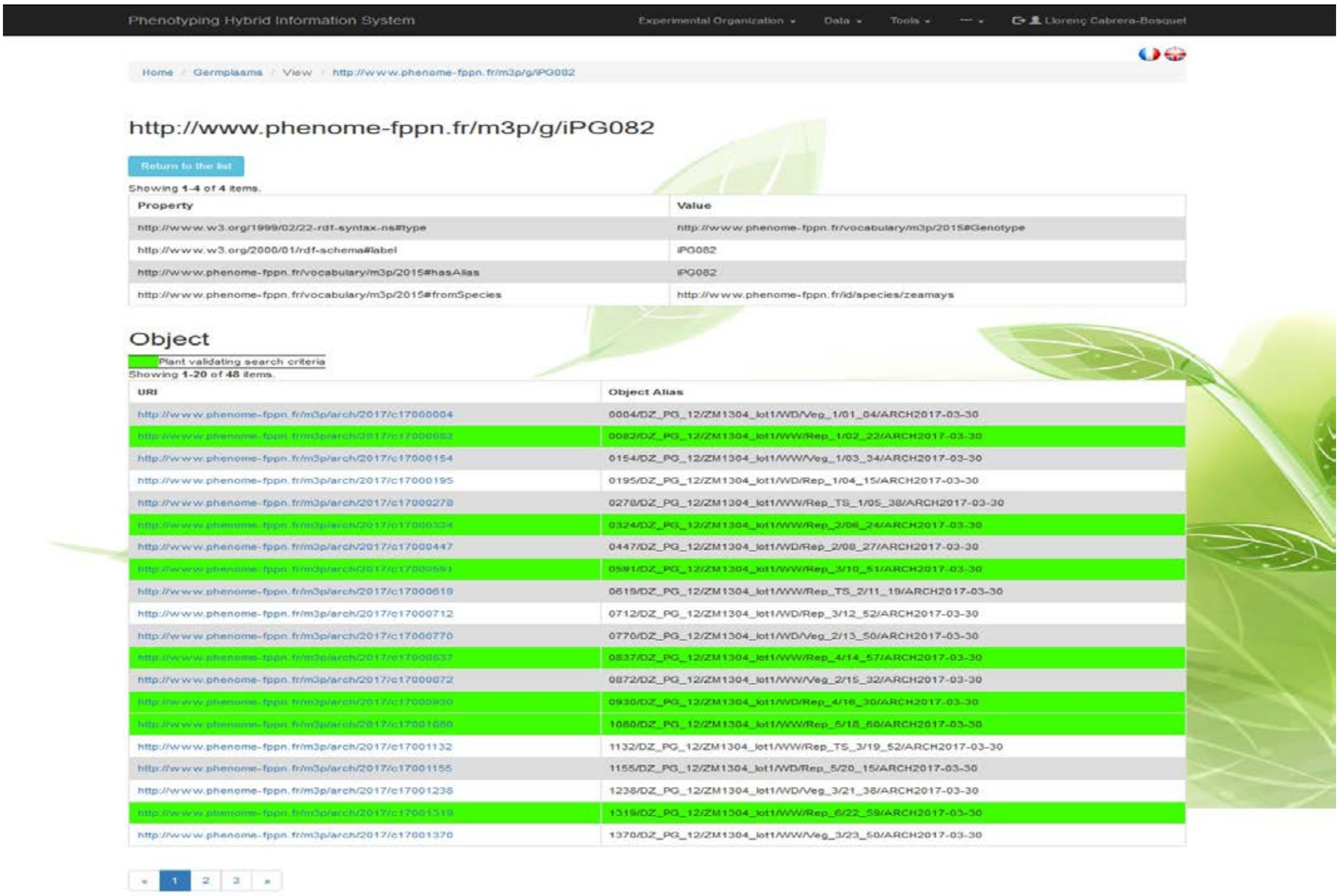
Phenotyping Hybrid Information System
Experimental Organization
Data
Tools
Llorenç Cabrera-Bosquet
Home / Germplasms / View / http://www.phenome-fppn.fr/m3p/g/iPG082
http://www.phenome-fppn.fr/m3p/g/iPG082
Return to the list
Showing 1-4 of 4 items.
Property Value
http://www.w3.org/1999/02/22-rdf-syntax-ns#type http://www.phenome-fppn.fr/vocabulary/m3p/2015#Genotype
http://www.w3.org/2000/01/rdf-schema#label iPG082
http://www.phenome-fppn.fr/vocabulary/m3p/2015#hasAlias iPG082
http://www.phenome-fppn.fr/vocabulary/m3p/2015#fromSpecies http://www.phenome-fppn.fr/id/species/zeamays
Object
Plant validating search criteria
Showing 1-20 of 48 items.
URI Object Alias
http://www.phenome-fppn.fr/m3p/arch/2017/c17000004 0004/DZ_PG_12/ZM1304_lot1/WD/Veg_1/01_04/ARCH2017-03-30
http://www.phenome-fppn.fr/m3p/arch/2017/c17000082 0082/DZ_PG_12/ZM1304_lot1/WW/Rep_1/02_22/ARCH2017-03-30
http://www.phenome-fppn.fr/m3p/arch/2017/c17000154 0154/DZ_PG_12/ZM1304_lot1/WW/Veg_1/03_34/ARCH2017-03-30
http://www.phenome-fppn.fr/m3p/arch/2017/c17000195 0195/DZ_PG_12/ZM1304_lot1/WD/Rep_1/04_15/ARCH2017-03-30
http://www.phenome-fppn.fr/m3p/arch/2017/c17000278 0278/DZ_PG_12/ZM1304_lot1/WW/Rep_TS_1/05_38/ARCH2017-03-30
http://www.phenome-fppn.fr/m3p/arch/2017/c17000324 0324/DZ_PG_12/ZM1304_lot1/WW/Rep_2/06_24/ARCH2017-03-30
http://www.phenome-fppn.fr/m3p/arch/2017/c17000447 0447/DZ_PG_12/ZM1304_lot1/WD/Rep_2/08_27/ARCH2017-03-30
http://www.phenome-fppn.fr/m3p/arch/2017/c17000591 0591/DZ_PG_12/ZM1304_lot1/WW/Rep_3/10_51/ARCH2017-03-30
http://www.phenome-fppn.fr/m3p/arch/2017/c17000619 0619/DZ_PG_12/ZM1304_lot1/WW/Rep_TS_2/11_19/ARCH2017-03-30
http://www.phenome-fppn.fr/m3p/arch/2017/c17000712 0712/DZ_PG_12/ZM1304_lot1/WD/Rep_3/12_52/ARCH2017-03-30
http://www.phenome-fppn.fr/m3p/arch/2017/c17000770 0770/DZ_PG_12/ZM1304_lot1/WD/Veg_2/13_50/ARCH2017-03-30
http://www.phenome-fppn.fr/m3p/arch/2017/c17000837 0837/DZ_PG_12/ZM1304_lot1/WW/Rep_4/14_57/ARCH2017-03-30
http://www.phenome-fppn.fr/m3p/arch/2017/c17000872 0872/DZ_PG_12/ZM1304_lot1/WW/Veg_2/15_32/ARCH2017-03-30
http://www.phenome-fppn.fr/m3p/arch/2017/c17000930 0930/DZ_PG_12/ZM1304_lot1/WD/Rep_4/16_30/ARCH2017-03-30
http://www.phenome-fppn.fr/m3p/arch/2017/c17001080 1080/DZ_PG_12/ZM1304_lot1/WW/Rep_5/18_60/ARCH2017-03-30
http://www.phenome-fppn.fr/m3p/arch/2017/c17001132 1132/DZ_PG_12/ZM1304_lot1/WW/Rep_TS_3/19_52/ARCH2017-03-30
http://www.phenome-fppn.fr/m3p/arch/2017/c17001155 1155/DZ_PG_12/ZM1304_lot1/WD/Rep_5/20_15/ARCH2017-03-30
http://www.phenome-fppn.fr/m3p/arch/2017/c17001238 1238/DZ_PG_12/ZM1304_lot1/WD/Veg_3/21_38/ARCH2017-03-30
http://www.phenome-fppn.fr/m3p/arch/2017/c17001319 1319/DZ_PG_12/ZM1304_lot1/WW/Rep_6/22_59/ARCH2017-03-30
http://www.phenome-fppn.fr/m3p/arch/2017/c17001370 1370/DZ_PG_12/ZM1304_lot1/WW/Veg_3/23_50/ARCH2017-03-30
« 1 2 3 »
