# Supplementary material for: Dealing with multi‐source and multi‐scale information in plant phenomics: the ontology‐driven Phenotyping Hybrid Information System
Source: New Phytol. 2018 Aug 28;221(1):588–601. doi: 10.1111/nph.15385 (PMC6585972; doi:10.1111/nph.15385)
Supplement: Supplementary file 2 — Notes S2 Annotations and events. [file NPH-221-588-s002.pdf]

Events and Annotations

The Events menu allows searching and visualising events and annotations performed in the different objects (e.g. plants, sensors, experiments) declared in the information system.

Different filters can be applied based on `Dates`, `Type`, `Description` of events or the `Concern` or `Alias` object (Fig. 1). As an example, searching for plant 795 (having the URI `m3p:/arch/2017/c17000795`) in the **Concern** box, results in seven different events describing:

- `oevv:Sowing` => sowing pf the plant
- `oevv:Thinning` => installation of plant tutors
- `oevv:Fertilization` => fertilizer application
- `oeev:Stacking` => installation of plant tutors
- `oevv:MoveTo` => displacement event
- `oevv:StuckPlant` => an incident ocurred during imaging
- `oevv:Harvesting` => harvesting of the plant

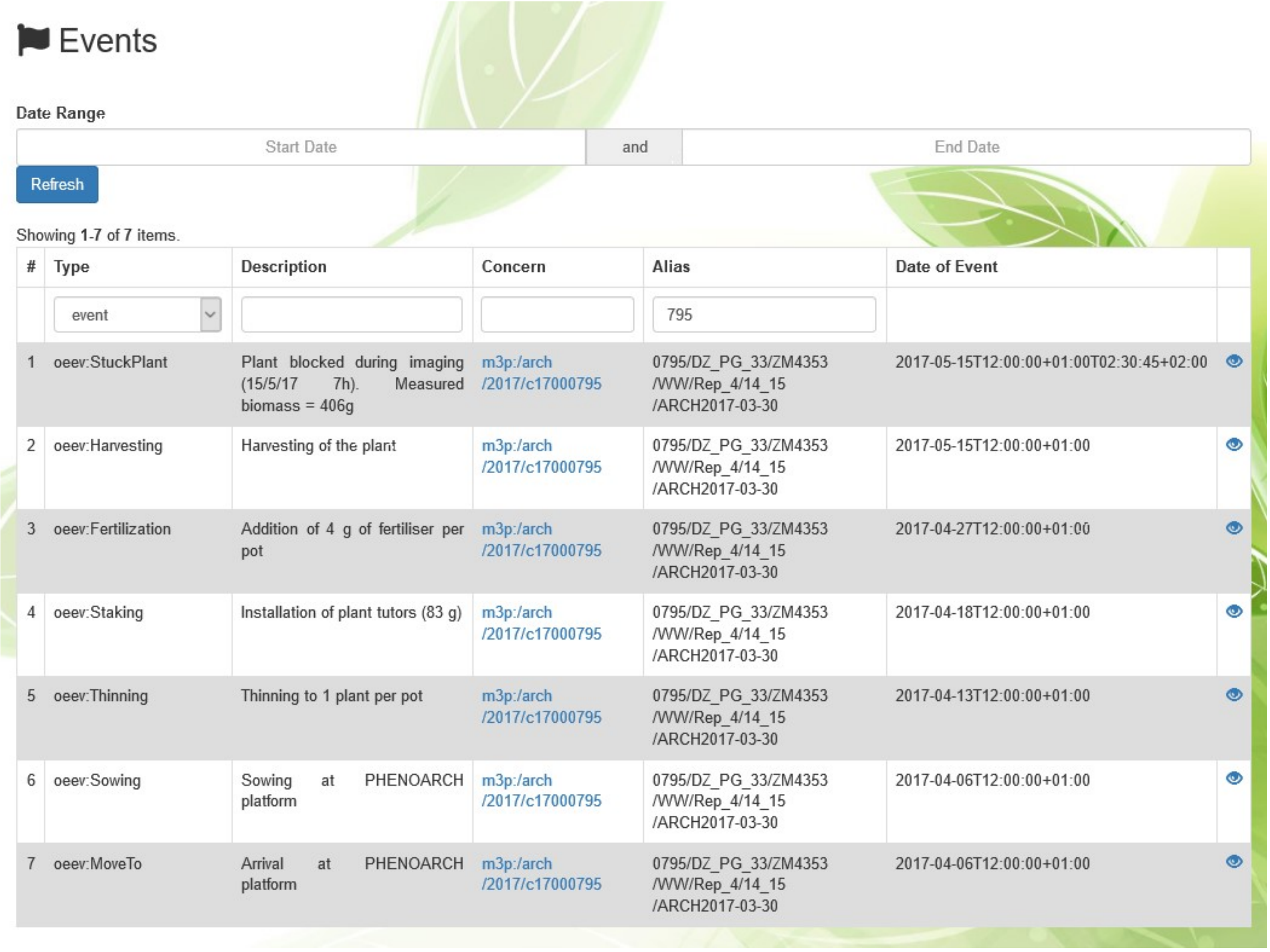

Events

Date Range

Start Date and End Date

Refresh

Showing 1-7 of 7 items.

#	Type	Description	Concern	Alias	Date of Event	
	event			795		
1	oeev:StuckPlant	Plant blocked during imaging (15/5/17 7h). Measured biomass = 406g	m3p:/arch /2017/c17000795	0795/DZ_PG_33/ZM4353 /WW/Rep_4/14_15 /ARCH2017-03-30	2017-05-15T12:00:00+01:00T02:30:45+02:00	
2	oeev:Harvesting	Harvesting of the plant	m3p:/arch /2017/c17000795	0795/DZ_PG_33/ZM4353 /WW/Rep_4/14_15 /ARCH2017-03-30	2017-05-15T12:00:00+01:00	
3	oeev:Fertilization	Addition of 4 g of fertiliser per pot	m3p:/arch /2017/c17000795	0795/DZ_PG_33/ZM4353 /WW/Rep_4/14_15 /ARCH2017-03-30	2017-04-27T12:00:00+01:00	
4	oeev:Staking	Installation of plant tutors (83 g)	m3p:/arch /2017/c17000795	0795/DZ_PG_33/ZM4353 /WW/Rep_4/14_15 /ARCH2017-03-30	2017-04-18T12:00:00+01:00	
5	oeev:Thinning	Thinning to 1 plant per pot	m3p:/arch /2017/c17000795	0795/DZ_PG_33/ZM4353 /WW/Rep_4/14_15 /ARCH2017-03-30	2017-04-13T12:00:00+01:00	
6	oeev:Sowing	Sowing at PHENOARCH platform	m3p:/arch /2017/c17000795	0795/DZ_PG_33/ZM4353 /WW/Rep_4/14_15 /ARCH2017-03-30	2017-04-06T12:00:00+01:00	
7	oeev:MoveTo	Arrival at PHENOARCH platform	m3p:/arch /2017/c17000795	0795/DZ_PG_33/ZM4353 /WW/Rep_4/14_15 /ARCH2017-03-30	2017-04-06T12:00:00+01:00	

Detailed information of each event can be displayed including **RDF triples** (Fig. 2). For instance the event `http://www.phenome-fppn.fr/id/event/5878e232-c4b8-4fb8-b705-1988540dcfd2` describing the `Harvesting` of plant 795 is formulated as:

http://www.phenome-fppn.fr/id/event/efb8946f-799d-4c02-b425-3fbf637c122b

Add annotation | Browse Triplestore

Type	oeev:Harvesting
Description	Harvesting of the plant
Author	http://www.phenome-fppn.fr/id/agent/lcabrera
Date of Event	2017-05-15T12:00:00+01:00
Concern	m3p:/arch/2017/c17000795
Alias	0795/DZ_PG_33/ZM4353/WW/Rep_4/14_15/ARCH2017-03-30

http://www.phenome-fppn.fr/id/event/efb8946f-799d-4c02-b425-3fbf637c122b

Add annotation | Add Event

Showing **1-4** of **4** items.

#	Subject	Predicate	Object or Litteral
	Type in some characters...	Type in some characters...	Type in some characters...
1	annotation:/fcdcb1ce-8e44-4808-adc4-0515a3edf25f	oa:hasTarget	**http://www.phenome-fppn.fr/id/event/efb8946f-799d-4c02-b425-3fbf637c122b**
2	**http://www.phenome-fppn.fr/id/event/efb8946f-799d-4c02-b425-3fbf637c122b**	oeev:concern	m3p:/arch/2017/c17000795
3	**http://www.phenome-fppn.fr/id/event/efb8946f-799d-4c02-b425-3fbf637c122b**	rdf:type	oeev:Harvesting
4	**http://www.phenome-fppn.fr/id/event/efb8946f-799d-4c02-b425-3fbf637c122b**	time:hasTime	instant:/06a68a04-d669-4388-90b2-f6dc3bf206fa

where is of type harvesting (**rdf:type**), concerns plant 795 (**event:concern**), and has and associated time stamp (**time:hasTime**). Details of the timings of the event (**time:inXSDDateTimeStamp**) and its annotation are described in Fig. 3 as:

http://www.phenome-fppn.fr/m3p/arch/2018/tic18_3efdc550-5294-479d-b8ec-ef17e19b2ce6

Add annotation

Showing **1-4** of **4** items.

#	Subject	Predicate	Object or Litteral
	Type in some characters	Type in some characters	Type in some characters
1	m3p:/arch/2018/evc18_6851fb51-efa6-44ff-bc4d-bf1cfa98f576	time:hasTime	**http://www.phenome-fppn.fr/m3p/arch /2018/tic18_3efdc550-5294-479d-b8ec-ef17e19b2ce6**
2	**http://www.phenome-fppn.fr/m3p/arch /2018/tic18_3efdc550-5294-479d-b8ec-ef17e19b2ce6**	rdf:type	time:Instant
3	**http://www.phenome-fppn.fr/m3p/arch /2018/tic18_3efdc550-5294-479d-b8ec-ef17e19b2ce6**	time:inXSDDateTimeStamp	2017-05-15T12:00:00+01:00
4	**http://www.phenome-fppn.fr/m3p/arch /2018/tic18_3efdc550-5294-479d-b8ec-ef17e19b2ce6**	dcterms:created	2018-03-28T11:41:16.418771

The harvesting event `http://www.phenome-fppn.fr/id/event/5878e232-c4b8-4fb8-b7051988540dcfd2` concerning the plant 795 is associated to the annotation `http://www.phenomefppn.fr/id/annotation/f3fb3f7c-b4a0-4531-b3de-5cfd8b4b2b28` ` via **oa:hasTarget**. Details of the annotation, such as the type **oa:Annotation**, the **oa:bodyValue** '*Harvesting of the plant*' and the time that event was annotated (**dcterms:created**) and the person who did the annotation (**dcterms:creator**) are described below (Fig. 4):

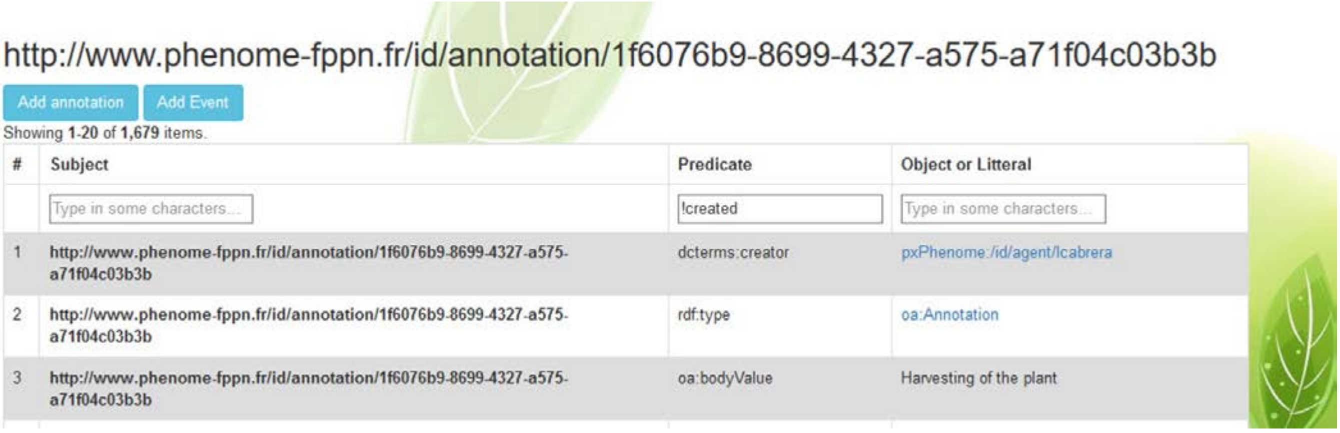
http://www.phenome-fppn.fr/id/annotation/1f6076b9-8699-4327-a575-a71f04c03b3b

Add annotation | Add Event

Showing 1-20 of 1,679 items.

#	Subject	Predicate	Object or Litteral
	Type in some characters...	!created	Type in some characters...
1	http://www.phenome-fppn.fr/id/annotation/1f6076b9-8699-4327-a575-a71f04c03b3b	dcterms:creator	pxPhenome:/id/agent/lcabrera
2	http://www.phenome-fppn.fr/id/annotation/1f6076b9-8699-4327-a575-a71f04c03b3b	rdf:type	oa:Annotation
3	http://www.phenome-fppn.fr/id/annotation/1f6076b9-8699-4327-a575-a71f04c03b3b	oa:bodyValue	Harvesting of the plant

Annotations

Annotations are mainly guided by the **OEEv ontology** and the **Web Annotation Data Model** that allows annotating any object declared in the system and assigning motivation and purpose attributes to annotations (e.g. oa:describing, oa:identifying, oa:linking, oa: replying, etc.).

```
    'http://www.w3.org/ns/oa#describing' => 'oa:describing',
    'http://www.w3.org/ns/oa#identifying' => 'oa:identifying',
    'http://www.w3.org/ns/oa#moderating' => 'oa:moderating',
    'http://www.w3.org/ns/oa#assessing' => 'oa:assessing',
    'http://www.w3.org/ns/oa#bookmarking' => 'oa:bookmarking',
    'http://www.w3.org/ns/oa#classifying' => 'oa:classifying',
    'http://www.w3.org/ns/oa#commenting' => 'oa:commenting',
    'http://www.w3.org/ns/oa#editing' => 'oa:editing',
    'http://www.w3.org/ns/oa#highlighting' => 'oa:highlighting',
    'http://www.w3.org/ns/oa#linking' => 'oa:linking',
    'http://www.w3.org/ns/oa#questioning' => 'oa:questioning',
    'http://www.w3.org/ns/oa#replying' => 'oa:replying',
    'http://www.w3.org/ns/oa#tagging' => 'oa:tagging',
```

Annotation

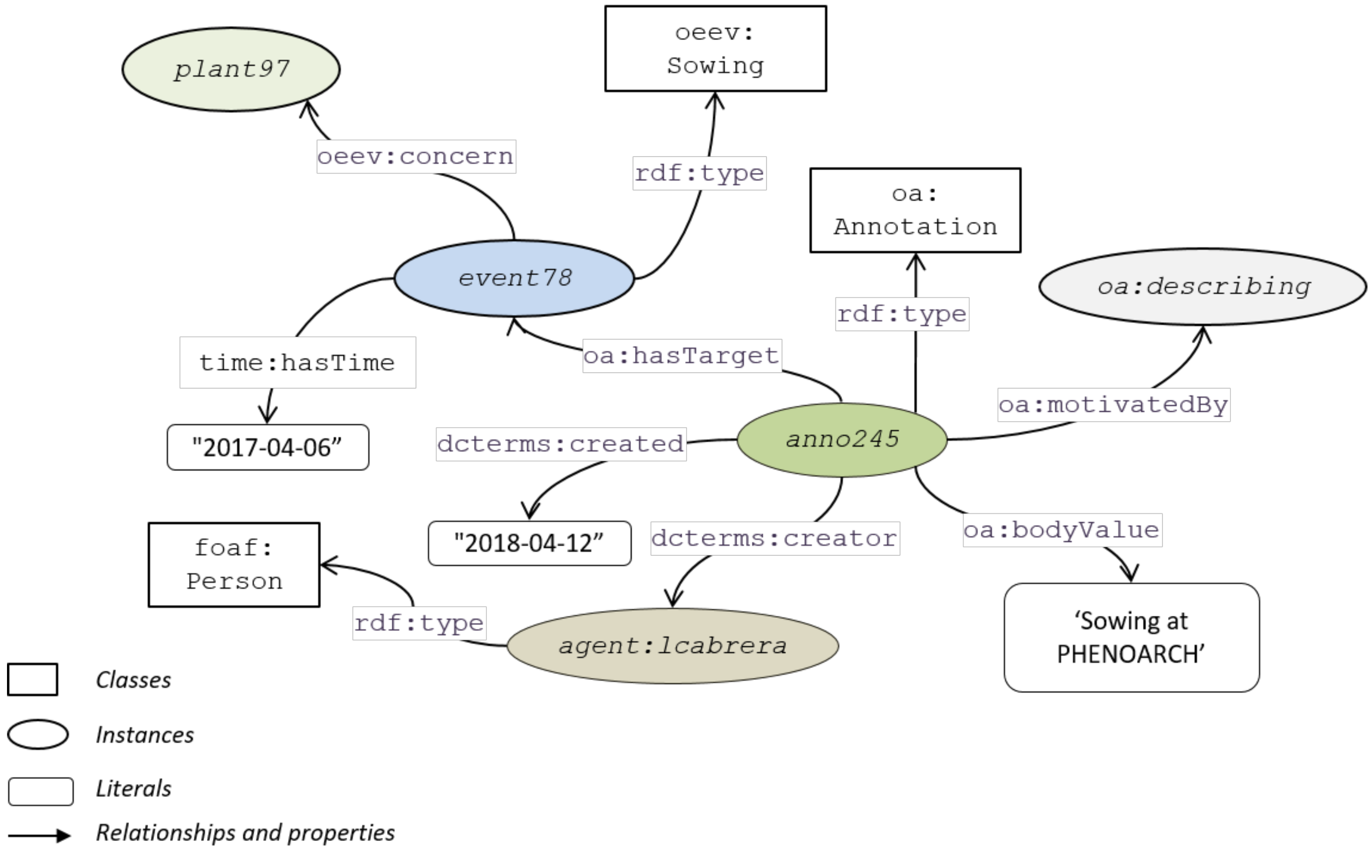

Dublin Core and **foaf** specifications such as *dcterms:created*, *dcterms:creator*, *foaf:Agent* are also used. For instance, the agent `agent:lcabrera`, is a `Person` member of the group `PHENOARCH-ADMIN` that has a number of attributes such as `firstName`, `lastName`, `mbox`, etc. The Agent `agent:lcabrera` is the author (dcterms:creator) of the annotation#245 (Fig. 6).

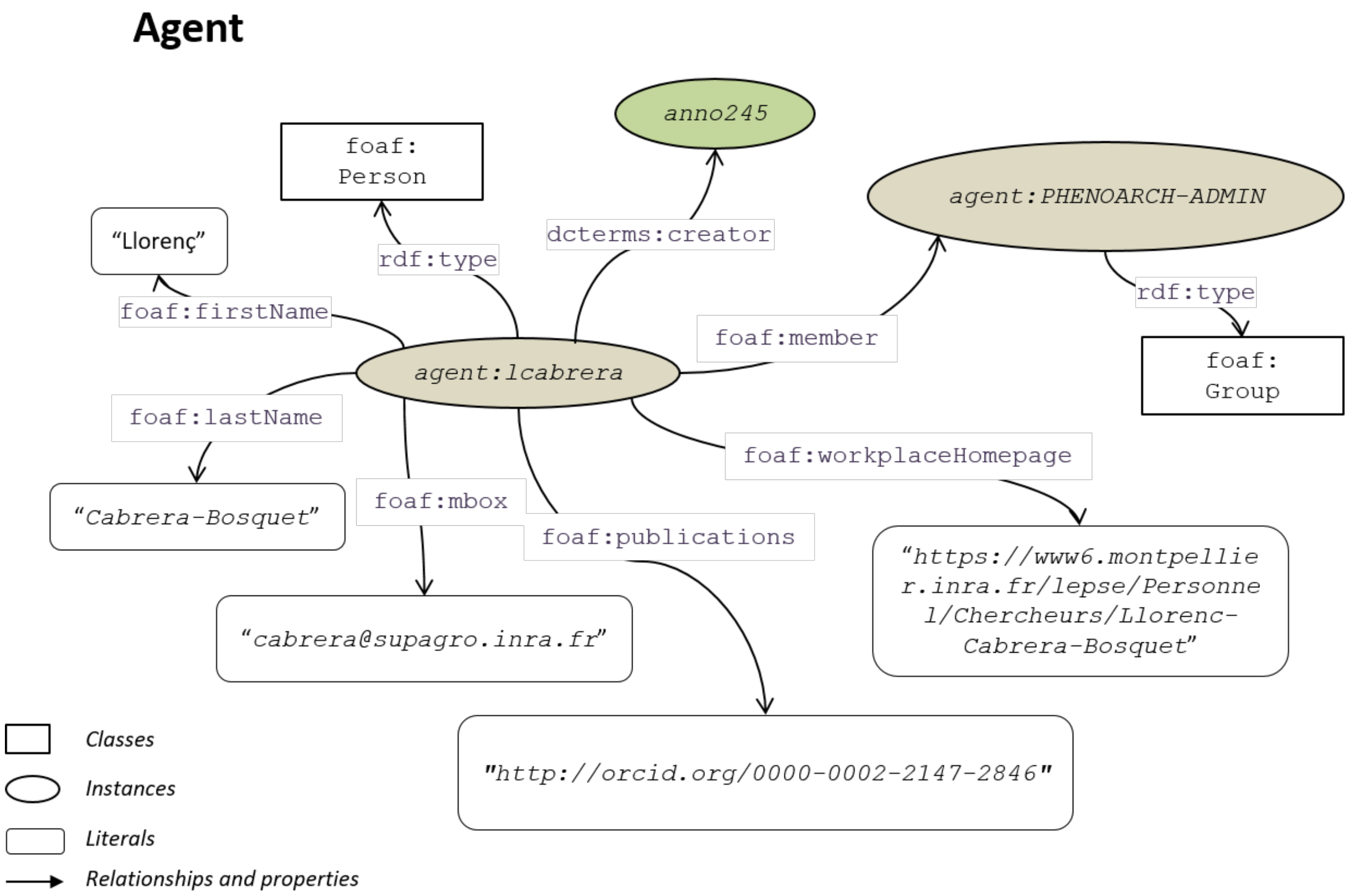


```
The different prefixes stand for:
- oeev: <http://www.phenome-fppn.fr/vocabulary/2018/oeev#>
- oa: <http://www.w3.org/ns/oa#>
- foaf: <http://xmlns.com/foaf/spec/>
- rdf: <http://www.w3.org/1999/02/22-rdf-syntax-ns#>
- time: <http://www.w3.org/2006/time#>
- dcterms: <http://purl.org/dc/terms/>
- agent: <http://www.phenome-fppn.fr/id/agent/>
```

Annotations and events can be added into any object (e.g. plant, device, experiment, event) by clicking on the `add annotation` and `add event` buttons present in the different menus:

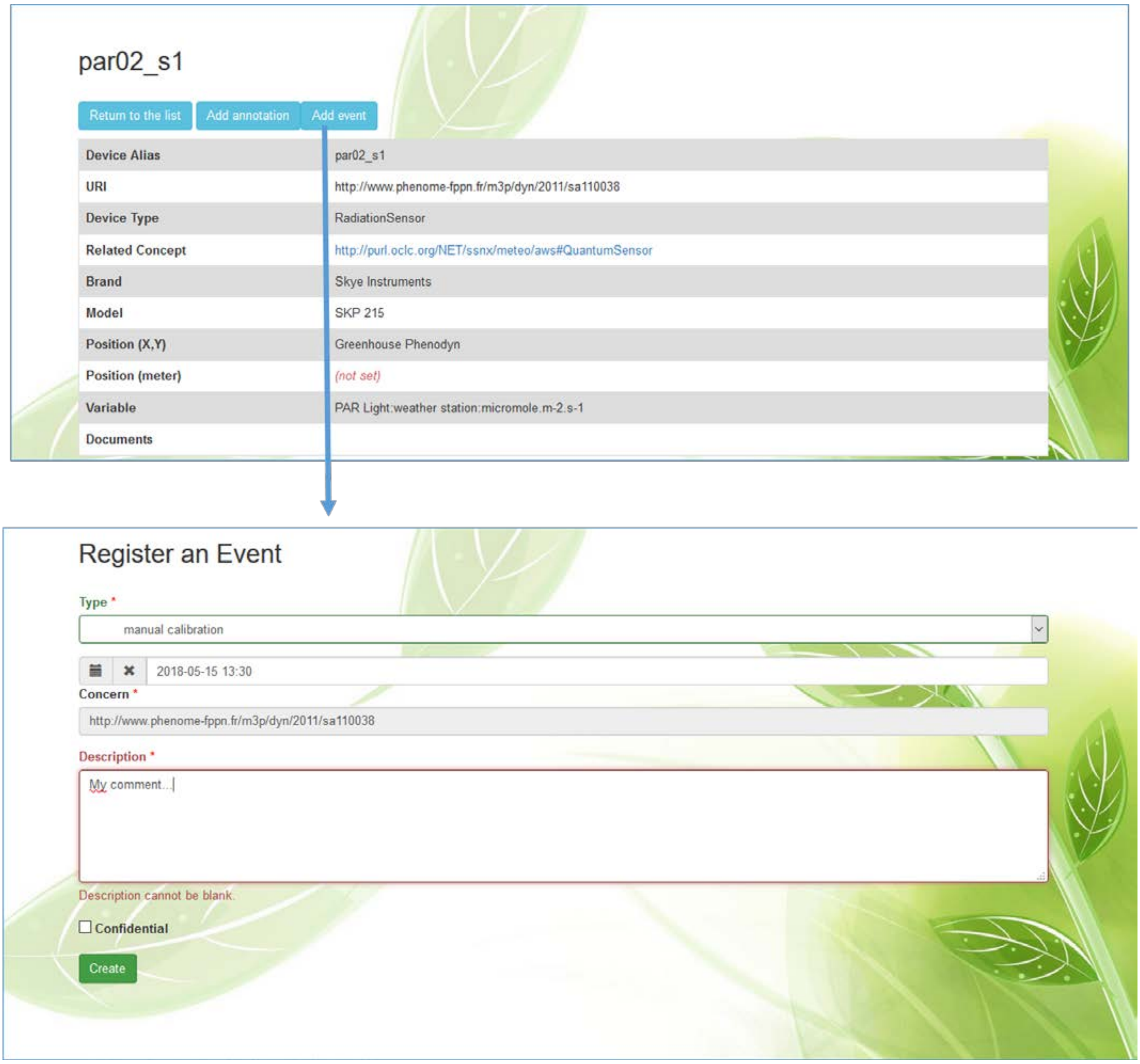

Use cases

Lodging of plot

The following semantic graph represents how **OEEv** ontology si used for representing a lodging event associated to ***plot97*** (Fig. 7).

In the example presented here, the ***event54*** that concerns ***plot97*** is of type ***Lodging*** and occurred the **"2017-06-29"**. ***event356*** is associated to the ***ann4807*** which is of type ***Annotation*** and is motivated by describing (***oa:describing***). This annotation, has a creator (***Agent1***), a date of annotation (**"2017-08-10"**) and a body value describing the event (**"Plots lodged after the storm"**).

Lodging of a plot

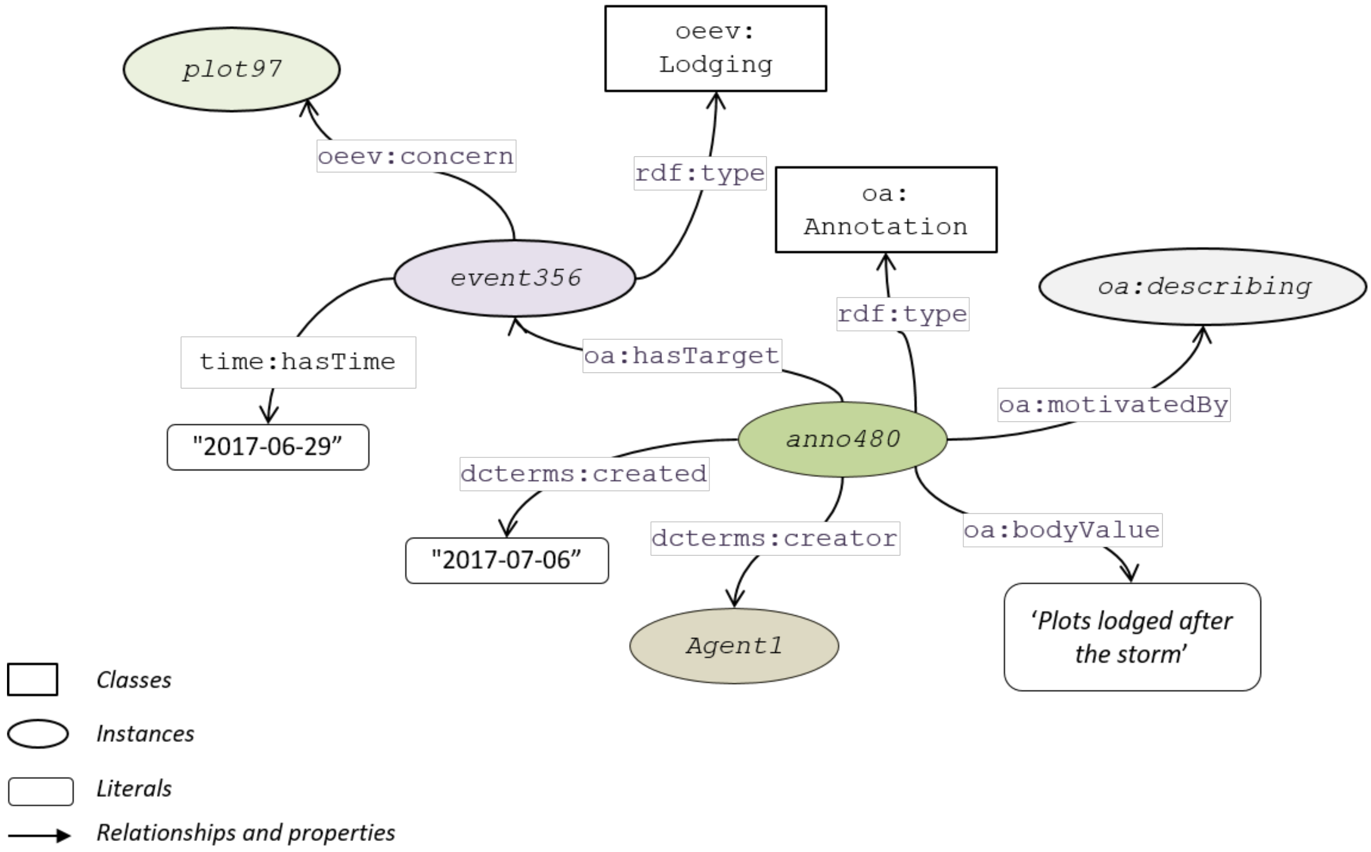

- Instances are depicted as coloured ellipses.
- Classes are depicted as white rectangles.
- Literals are depicted as white lozenges.
- Relationships and properties are depicted as black lines.

For better legibility objects are labelled with shortened alias names rather than using full URIs:

- ***plot97***: http://www.phenome-fppn.fr/diaphen/2017/o17000097 ***Agent1***: http://www.phenome-
- fppn.fr/id/agent/rchapuis ***event356***: http://www.phenome-fppn.fr/id/event/cafa1c09-2cbf-409f-
- 82f9-9f20496e3eb9 ***anno480***: http://www.phenome-fppn.fr/id/annotation/469bca50-10f9-4deb-
- 9b3a-6ae7476306cb

Note that not all objects and events associated to ***plot97*** are represented in this graph. Fulls details can be found in the PHIS user interface by searching for plot 97 in the different menus (e.g. **Events**, **Scientific objects**).

The event can be displayed in the web user interface by filtering by lodging and plot97 in the Events menu. Further information can be displayed by clicking on the `Browse Triplestore` button:

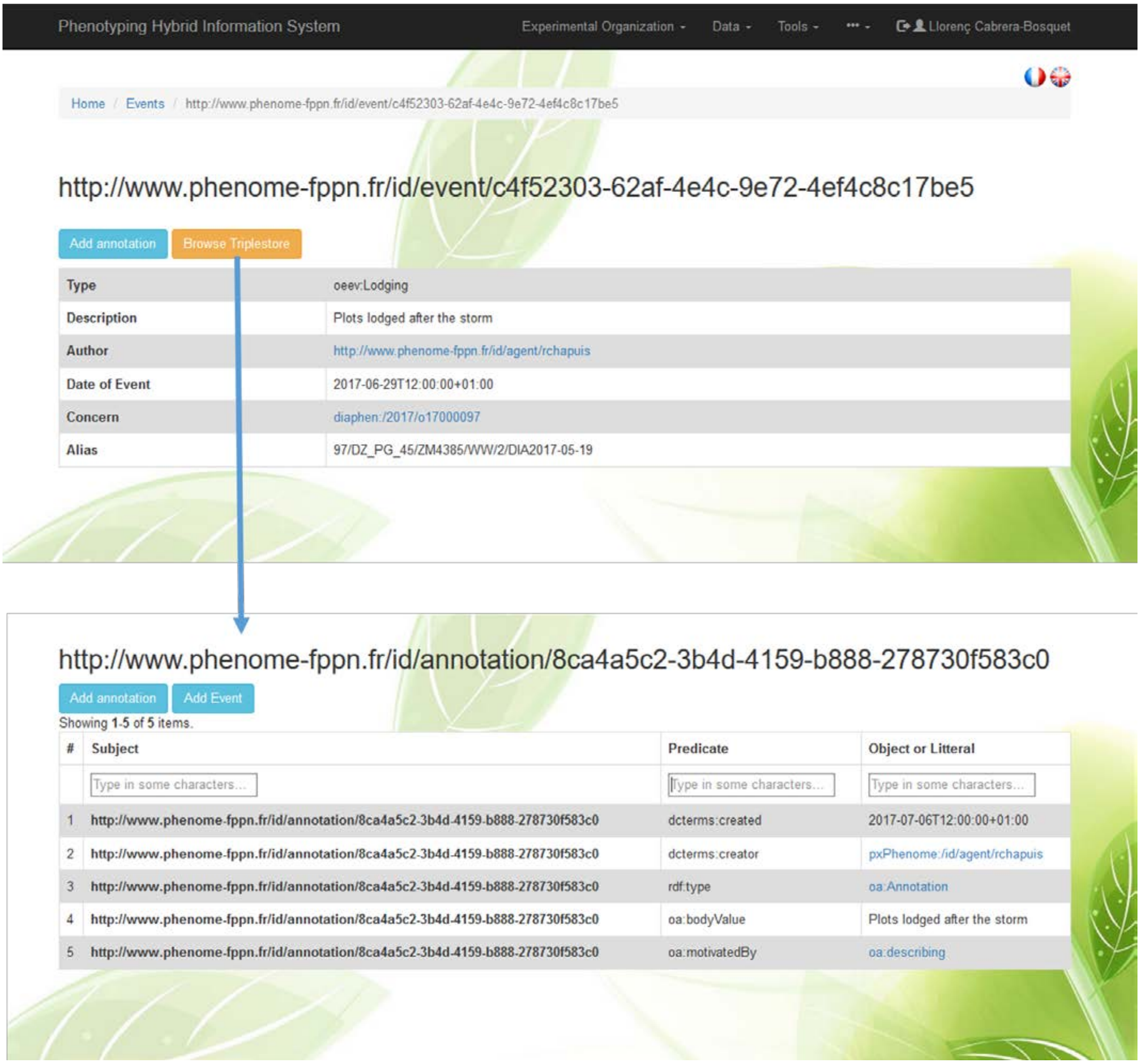

Plant blocked in imaging cabin

The following semantic graph represents how **OEEv** ontology is used for representing an incident during imaging acquisition associated to ***plant795*** (Fig. 10).

In the example presented here, the ***event849*** that concerns ***plant795*** is of type ***PlantStuck*** and occurred the **"2017-05-15"**. ***event849*** is associated to the ***anno86*** which is of type ***Annotation*** and is motivated by describing (***oa:describing***). This annotation, has a creator (***Agent2***), a date of annotation (**"2017-05-26"**) and a body value describing the event (**"Fallen plant during imaging"**).

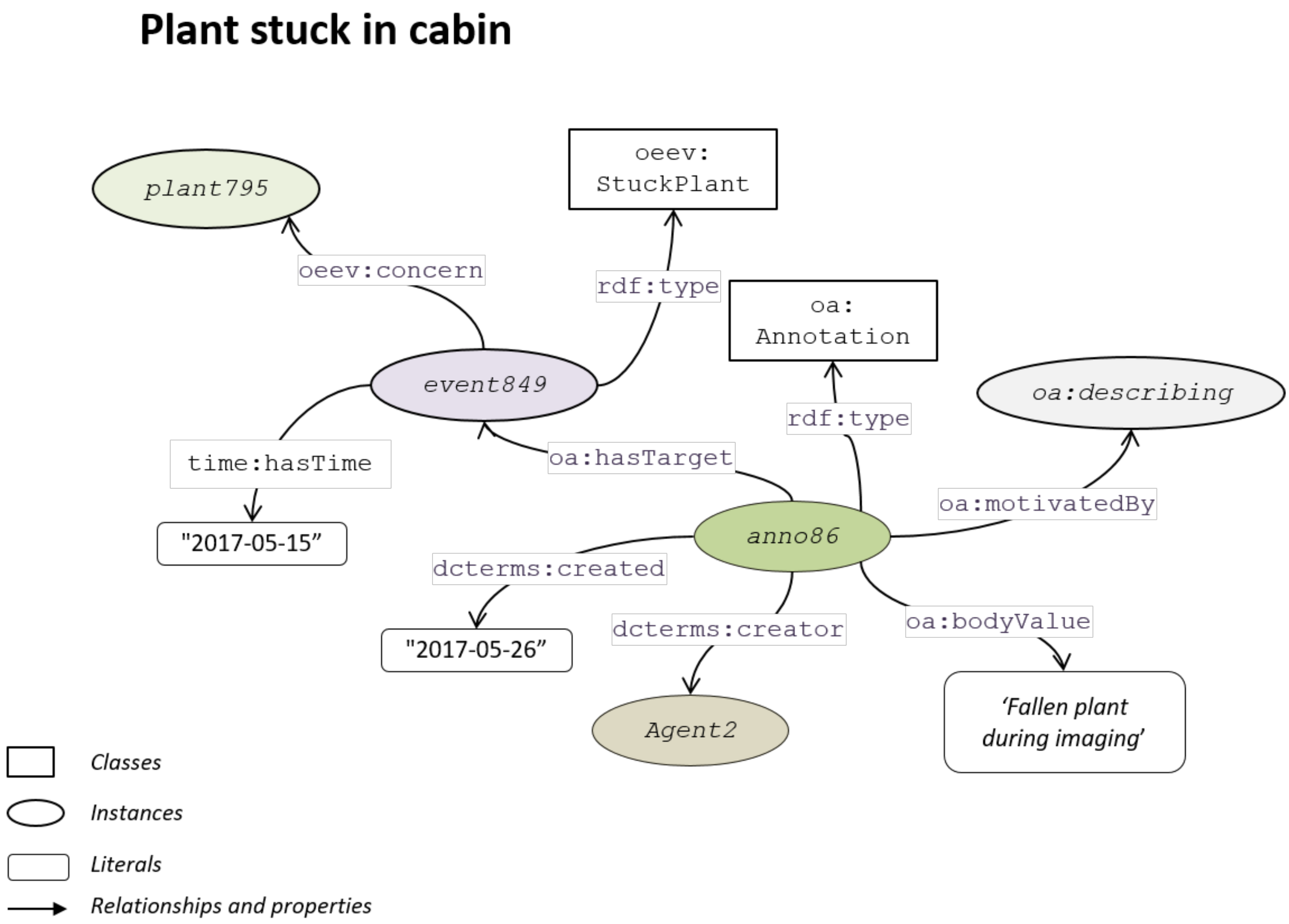

- Instances are depicted as coloured ellipses.
- Classes are depicted as white rectangles.
- Literals are depicted as white lozenges.
- Relationships and properties are depicted as black lines.

For better legibility objects are labelled with shortened alias names rather than using full URIs:

- ***plant795***: http://www.phenome-fppn.fr/m3p/arch/2017/c17000795 ***Agents***:
- http://www.phenome-fppn.fr/id/agent/lcabrera ***event849***: http://www.phenome-
- fppn.fr/id/event/bcbb7240-34bc-4bc3-8ff3-31911e4b6aa7 ***anno86***: http://www.phenome-
- fppn.fr/id/annotation:4aac3076-b542-4f6e-92e1-9d350dd3eec5

Note that not all objects and events associated to ***plant795*** are represented in this graph. Fulls details can be found in the PHIS user interface by searching for plant 97 in the different menus (e.g. **Events**, **Scientific objects**).

Suspected seed contamination

The following semantic graph represents how **OEEv** ontology is used for annotating an annomaly detected during data processing (i.e. suspected seed contamination) (Fig. 11).

The ***anno338*** which is of type ***Annotation*** and is motivated by tagging (***oa:tagging***) has as target ***plant816***. The ***anno338*** has a creator (***Agent2***), a date of annotation (**"2018-05-17 11:02:39"**) and a body value describing the event (**"Suspected seed contamination"**).

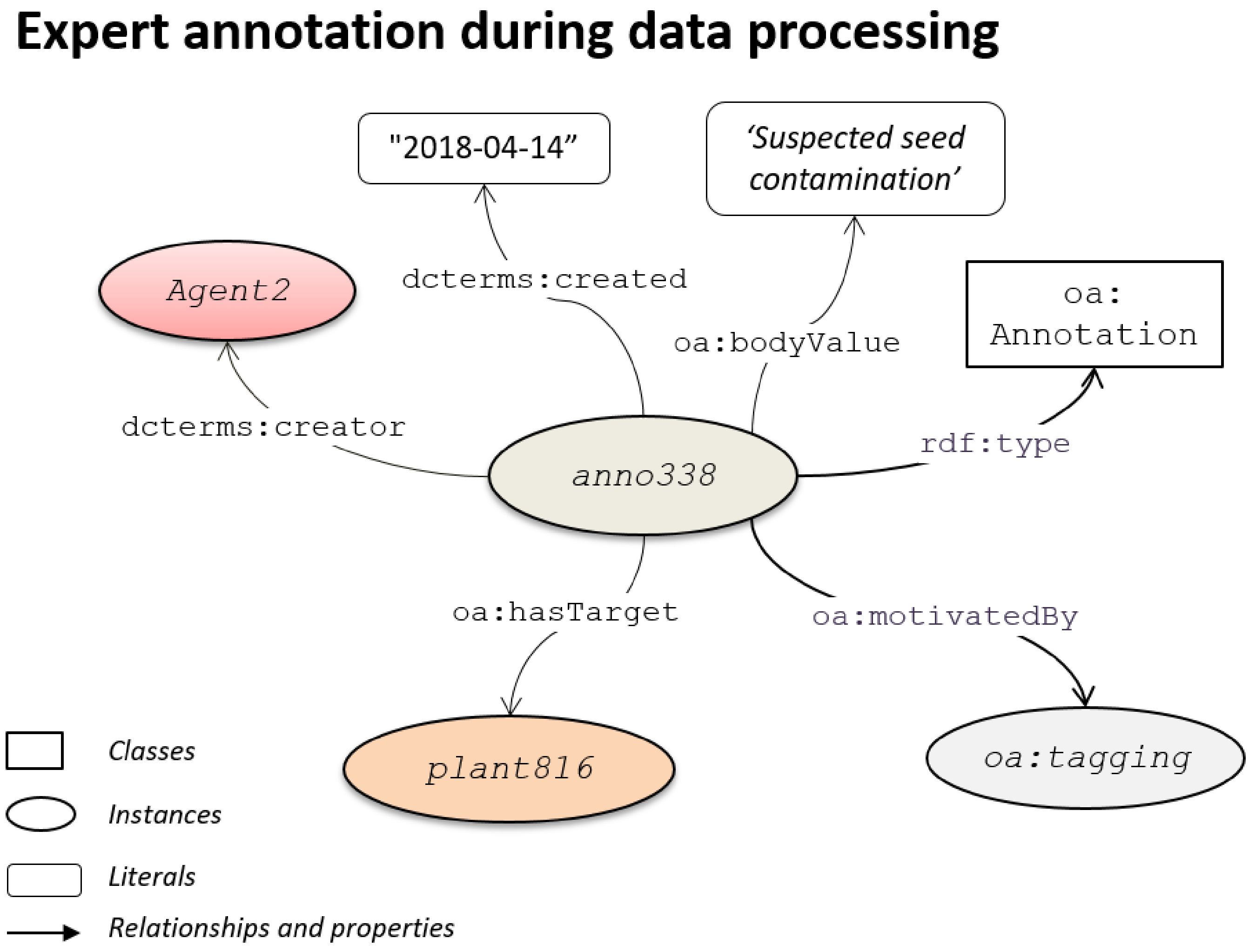

- Instances are depicted as coloured ellipses.
- Classes are depicted as white rectangles.
- Literals are depicted as white lozenges.
- Relationships and properties are depicted as black lines.

For better legibility objects are labelled with shortened alias names rather than using full URIs:

- ***plant816***: http://www.phenome-fppn.fr/m3p/arch/2017/c17000816
- ***Agent2***: http://www.phenome-fppn.fr/id/agent/lcabrera
- ***anno338***: http://www.phenome-fppn.fr/id/annotation/5b0bd064452b1

Note that not all objects and events associated to ***plant816*** are represented in this graph. Fulls details can be found in the PHIS user interface by searching for plant 97 in the different menus (e.g. **Events**, **Scientific objects**).

Camera Dysfunction

In this example the target is a RGB camera that suffered a dysfunction (Fig. 12):

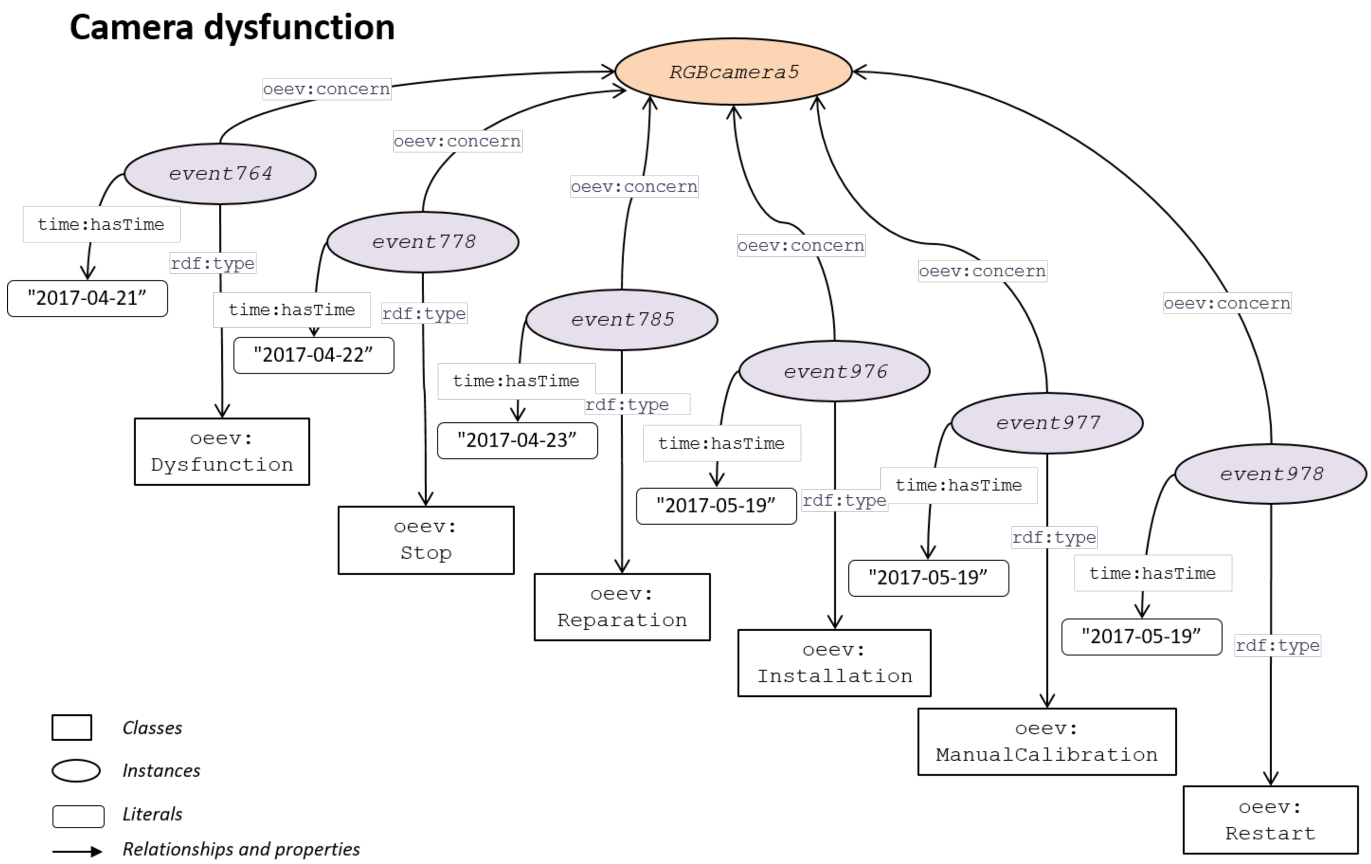

As represenetd in the semantic graph the ***RGBcamera5*** suffered as ***Dysfucntion*** the "2017-04-21". The "2017-04-22", image acquisition was stopped in cabin 5 as represented with the event ***Stop***. The "2017-04-23" the camera was sent to the factory for ***Reparation***. The "2017-05-19", the repaired camera was reinstalled and calibrated before being put in service again, as represented by the ***Installation***, ***ManualCalibration*** and ***Restart*** events.

For better legibility objects are labelled with shortened alias names rather than using full URIs:

- ***RGBcamera5***: http://www.phenome-fppn.fr/m3p/arch/2018/ac180011 ***event764***:
- http://www.phenome-fppn.fr/id/event/dda84a5e-8783-4f60-8d80-d5a3ccb36b25 ***event778***:
- http://www.phenome-fppn.fr/id/event/8be63f29-b3ba-4d0a-b40b-775d6119b167
- ***event785***: http://www.phenome-fppn.fr/id/event/39a5a56a-bec8-4a35-a4dc-35d9c13b480f
- ***event976***: http://www.phenome-fppn.fr/id/event/2ed84816-516f-4621-b7b9-face4acf7de4
- ***event977***: http://www.phenome-fppn.fr/id/event/2ed6d628-c076-42d1-b8ba-014eaade4701
- ***event978***: http://www.phenome-fppn.fr/id/event/05dc3267-9cce-4bce-8175-a7b2e04d663f

Only event types and dates are represented. Full details can be consulted online in the Events or Device menu by searching for *camera* or http://www.phenome-fppn.fr/m3p/arch/2018/ac180011 (Fig. 13).

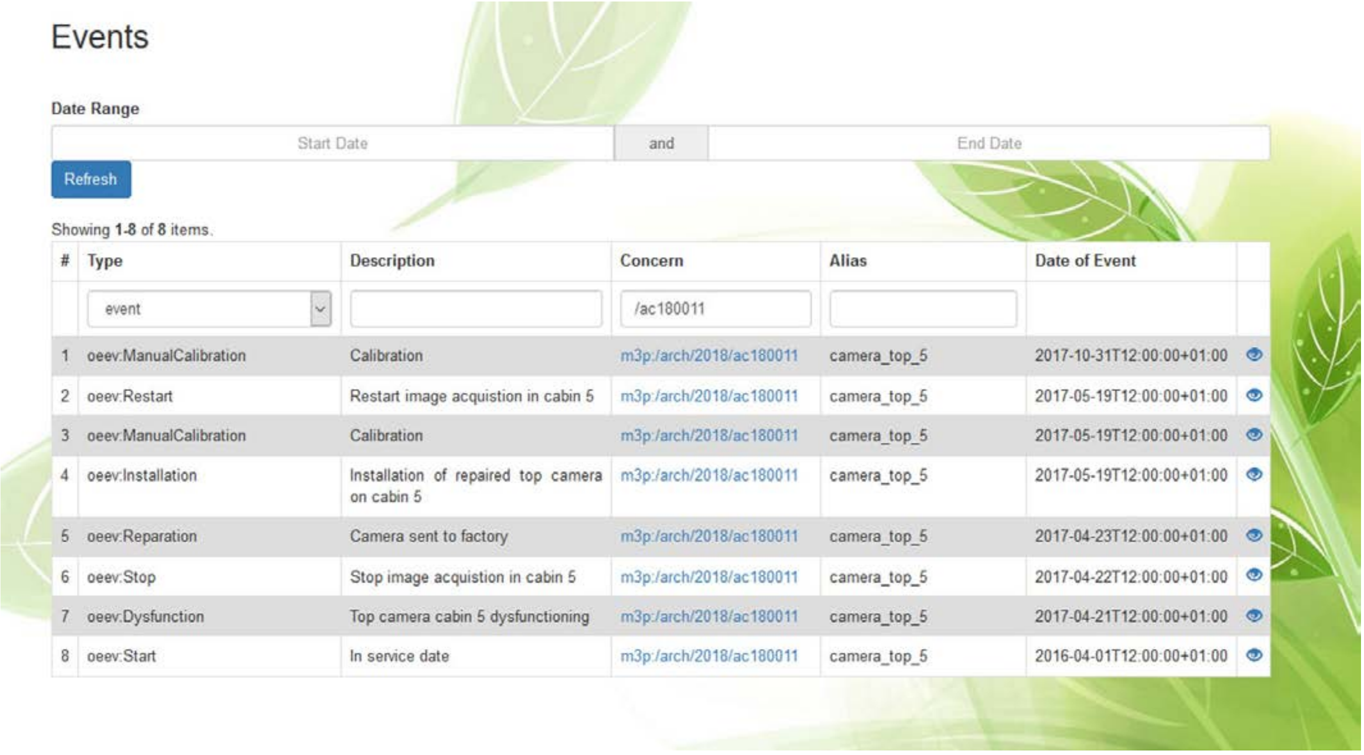

Events

Date Range

Start Date and End Date

Refresh

Showing 1-8 of 8 items.

#	Type	Description	Concern	Alias	Date of Event
	event		/ac180011		
1	oeev:ManualCalibration	Calibration	m3p:/arch/2018/ac180011	camera_top_5	2017-10-31T12:00:00+01:00
2	oeev:Restart	Restart image acquistion in cabin 5	m3p:/arch/2018/ac180011	camera_top_5	2017-05-19T12:00:00+01:00
3	oeev:ManualCalibration	Calibration	m3p:/arch/2018/ac180011	camera_top_5	2017-05-19T12:00:00+01:00
4	oeev:Installation	Installation of repaired top camera on cabin 5	m3p:/arch/2018/ac180011	camera_top_5	2017-05-19T12:00:00+01:00
5	oeev:Reparation	Camera sent to factory	m3p:/arch/2018/ac180011	camera_top_5	2017-04-23T12:00:00+01:00
6	oeev:Stop	Stop image acquistion in cabin 5	m3p:/arch/2018/ac180011	camera_top_5	2017-04-22T12:00:00+01:00
7	oeev:Dysfunction	Top camera cabin 5 dysfunctioning	m3p:/arch/2018/ac180011	camera_top_5	2017-04-21T12:00:00+01:00
8	oeev:Start	In service date	m3p:/arch/2018/ac180011	camera_top_5	2016-04-01T12:00:00+01:00

Leaf Sampling

In this example, the sapmling of a leaf of plant 60 is represented (Fig. 14):

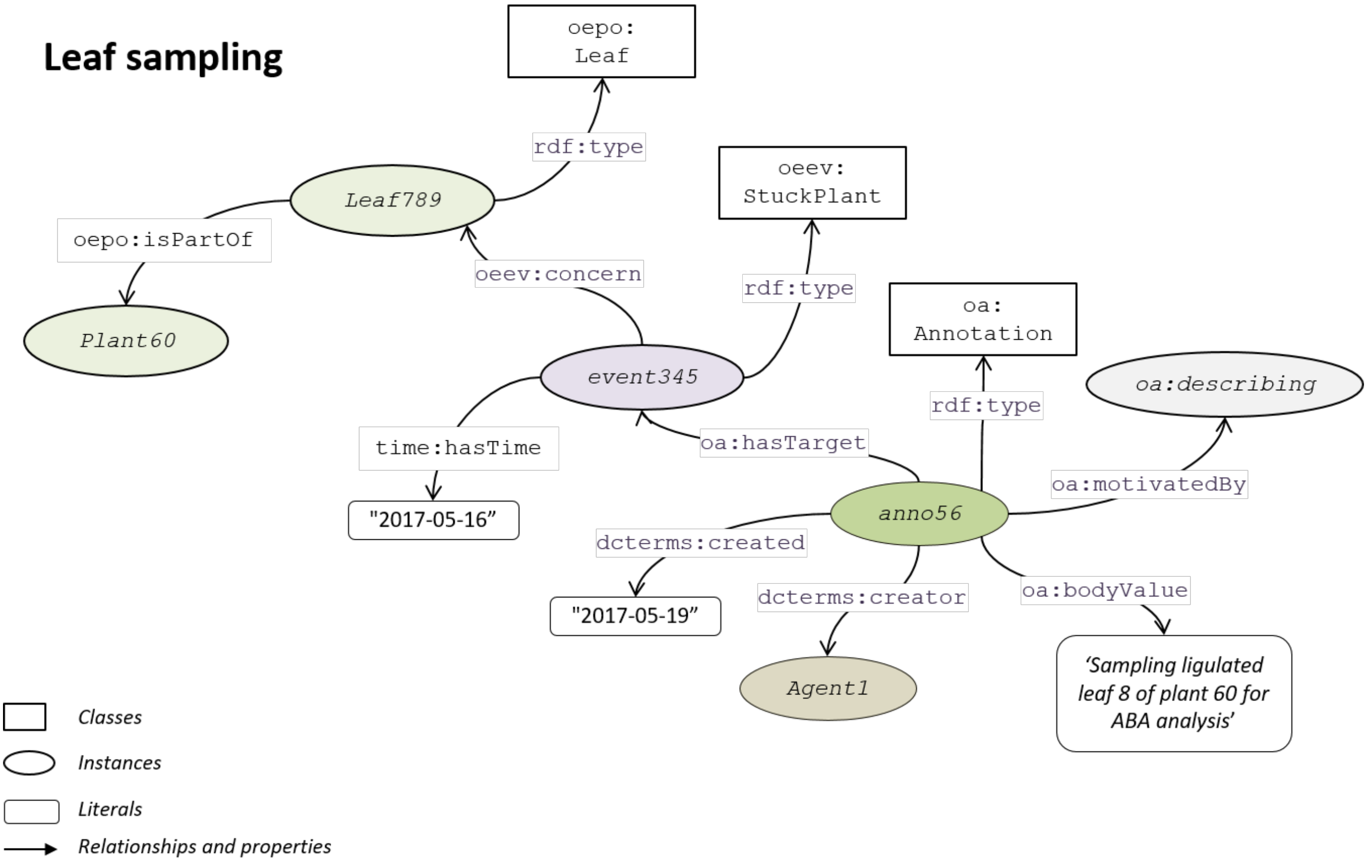

The real example concerning leaf `http://www.phenome-fppn.fr/m3p/arch/2017/lc17000060` can be consulted online by searching for sampling events in the `Events` menu:

Events

Date Range

Start Date	and	End Date

Refresh

Showing **1-1** of **1** item.

#	Type	Description	Concern	Alias	Date of Event	
	sampling			60		
1	oeev:Sampling	Sampling ligulated leaf 8 of plant 60 for ABA analysis	m3p:/arch /2017/lc17000060	0060/DZ_PG_27/ZM4354/WD/Veg_1 /01_60/8/ARCH2017-03-30	2017-05-16T12:00:00+01:00 10:51	

http://www.phenome-fppn.fr/id/event/b020e89f-3ac7-4eee-baa0-70c3cbec30ce

Add annotation Browse Triplestore

**Type**	oeev:Sampling
**Description**	Sampling ligulated leaf 8 of plant 60 for ABA analysis
**Author**	http://www.phenome-fppn.fr/id/agent/lcabrera
**Date of Event**	2017-05-16T12:00:00+01:00 10:51
**Concern**	m3p:/arch/2017/lc17000060
**Alias**	0060/DZ_PG_27/ZM4354/WD/Veg_1/01_60/8/ARCH2017-03-30
