# Supplementary material for: Dealing with multi‐source and multi‐scale information in plant phenomics: the ontology‐driven Phenotyping Hybrid Information System
Source: New Phytol. 2018 Aug 28;221(1):588–601. doi: 10.1111/nph.15385 (PMC6585972; doi:10.1111/nph.15385)

Object tracking

The Experimental Organization menu contains a `Scientific Object Tracking tool`.

Searching filters allow selecting for scientfic objects such as plants, plots or organs or keywords. For instance, the search of **arch** in the `Uri` box and **262** in the `Alias` box results in three different outputs:

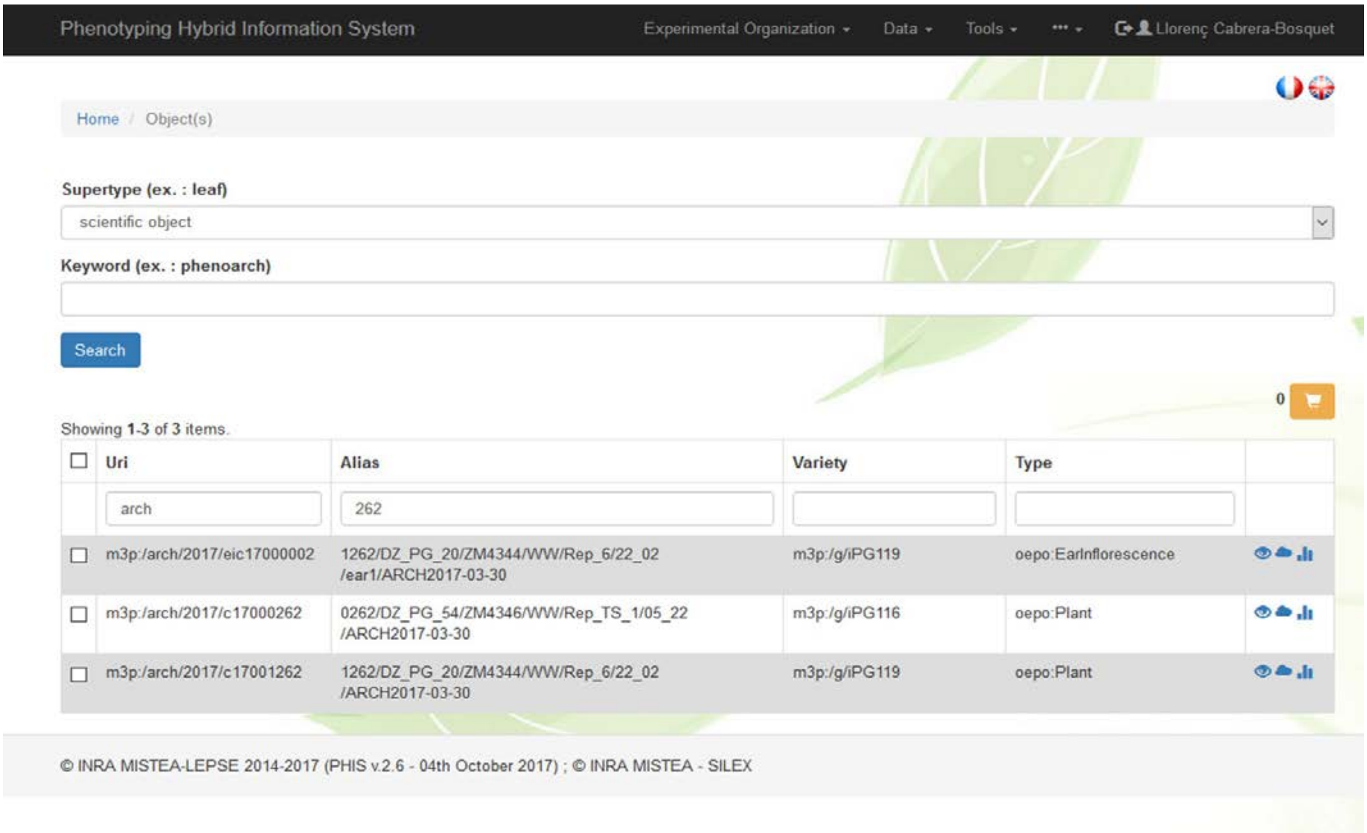

Wheras the first result correponds to an ear inflorescence (Type = **oepo:EarInflorescence**) the other results correspond to a Leaf (Type = **oepo:Leaf**). Objects (either plants or leaves) have their own URI. The filtered objects can be individually selected by clicking on left boxes and are automatically placed in a basket for further analyses.

Selected objects can be visualised by clicking on the `shopping car` icon.

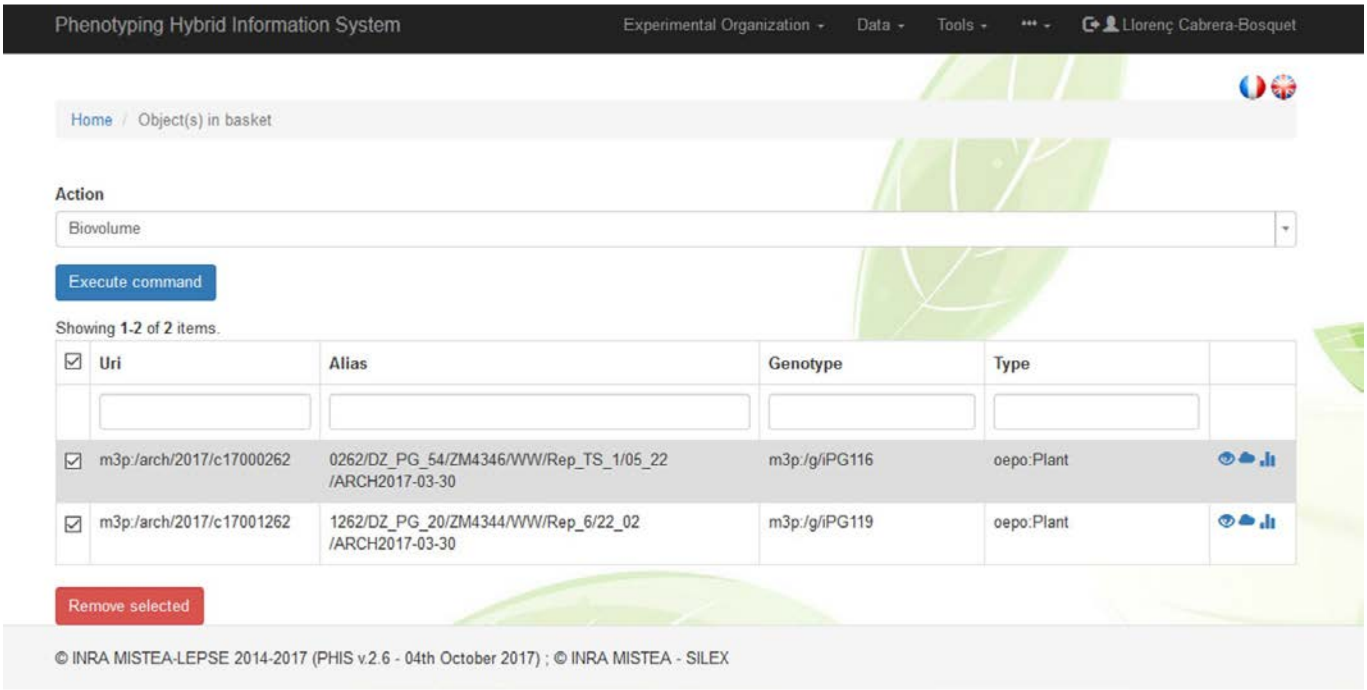

Once the objects are in the shopping car (i.e. plants *m3p:/arch/2017/c17000262* and *m3p:/arch/2017/c17001262*), different analyses ca be performed:

Trait comparison

This feature allows different elaborated variables such as biovolume, leaf area or plant height to be compared between selected objects:

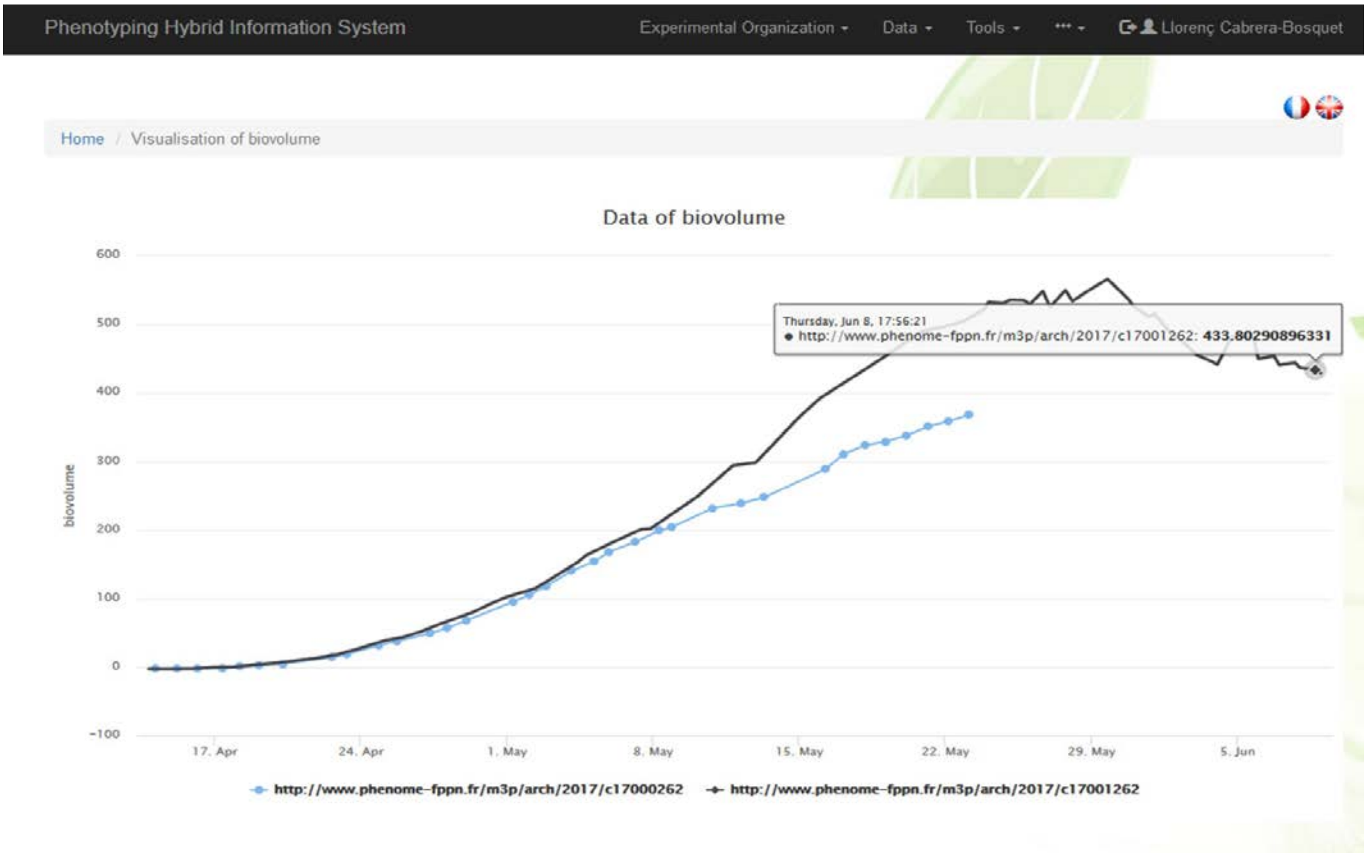

Object inspection

The different icons on the right side of each selected object allow different actions:

- : Allows browsing the object properties including annotations and events
- : Allows visualisation of environmental conditions sensed by each object
- : Allows visualisation of different time courses of traits

RDF details

Detailed information of object characteristics and annotations or events is displayed in the form of RDF triples (subject - predicate - object):

- `http://www.phenome-fppn.fr/m3p/arch/2017/c17000262` `rdf:type` `oepo:Plant` represents plant 262 is of type plant
- `http://www.phenome-fppn.fr/m3p/arch/2017/c17000262` `oepo:hasRepetition` `1` represents plant 262 has rep 1 `http://www.phenome-fppn.fr/m3p/arch/2017/c17000262`
- `oepo:participatesIn` `m3p:/ARCH2017-03-30` represents plant 262 participates in experiment ARCH2017-03-30

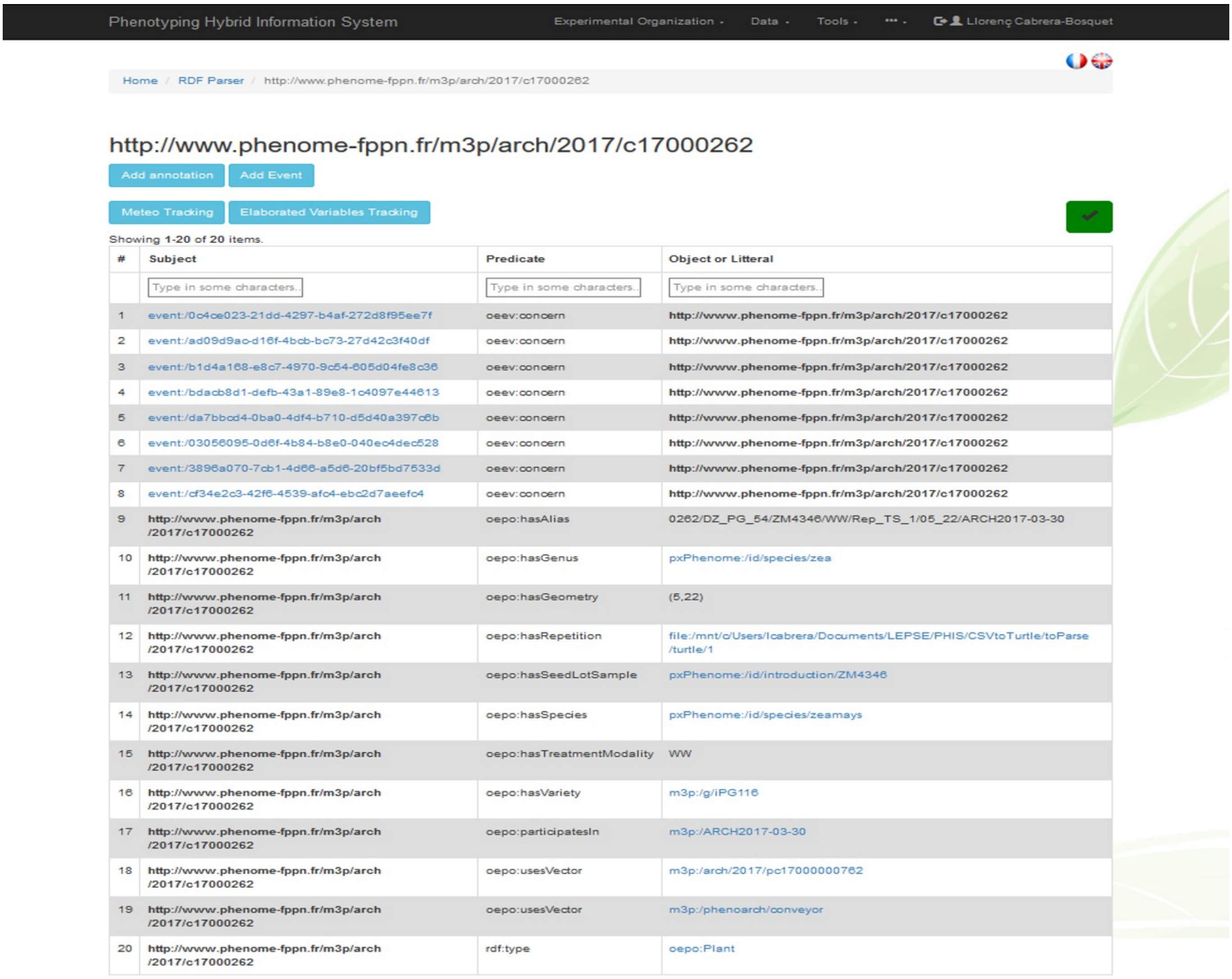

#	Subject	Predicate	Object or Litteral
	Type in some characters...	Type in some characters...	Type in some characters...
1	event:/0c4ce023-21dd-4297-b4af-272d8f95ee7f	oeev:concern	http://www.phenome-fppn.fr/m3p/arch/2017/c17000262
2	event:/ad09d9ac-d16f-4bcb-bc73-27d42c3f40df	oeev:concern	http://www.phenome-fppn.fr/m3p/arch/2017/c17000262
3	event:/b1d4a168-e8c7-4970-9c54-605d04fe8c36	oeev:concern	http://www.phenome-fppn.fr/m3p/arch/2017/c17000262
4	event:/bdacb8d1-defb-43a1-89e8-1c4097e44613	oeev:concern	http://www.phenome-fppn.fr/m3p/arch/2017/c17000262
5	event:/da7bbcd4-0ba0-4df4-b710-d5d40a397c6b	oeev:concern	http://www.phenome-fppn.fr/m3p/arch/2017/c17000262
6	event:/03056095-0d6f-4b84-b8e0-040ec4dec528	oeev:concern	http://www.phenome-fppn.fr/m3p/arch/2017/c17000262
7	event:/3896a070-7cb1-4d66-a5d6-20bf5bd7533d	oeev:concern	http://www.phenome-fppn.fr/m3p/arch/2017/c17000262
8	event:/cf34e2c3-42f6-4539-afc4-ebc2d7aeefc4	oeev:concern	http://www.phenome-fppn.fr/m3p/arch/2017/c17000262
9	http://www.phenome-fppn.fr/m3p/arch /2017/c17000262	oepo:hasAlias	0262/DZ_PG_54/ZM4346/WW/Rep_TS_1/05_22/ARCH2017-03-30
10	http://www.phenome-fppn.fr/m3p/arch /2017/c17000262	oepo:hasGenus	pxPhenome:/id/species/zea
11	http://www.phenome-fppn.fr/m3p/arch /2017/c17000262	oepo:hasGeometry	(5,22)
12	http://www.phenome-fppn.fr/m3p/arch /2017/c17000262	oepo:hasRepetition	file:/mnt/c/Users/lcabrera/Documents/LEPSE/PHIS/CSVtoTurtle/toParse /turtle/1
13	http://www.phenome-fppn.fr/m3p/arch /2017/c17000262	oepo:hasSeedLotSample	pxPhenome:/id/introduction/ZM4346
14	http://www.phenome-fppn.fr/m3p/arch /2017/c17000262	oepo:hasSpecies	pxPhenome:/id/species/zeamays
15	http://www.phenome-fppn.fr/m3p/arch /2017/c17000262	oepo:hasTreatmentModality	WW
16	http://www.phenome-fppn.fr/m3p/arch /2017/c17000262	oepo:hasVariety	m3p:/g/iPG116
17	http://www.phenome-fppn.fr/m3p/arch /2017/c17000262	oepo:participatesIn	m3p:/ARCH2017-03-30
18	http://www.phenome-fppn.fr/m3p/arch /2017/c17000262	oepo:usesVector	m3p:/arch/2017/pc17000000762
19	http://www.phenome-fppn.fr/m3p/arch /2017/c17000262	oepo:usesVector	m3p:/phenoarch/conveyor
20	http://www.phenome-fppn.fr/m3p/arch /2017/c17000262	rdf:type	oepo:Plant

Environmental conditions

This feature allows to follow the environmental conditions sesnsed by a given plant. This is of particular interest when plants are transfered between installations or compartments with different environmental

conditions. In the exemple presented here, `plant 262` is sequentially monitored in two installations over 50 days. The experiment takes place in `Installation 1` (**http://www.phenome-fppn.fr/m3p/phenoarch**) for daily measurements of biovolume and transpiration from April 6 to May 19, and then the plant is moved to `Installation 2` (**http://www.phenome-fppn.fr/m3p/phenodyn**) for ten days where was subjected to a thermal stress (May 19 to May 29) before being harvested (May 29). As shown in the graph below, `air temperature` sensed by the plant largely differ between the two installations, so a proper tracking tool is essential.

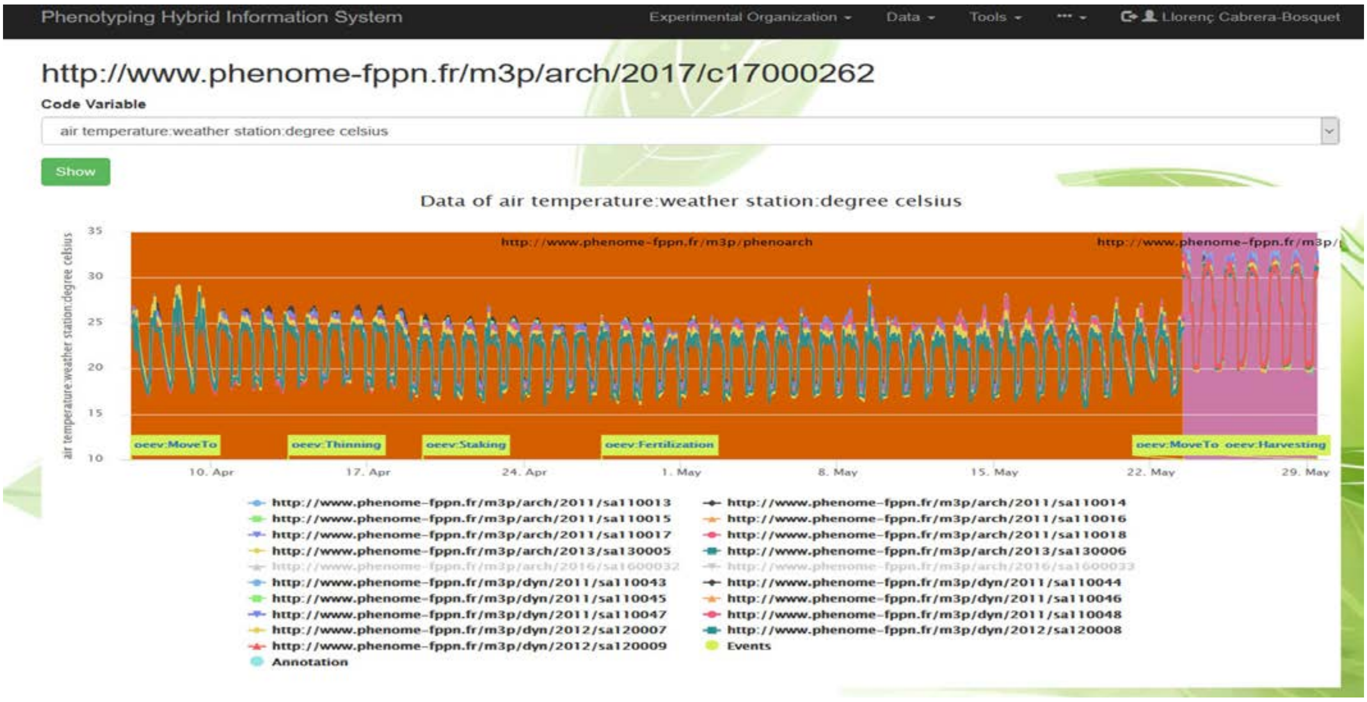

Events displayed in green boxes along the time axis can be explored by clicking on. For instance, clicking on the box displaying `oeev:fertilization` allows exploring all the information related to such event.

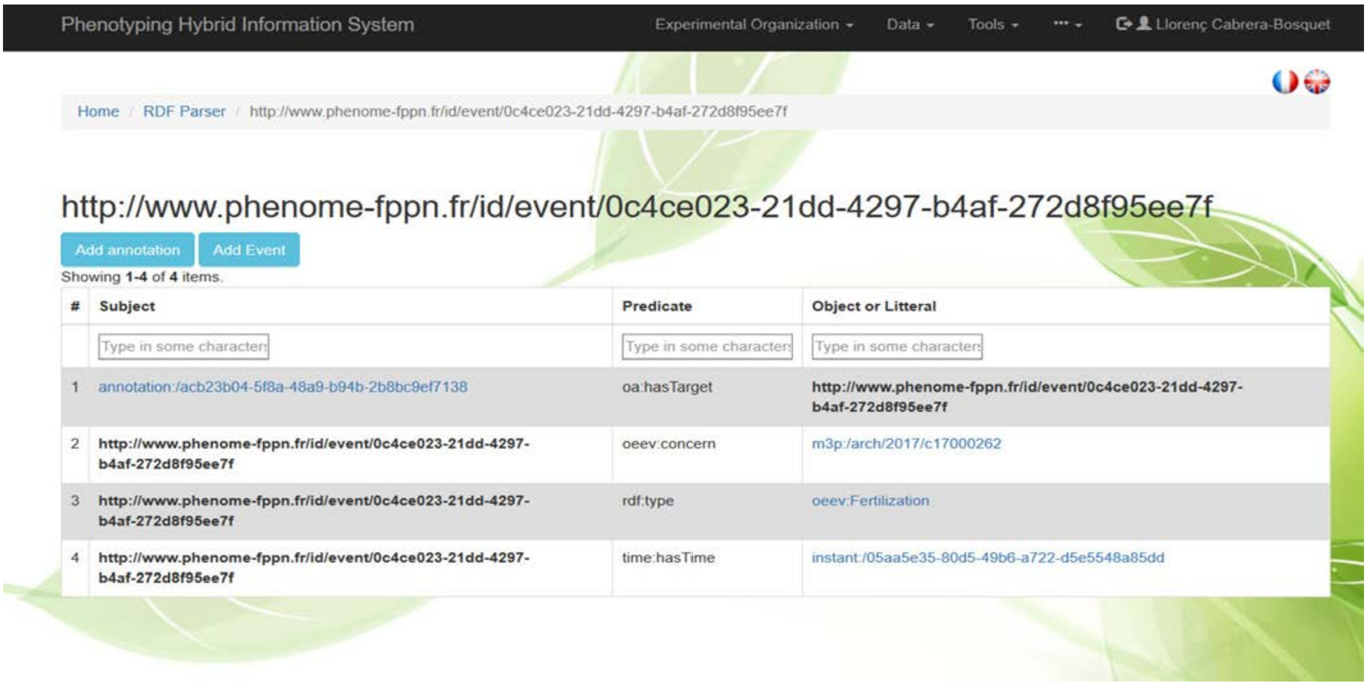

Time courses of variables

For instance `biovolume` over time can be displayed together with the annotations and events (e.g. sowing, harvesting) that occured to a given plant. Orange and pink panels represent the installations **phenoarch** and **phenodyn**, respectively:

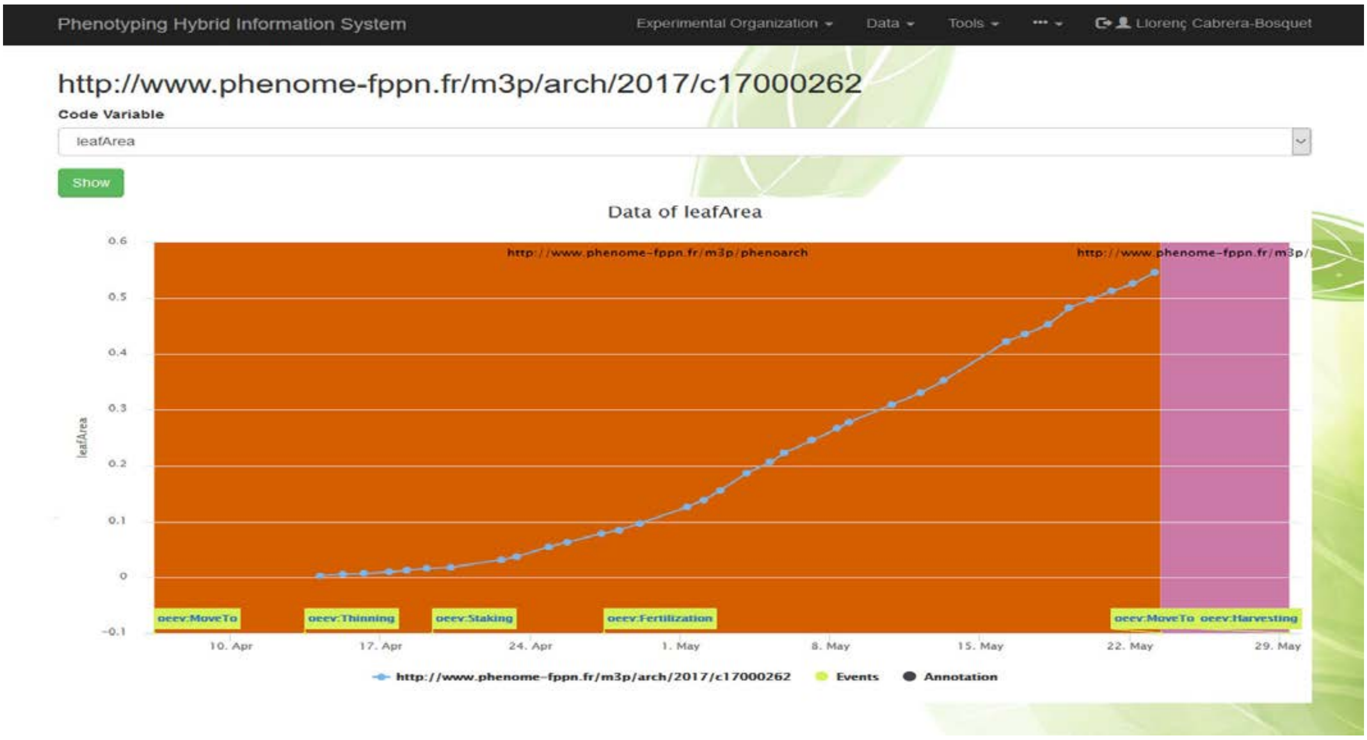

Events displayed in green boxes along the time axis can be explored by clicking on. For instance, clicking on the box displaying `oeev:Harvesting` allows exploring all the information related to such event:

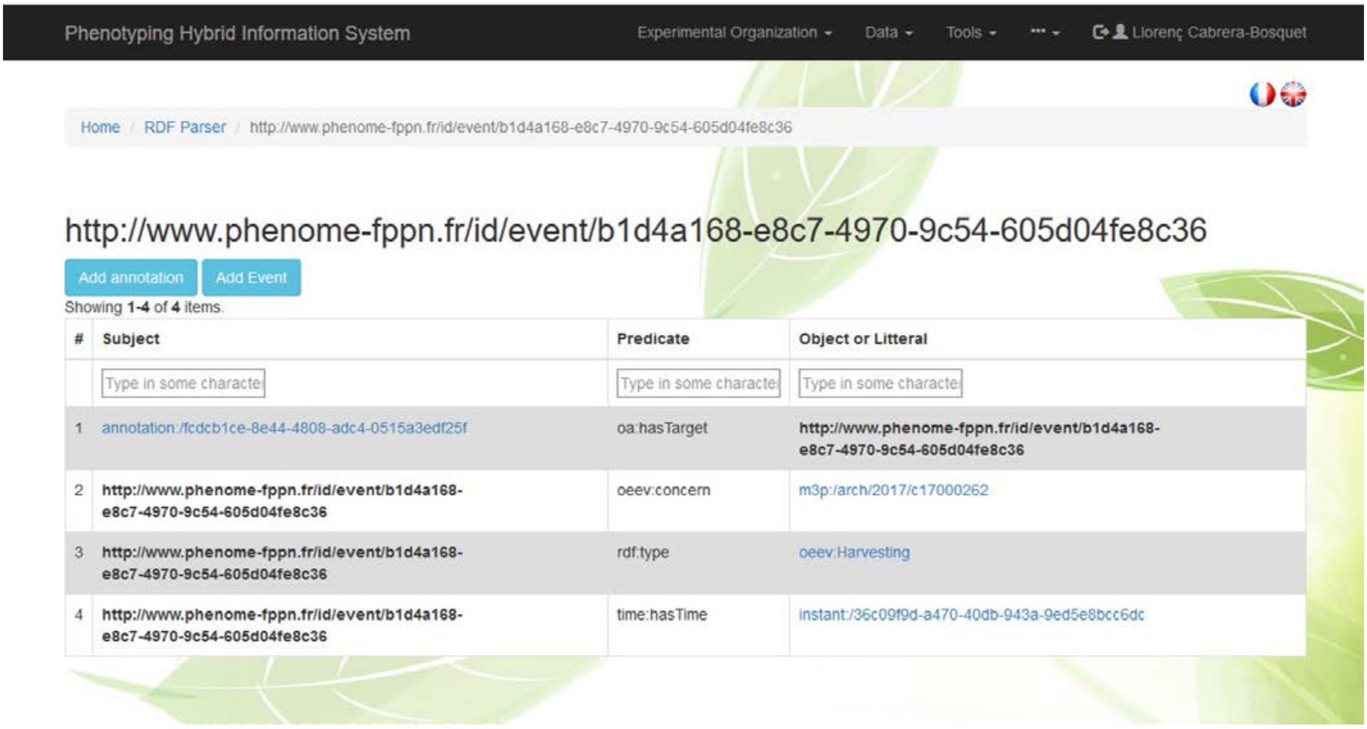

Supplement: Supplementary file 3 — Notes S3 Object tracking menu of PHIS web user interface. [file NPH-221-588-s003.pdf]
