# Supplementary material for: Dealing with multi‐source and multi‐scale information in plant phenomics: the ontology‐driven Phenotyping Hybrid Information System
Source: New Phytol. 2018 Aug 28;221(1):588–601. doi: 10.1111/nph.15385 (PMC6585972; doi:10.1111/nph.15385)
Supplement: Supplementary file 4 — Notes S4 Data menu of PHIS web user interface. [file NPH-221-588-s004.pdf]

Data menu

The Data menu contains tools for advanced data querying, visualization features for displaying images and time courses of phenotypic and environmental variables and scientific computing features including data analysis tools and integration of workflows.

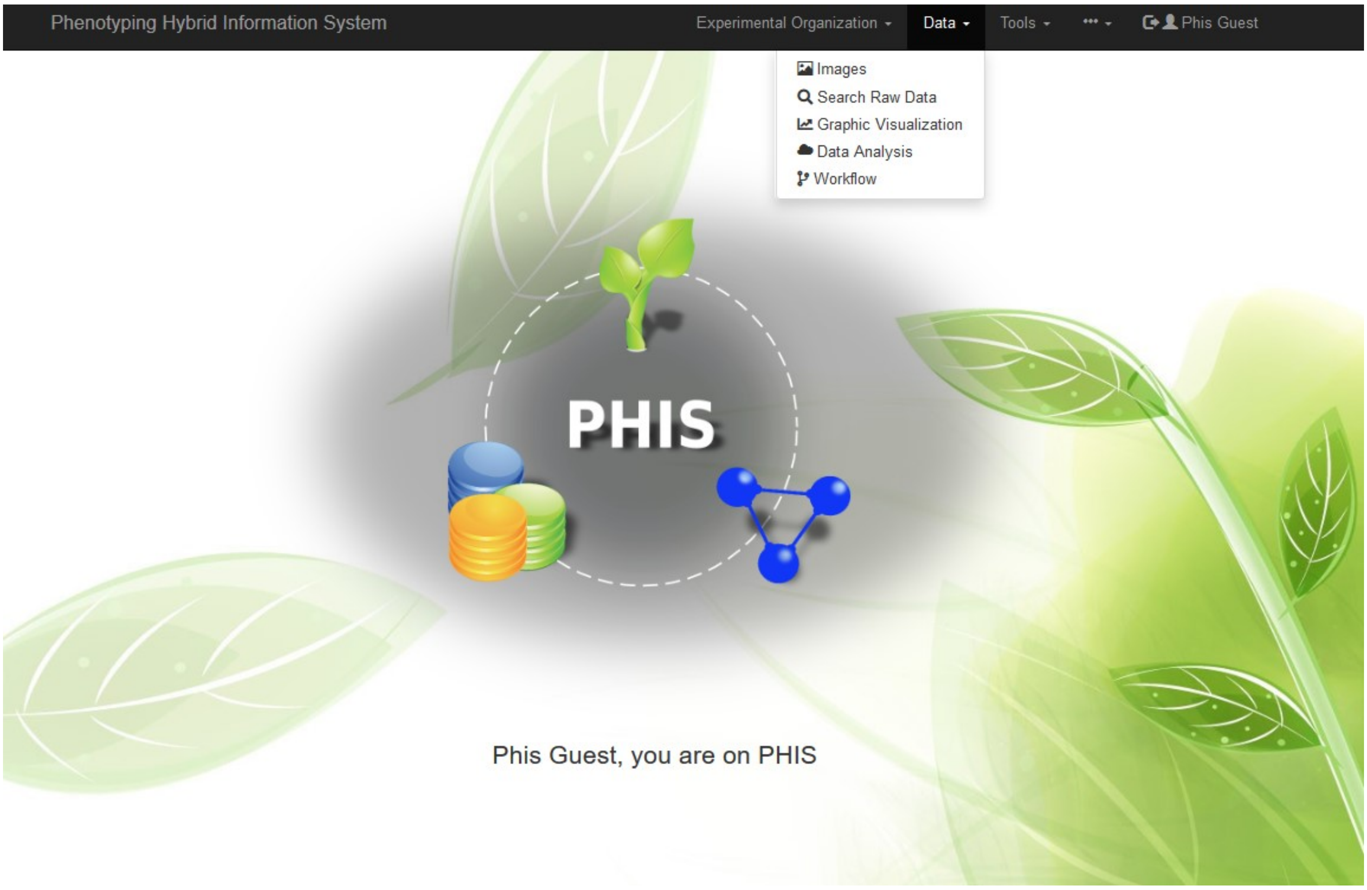

Images

Raw images (e.g. RGB, hemispherical,...), thumbnails and analysed images (e.g. segmented images) stored on the distributed storage system **iRODS**, can be accessed using a filtering form based on `URIs`, `Experiments`, `Genotypes` and `Dates`

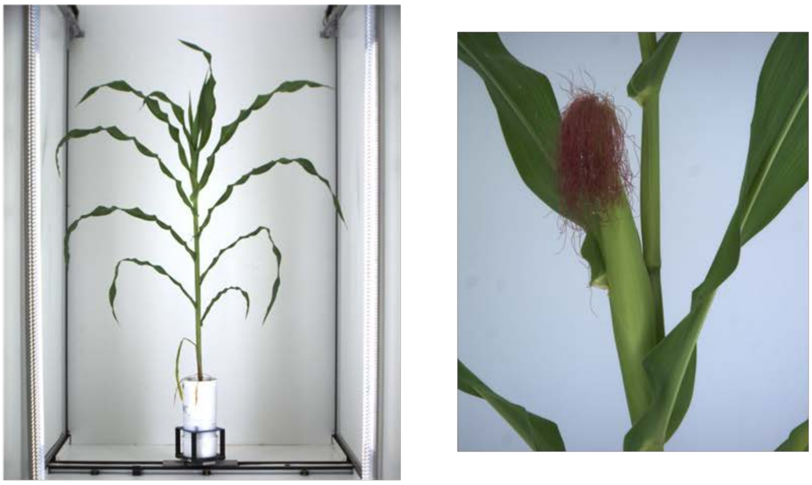

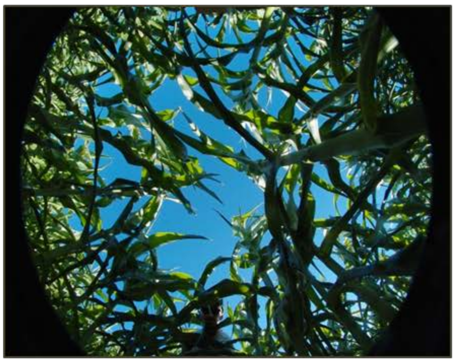

Plant and ear RGB images (greenhouse) ***Hemispherical image (field)***

Images can be displayed together different fields including: `uri`, `experiment`, `genotype`,`scenario`,`repetition`,`seedlot`, `camera angle`,...

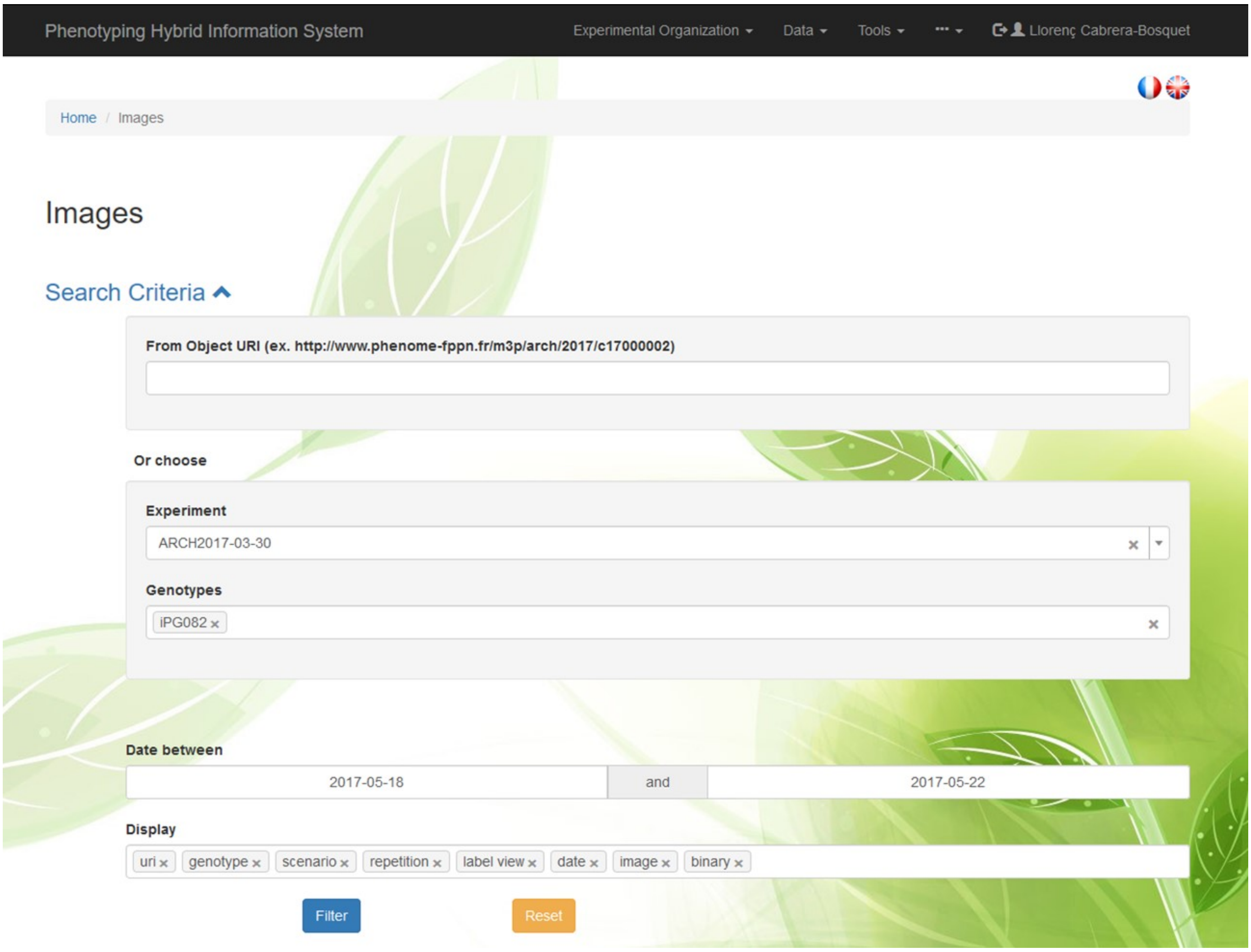

Results of image analysis can be displayed by chossing the option `binary` in the filter `Fields to display`

Images can be displayed and scrolled by clicking in the eye icon.

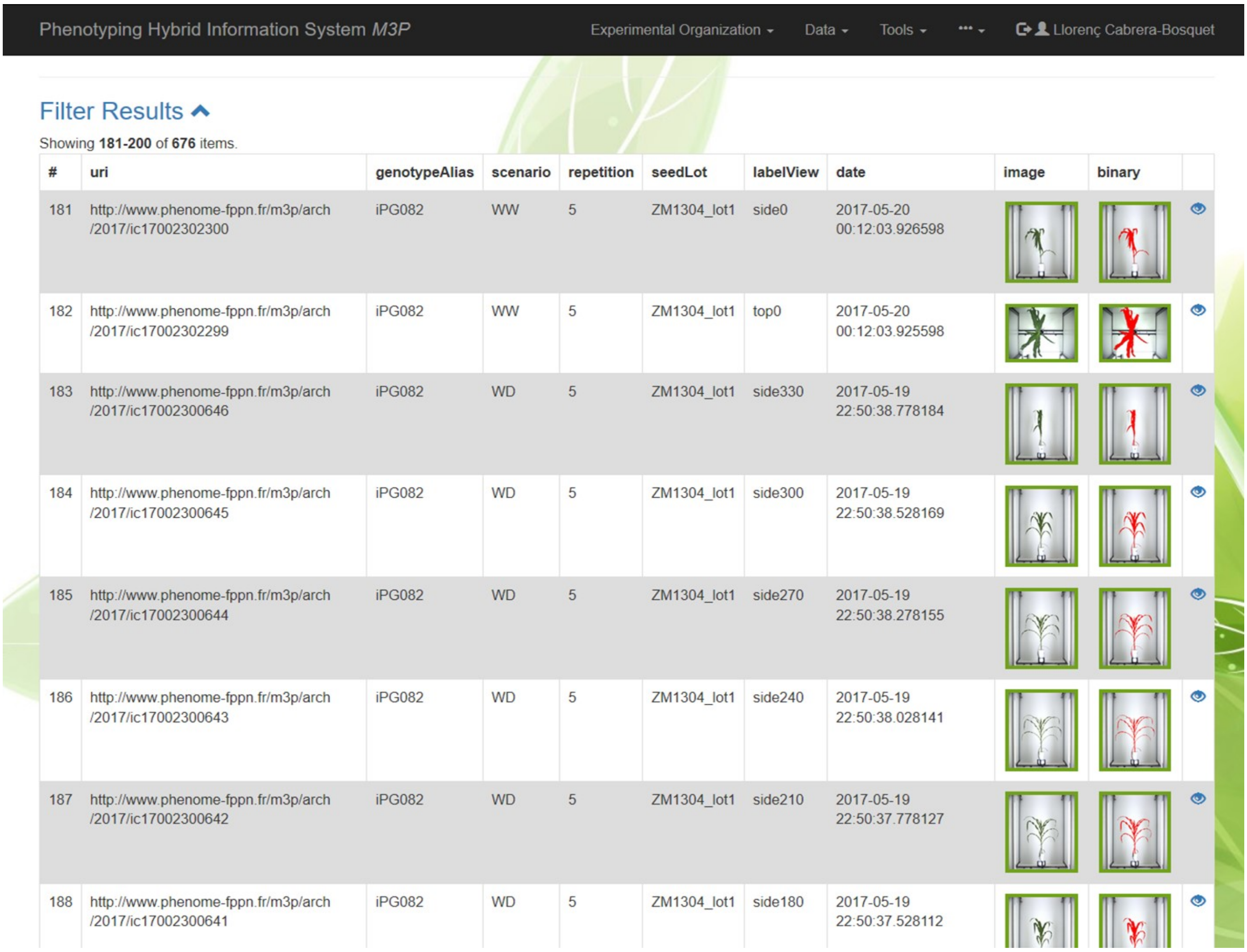

#	uri	genotypeAlias	scenario	repetition	seedLot	labelView	date	image	binary
181	http://www.phenome-fppn.fr/m3p/arch/2017/ic17002302300	iPG082	WW	5	ZM1304_lot1	side0	2017-05-20 00:12:03.926598		
182	http://www.phenome-fppn.fr/m3p/arch/2017/ic17002302299	iPG082	WW	5	ZM1304_lot1	top0	2017-05-20 00:12:03.925598		
183	http://www.phenome-fppn.fr/m3p/arch/2017/ic17002300646	iPG082	WD	5	ZM1304_lot1	side330	2017-05-19 22:50:38.778184		
184	http://www.phenome-fppn.fr/m3p/arch/2017/ic17002300645	iPG082	WD	5	ZM1304_lot1	side300	2017-05-19 22:50:38.528169		
185	http://www.phenome-fppn.fr/m3p/arch/2017/ic17002300644	iPG082	WD	5	ZM1304_lot1	side270	2017-05-19 22:50:38.278155		
186	http://www.phenome-fppn.fr/m3p/arch/2017/ic17002300643	iPG082	WD	5	ZM1304_lot1	side240	2017-05-19 22:50:38.028141		
187	http://www.phenome-fppn.fr/m3p/arch/2017/ic17002300642	iPG082	WD	5	ZM1304_lot1	side210	2017-05-19 22:50:37.778127		
188	http://www.phenome-fppn.fr/m3p/arch/2017/ic17002300641	iPG082	WD	5	ZM1304_lot1	side180	2017-05-19 22:50:37.528112		

Search raw data

Advanced data querying allows rapid and efficient visualisation of images and monitoring variables guided by filtering tools based on semantics and rules such as mathematical operators.

For instance, in the exemple shown here, the query for Ground cover measurements ranging between 0.7 and 0.8 gives the following results:

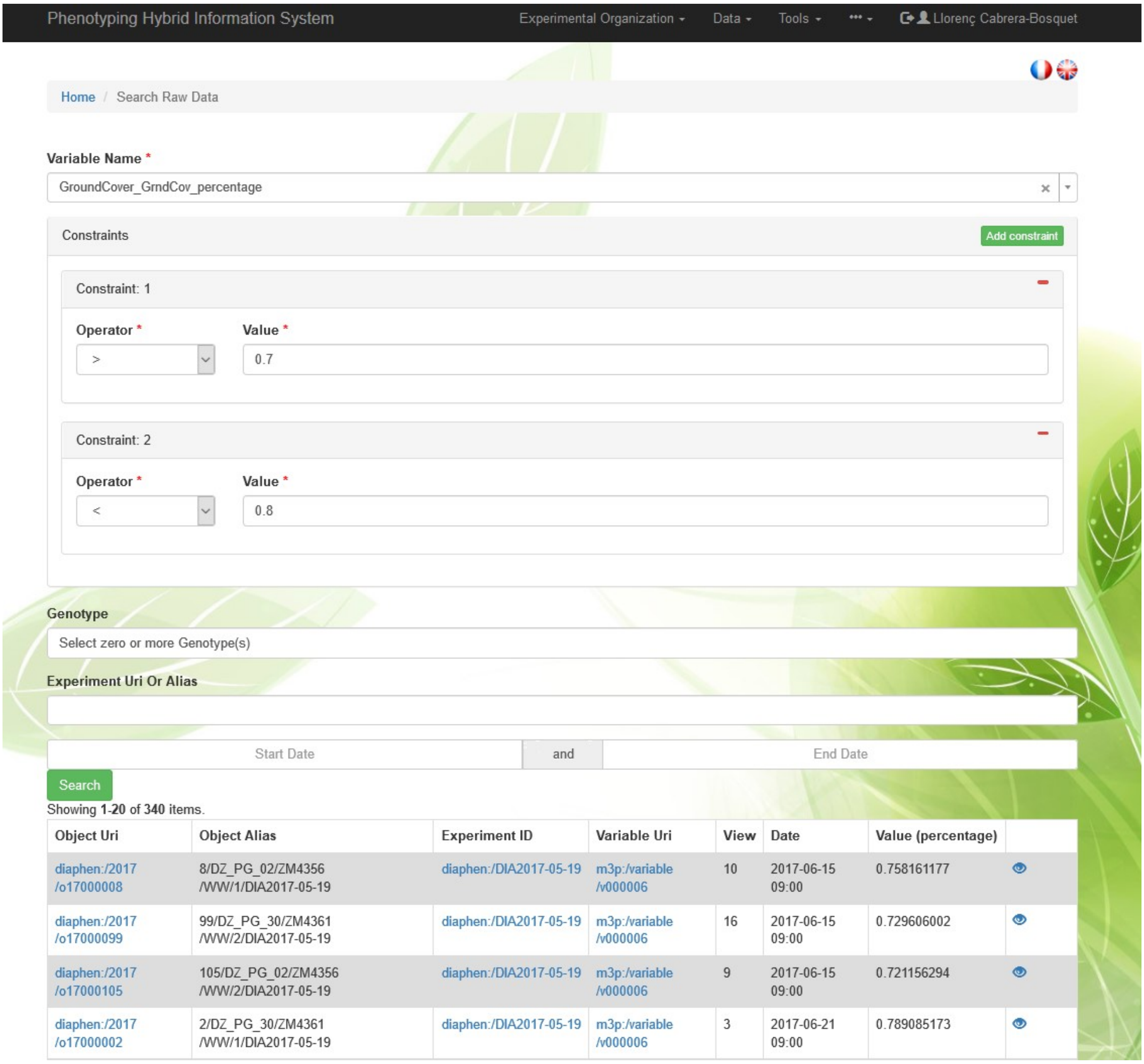

Each of the results can be explored, and extra information is provided:

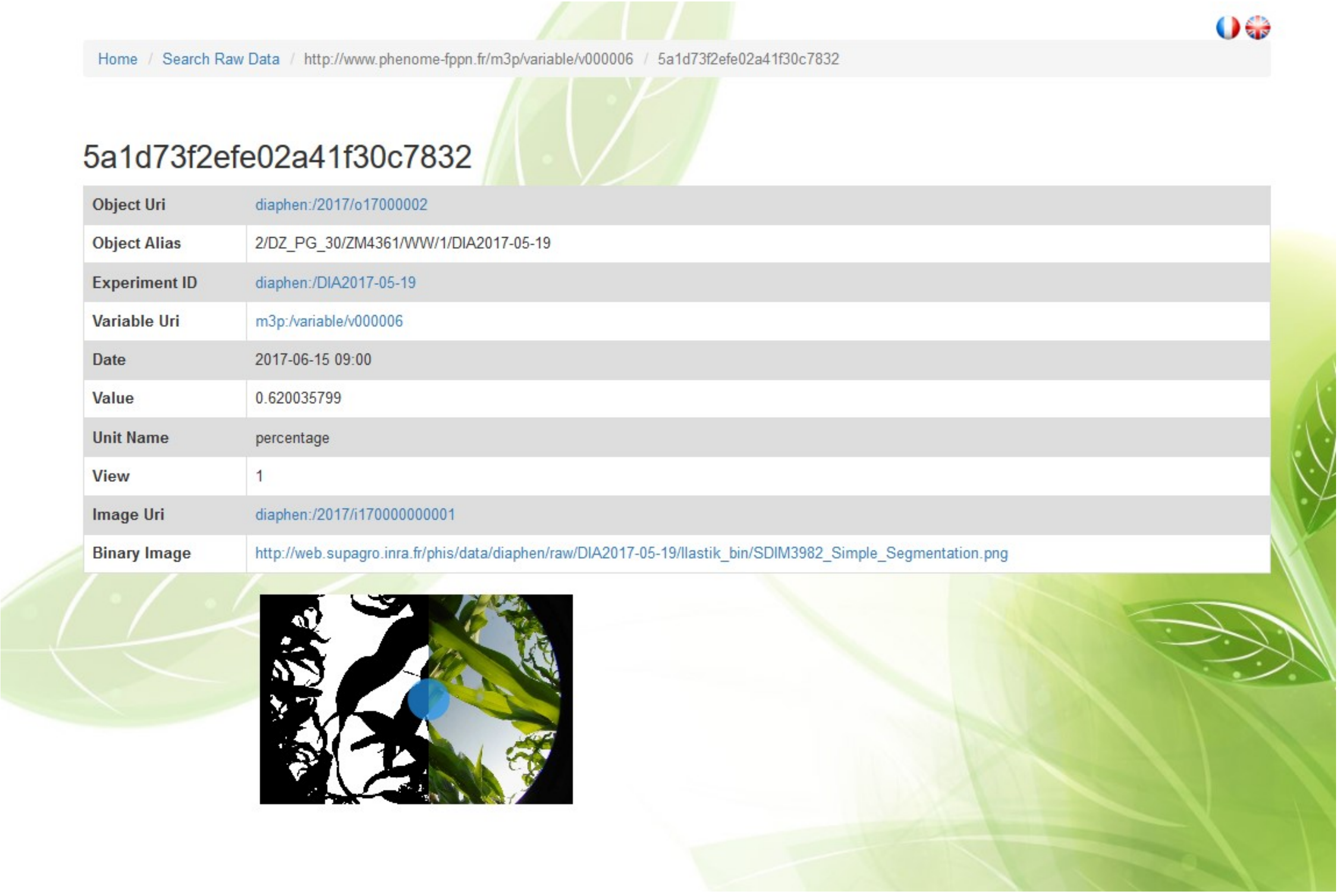

Object Uri	diaphen:/2017/o17000002
Object Alias	2/DZ_PG_30/ZM4361/WW/1/DIA2017-05-19
Experiment ID	diaphen:/DIA2017-05-19
Variable Uri	m3p:/variable/v000006
Date	2017-06-15 09:00
Value	0.620035799
Unit Name	percentage
View	1
Image Uri	diaphen:/2017/i170000000001
Binary Image	http://web.supagro.inra.fr/phis/data/diaphen/raw/DIA2017-05-19/llastik_bin/SDIM3982_Simple_Segmentation.png

An image comparison slider tool allows comparing raw and segmented images:

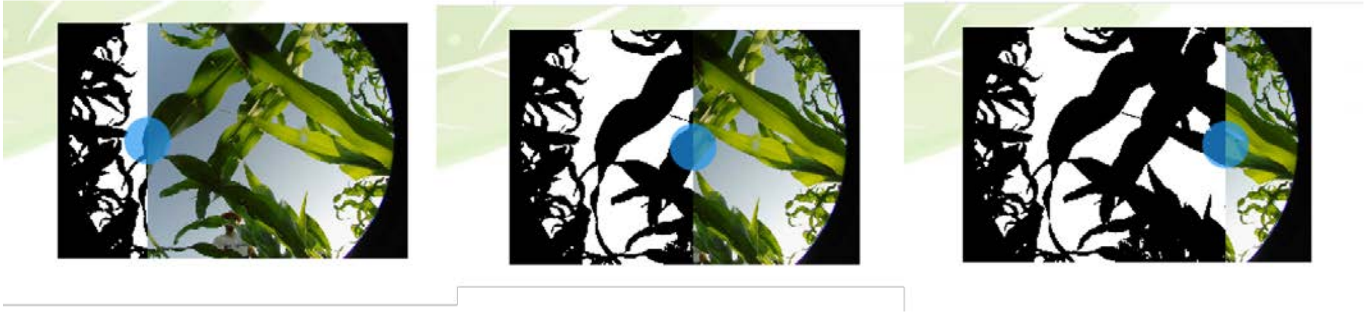

Graphic visualisation

The graphic visualisation menu contains features for displaying images, dynamic graphs of static or time courses of phenotypic and environmental variables, which are automatically adapted to the particular experimental settings and variables, such as greenhouse or field. For instance, the users may request a dynamic visualization of image analyses and watering results based on different filtering options (image angles, genotypes, plants, treatments). Such interactive figures allow exploring dynamic variables over time (e.g. plant area or water-use).

Graphs can be zoomed into a particular time window, and clicking on a data point automatically displays the images associated to this point together with the associated annotations. Raw images, segmented images and metadata can be displayed in both field and greenhouse experiments. The interaction with the knowledge layer allows projecting variables using GPS coordinates associated to plants or plots in field experiments and linking data with environmental sensor outputs.

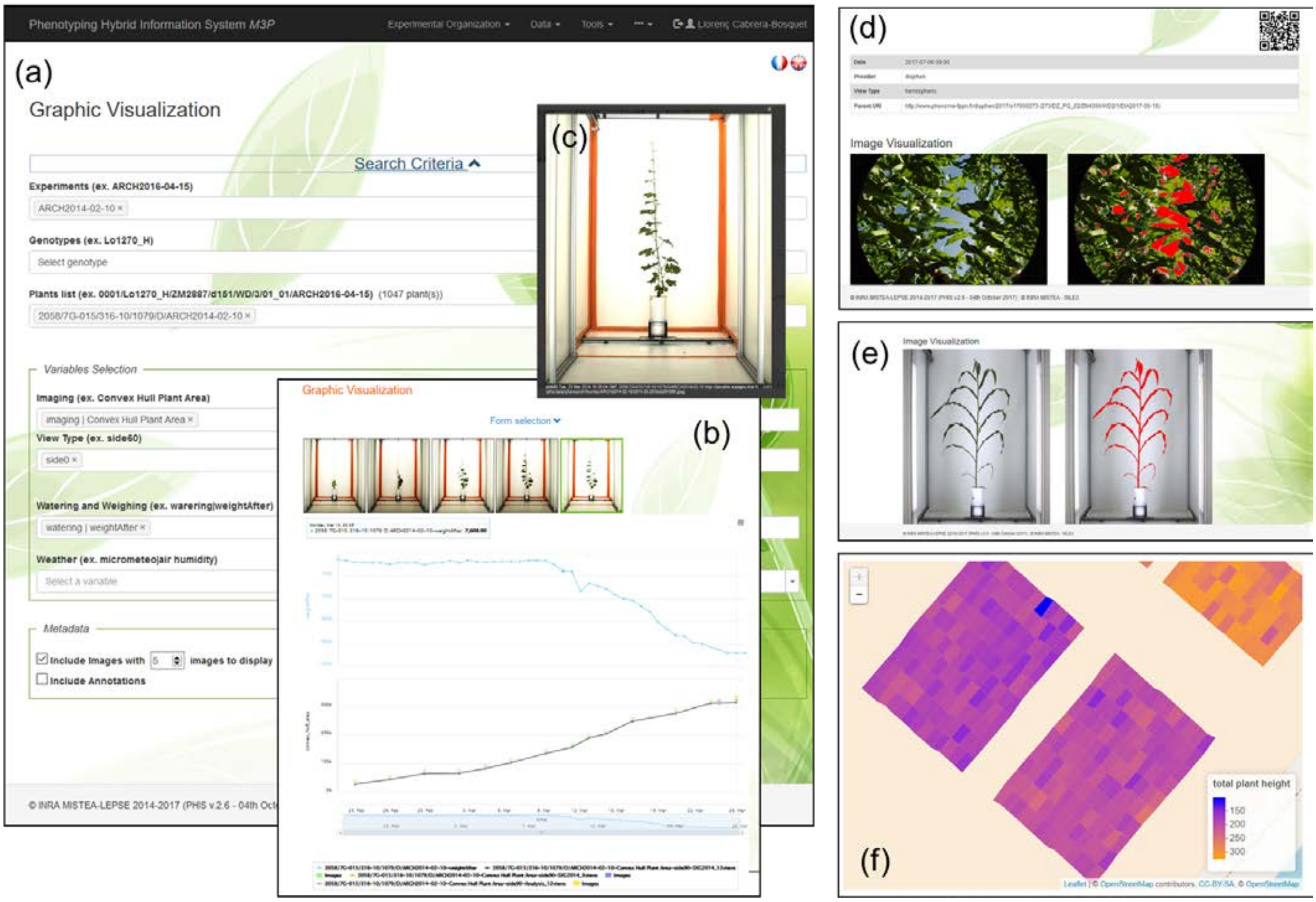

Graphs can be exported into different formats (i.e. PDF, JPG, PNG, SVG).

Data Analysis

PHIS includes extensible scientific computing modules based on R packages for calculating elaborated variables and generating experimental summaries and reports. Automatic reporting is based on data query through the interface and R integration in text processing (**R Markdown**). Different R (**R Core Team, 2015**) packages ('*dplyr*', '*mongolite*', '*RPostgreSQL*', '*jsonlite*', '*httr*', '*urltools*') are also used to link databases and the Web User Interface. Basic reports include daily, weekly or final overviews of experiments, with standard statistics and graphic visualization of averaged queried traits. Extended reports may include specific calculation of traits and environmental conditions associated to a given plant or genotype. In particular, new variables can be calculated for a given experiment based on the joint use of phenotypic and environmental data together with associated metadata to plants and traits (e.g. events, methods).

Six analysis are available:

- Daily Greenhouse Report
- Daily Field Report
- Environment Field Report
- Environment Greenhouse Report
- Global Greenhouse Report
- Thermal time Calculation Report

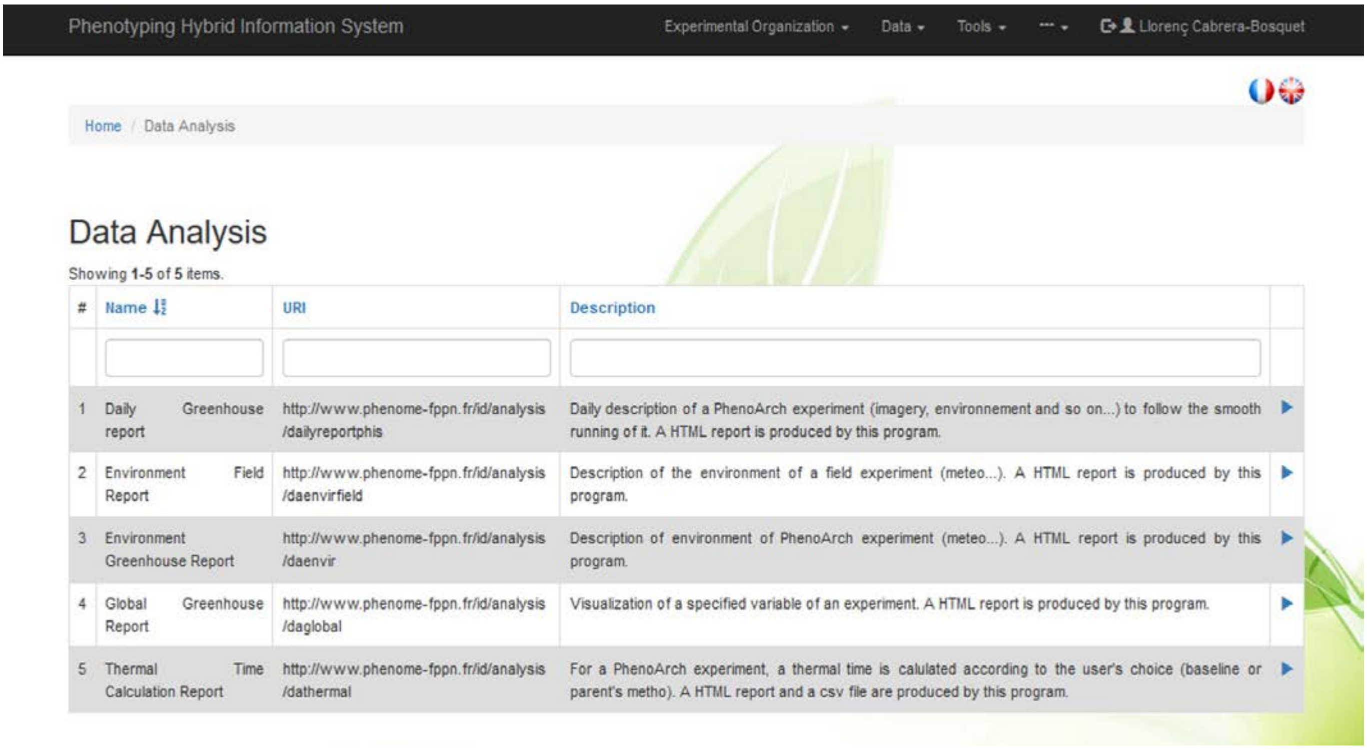

For instance, a `Thermal Time Calculation Report` allows calculating the progression of thermal time for each `experiment`, taking into account diffrent parameters like the `species`, the `method` used (e.g. baseline temperature or Parent *et al.* method[1]) the `event` to start computation (e.g. sowing, emergence,thinning) or a given calendar date, and `temperature data` (air or leaf temperatures)

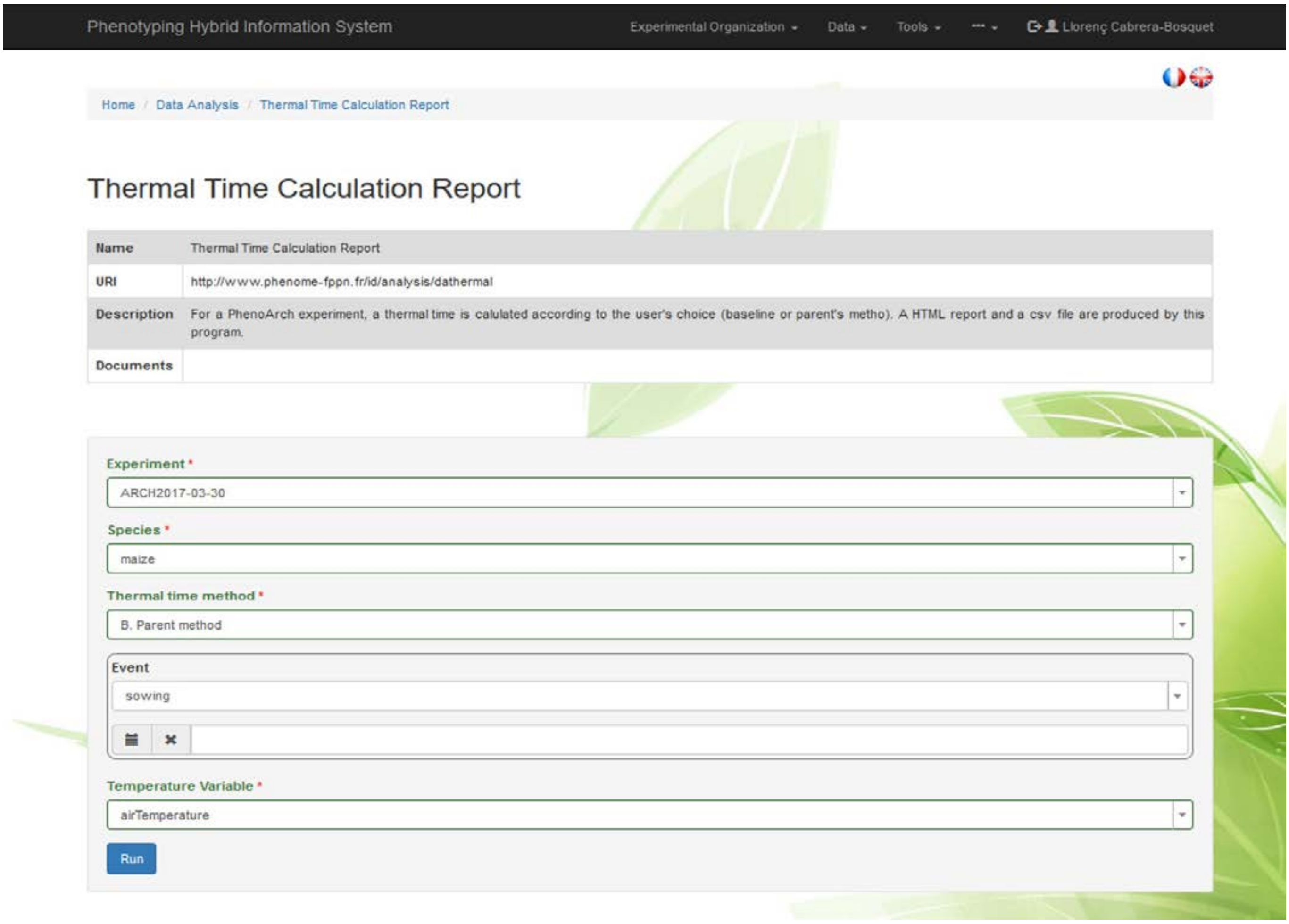

An HTML report of this analysis and a .csv file containing the calculated thermal time is provided at the end of the analysis:

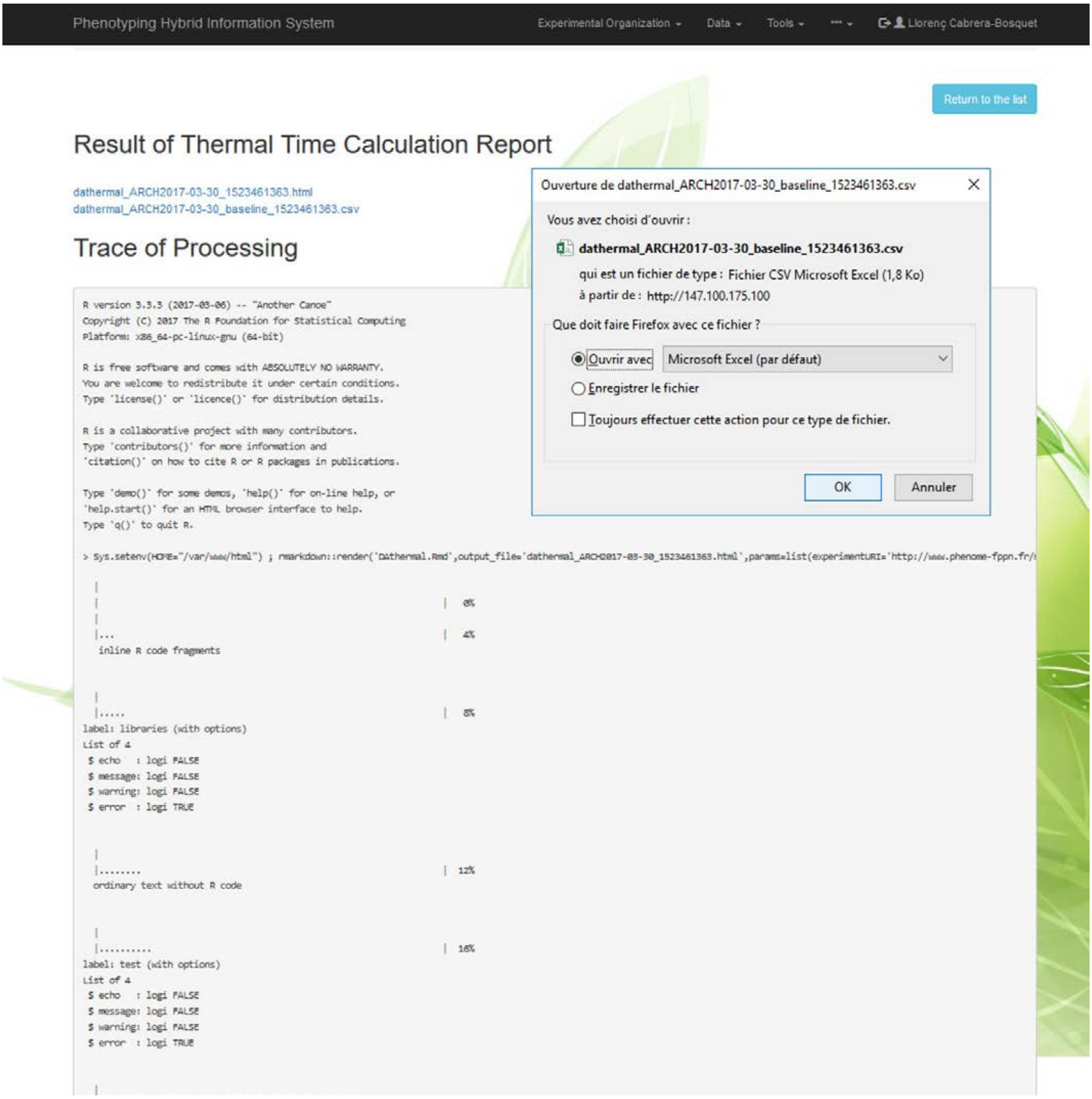

Workflows

Workflow feature icluded in PHIS enables computational analysis and workflows through the scientific platform **Galaxy**.

The first step consists in getting connection to Galaxy following these instructions:

The first step is to open Workflows and click Connect to Galaxy

1

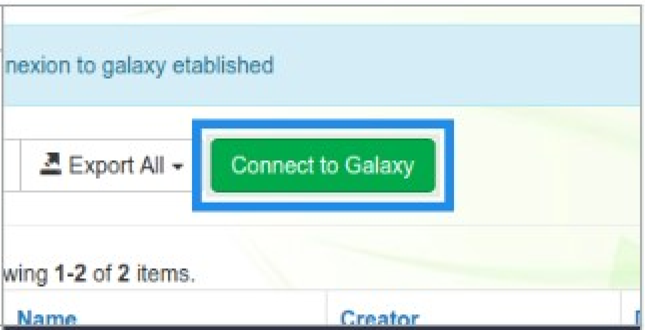

click Authentication

2

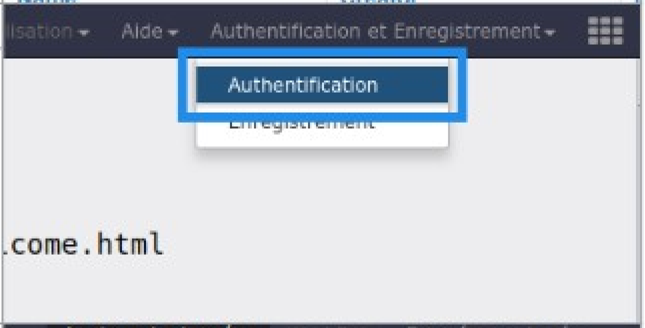

Use the same username and password used in PHIS
Click Login

3

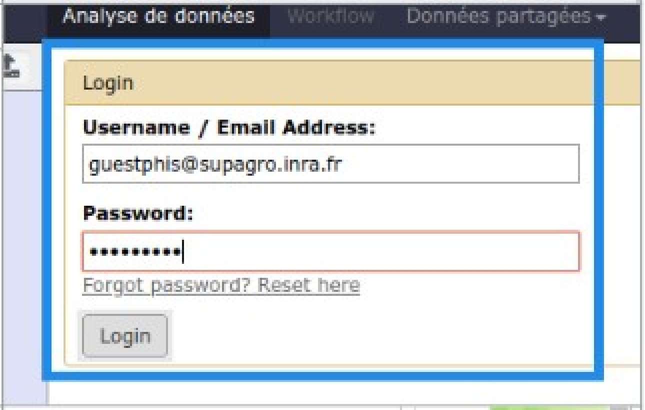

Go back to PHIS and Run the choosed workflow

4

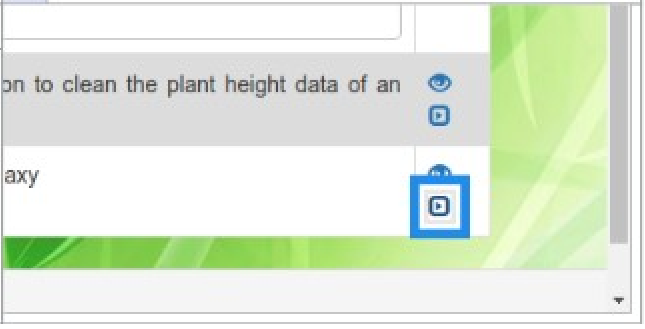

Then workflows can be executed. As an exemple, the simple Workflow `TEST Echo workflow` is shown, containing different attributes and input parameters:

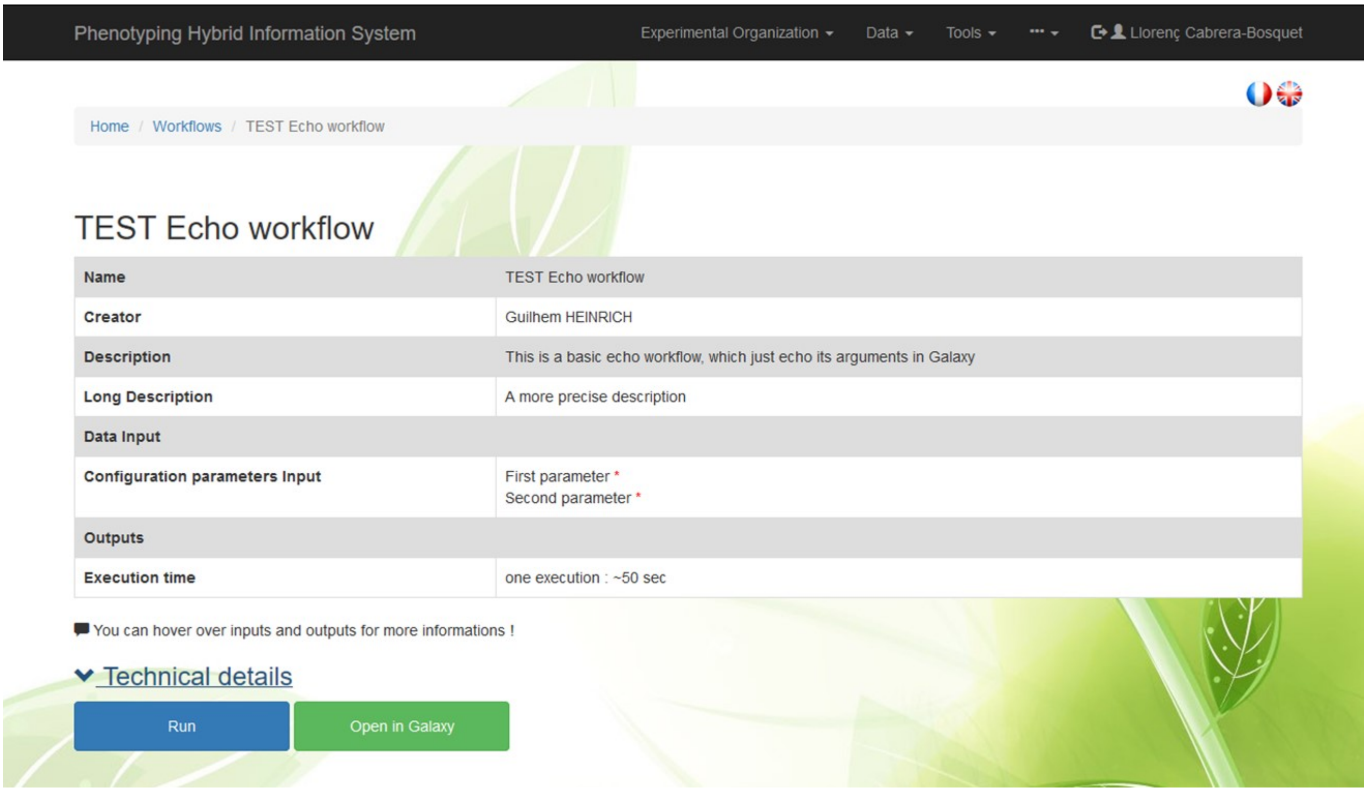

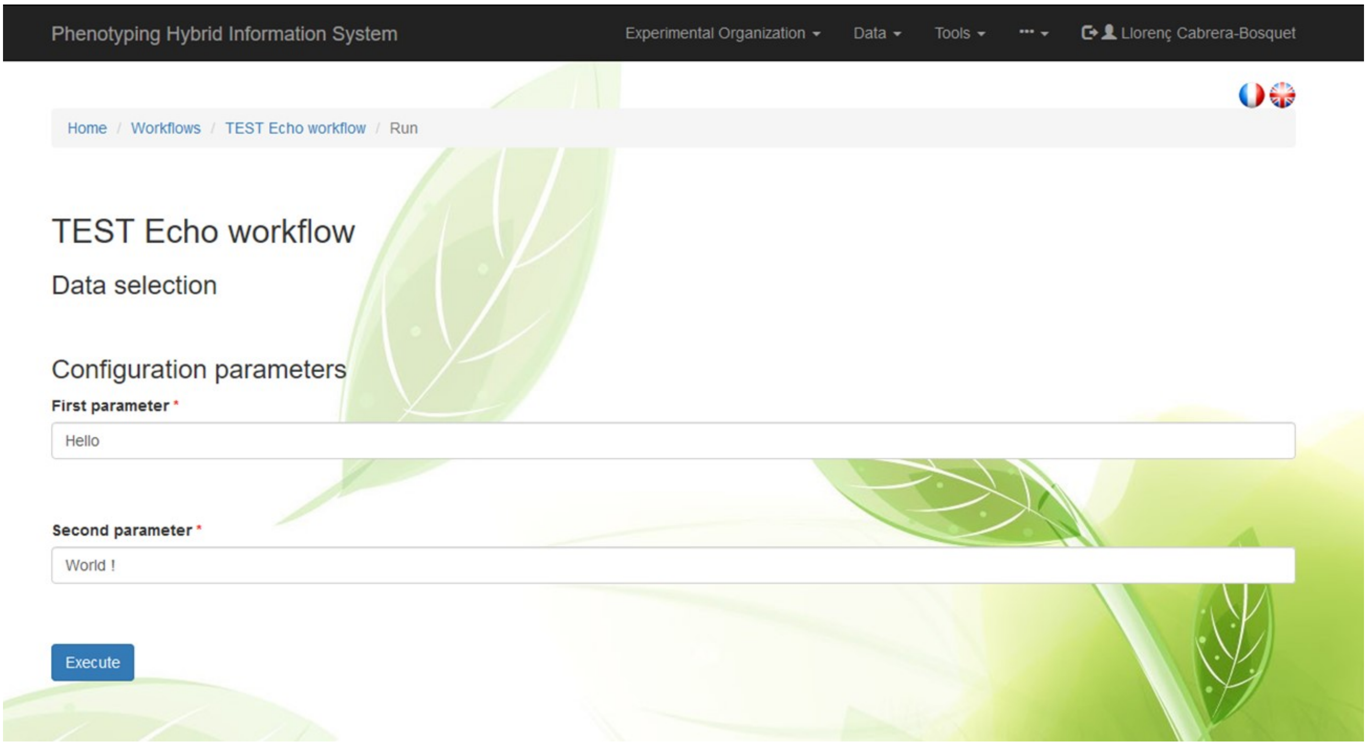

Current tasks can be visualised by clicking on the `Current tasks` button:

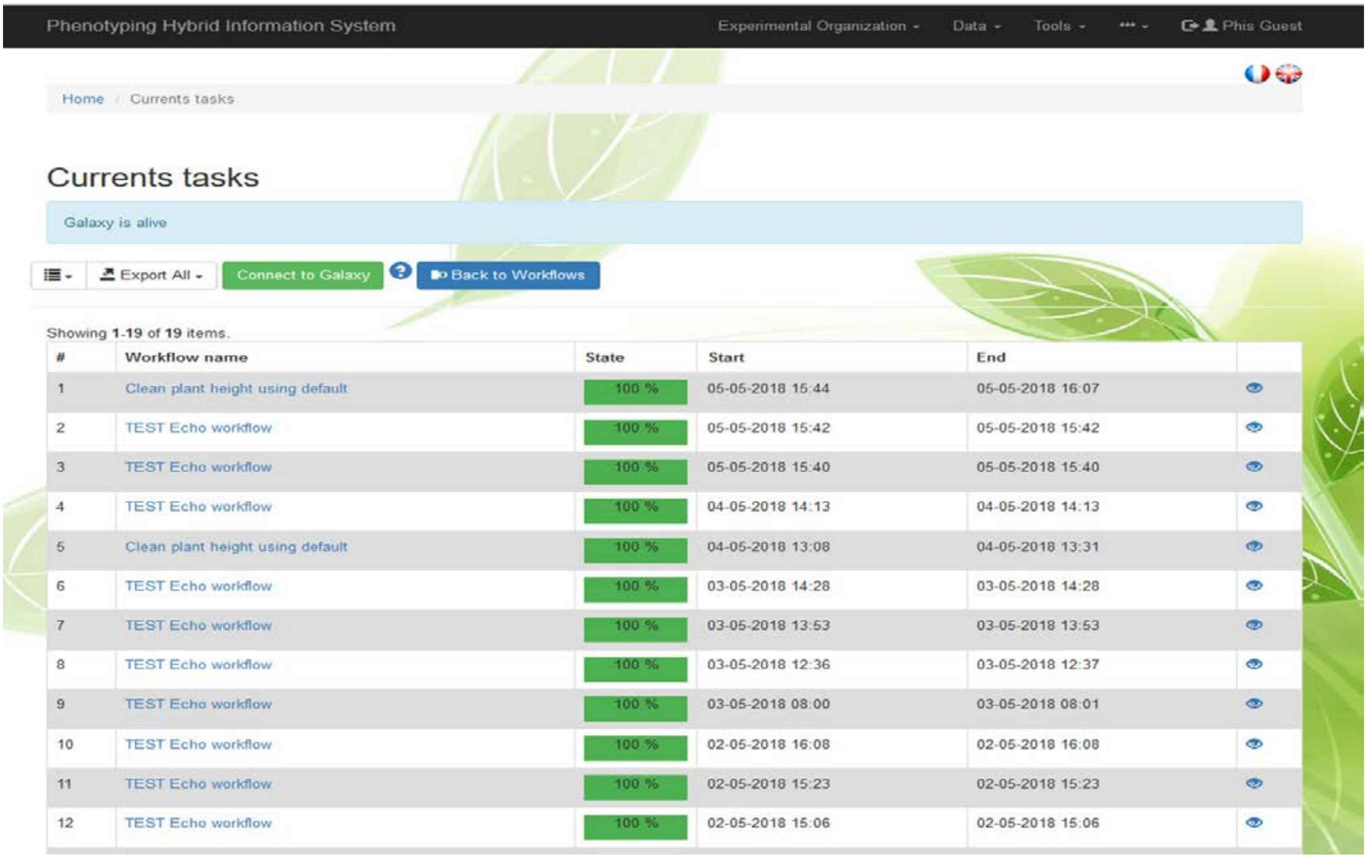

---

[1]Parent *et al*. 2010. Modelling temperature-compensated physiological rates, based on the co-ordination of responses to temperature of developmental processes. *Journal of Experimental Botany* 61: 2057-2069.
