# Supplementary material for: Dealing with multi‐source and multi‐scale information in plant phenomics: the ontology‐driven Phenotyping Hybrid Information System
Source: New Phytol. 2018 Aug 28;221(1):588–601. doi: 10.1111/nph.15385 (PMC6585972; doi:10.1111/nph.15385)
Supplement: Supplementary file 5 — Notes S5 Tools menu of PHIS web user interface. [file NPH-221-588-s005.pdf]

Tools menu

The Tools menu contains installation specific widgets and access to the Web Service API.

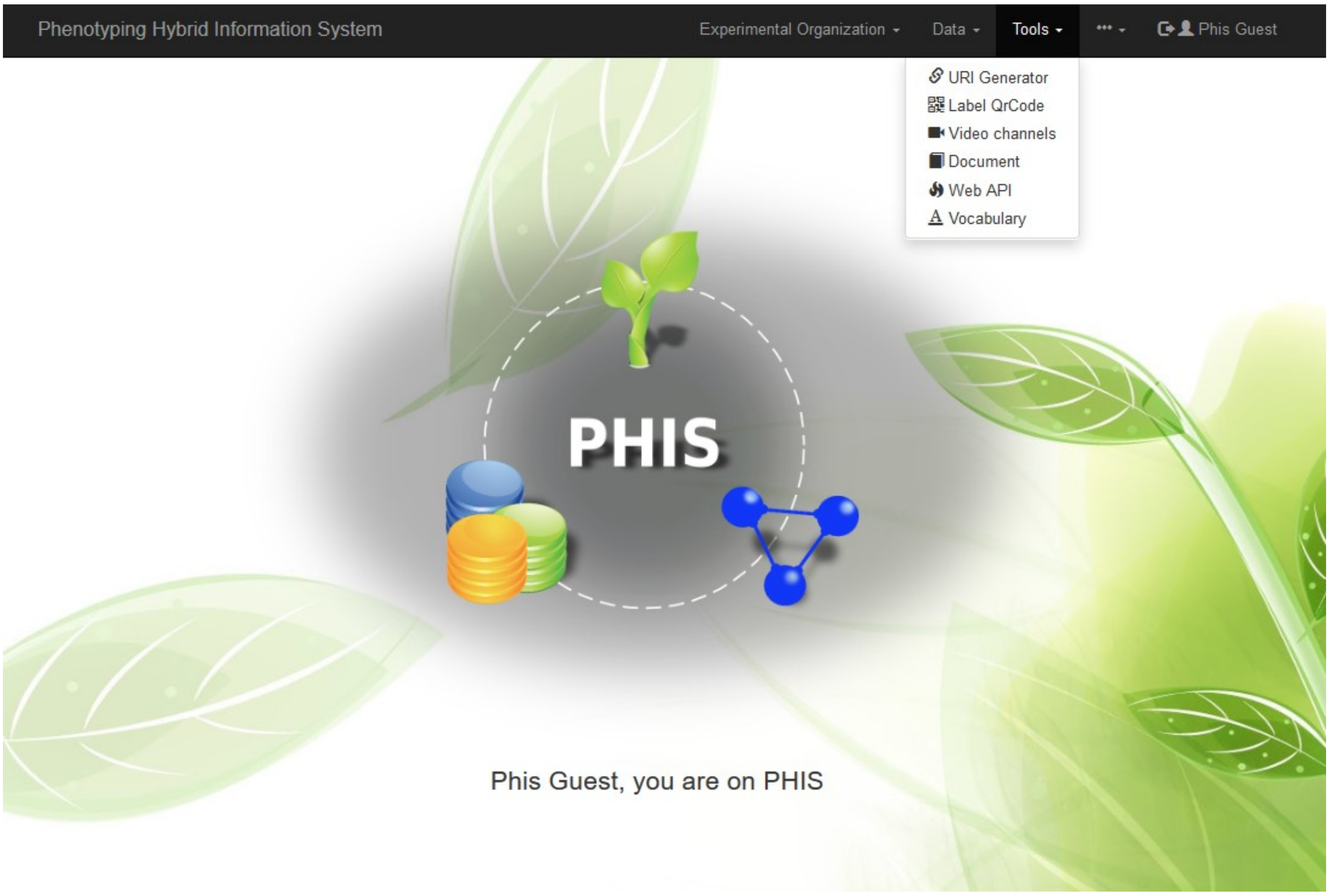

URI Generator

URIs of new objects can be manually created using the URI generator. Upon declaration of a new URI, several fields are required such as `RDF label`, `RDF type`, `Date of creation` and `Comment`.

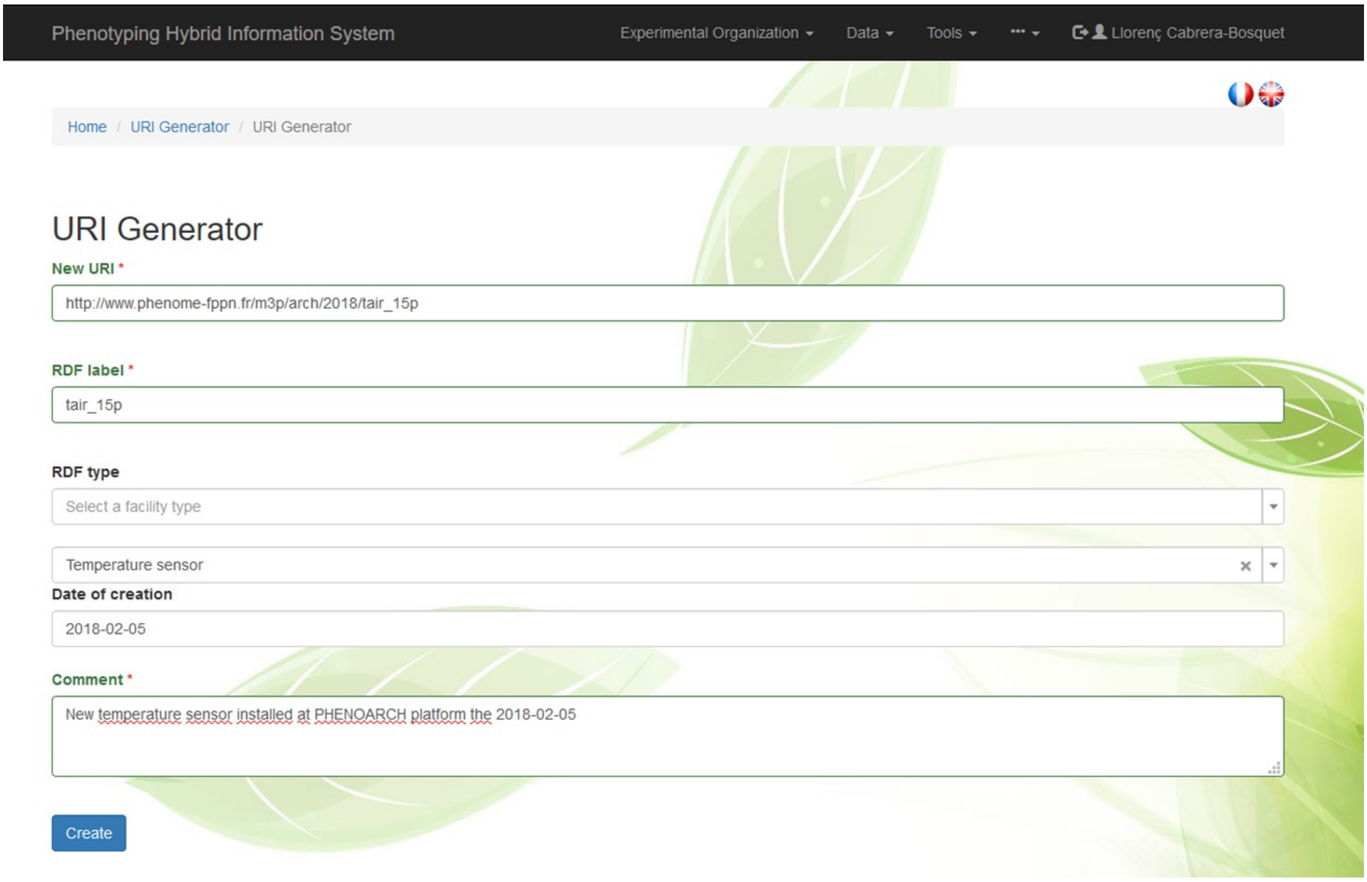

Video Channels

For some installations having CCTVs or webcams, access is provided.

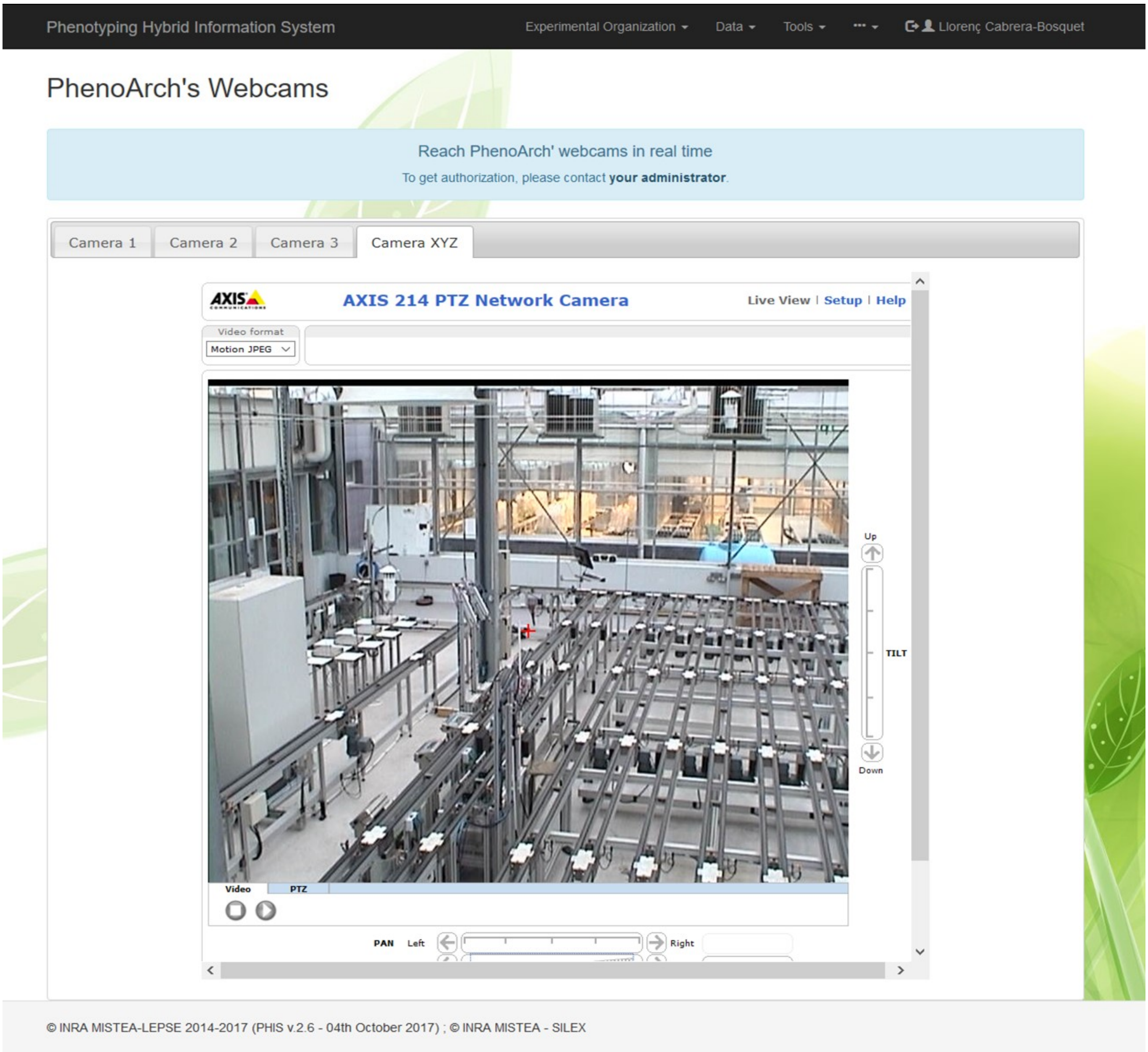

Quick Response Code Generator

The tools menu contains a Quick response (QR) code generator. Qrcode

First, an experiment has to be chosen, and the list of plants or plots can be displayed.

Visualise Plant Tags

Plants or plots of a given experiment have a predefined Tag Pattern (e.g. `CAR_NUMBER/VARIETY/SEEDLOT/SCENARIO/REP/XY/EXPERIMENT`)

Tag patterns can be customised for each experiment. Change tag Pattern

QR codes can be customised using the different items available for each experiment. Configure QrCode Label

For instance, information about the pot/plot number, the experiment, variety, scenario, seed lots, replicates and position of plants or plots can be displayed in QR codes:

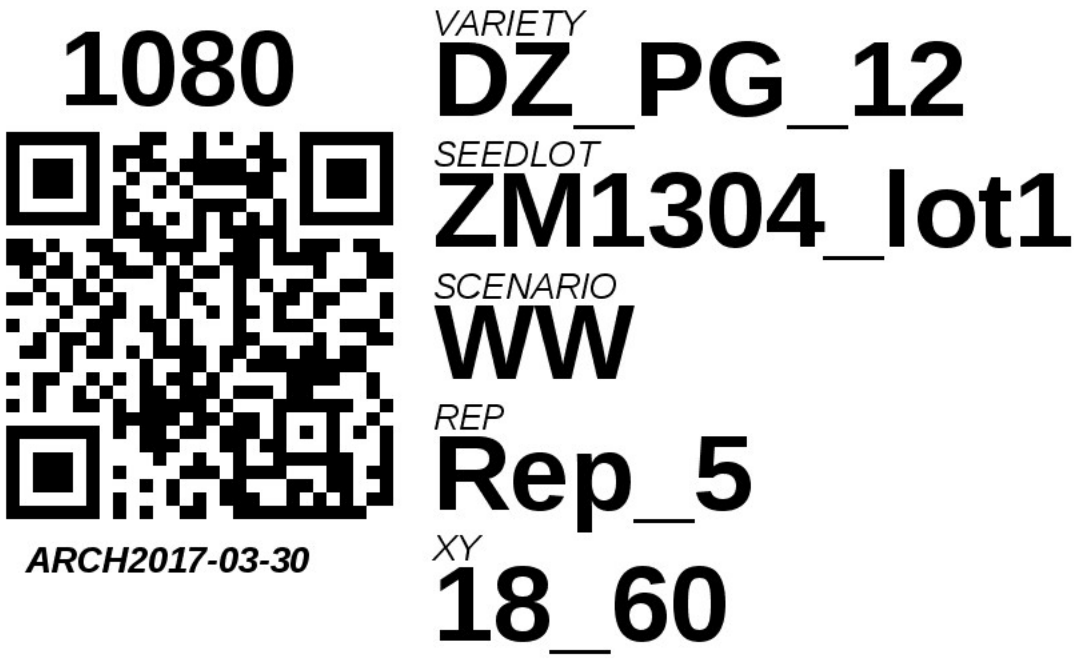

In the example QR code, 1080 and ARCH2017-03-30 stand for the pot number and experimnt name, respectivley

Web Service API

An link to the Web Service API is provided.

Vocabulary

The vocabulary menu contains information about the OEPO and OEEv ontologies as well as all semantic resources used in PHIS.
