# Supplementary material for: Dealing with multi‐source and multi‐scale information in plant phenomics: the ontology‐driven Phenotyping Hybrid Information System
Source: New Phytol. 2018 Aug 28;221(1):588–601. doi: 10.1111/nph.15385 (PMC6585972; doi:10.1111/nph.15385)
Supplement: Supplementary file 6 — Notes S6 Web Service API. [file NPH-221-588-s006.pdf]

# Web Service API

---

The Web Service API enables interoperability and data exchange with external databases and resources, export to computing and modelling platforms and integration of phenomic data into other systems.

The Web Service provides flexible and powerful capabilities for the integration of a diverse and multi-source amount of data including:

- Structured environmental and phenotypic data acquired by the different sensors of the installation (stored in a PostgreSQL database)
- Images stored in the iRODS system
- Non-structured data such as elaborated variables and image analysis stored in the MongoDB
- Rich metadata and knowledge stored in the Triple Store

The versatile use of the Web Service allows one to virtually integrating data from any external client, therefore allowing PHIS to be adapted into other infrastructures. For instance, environmental data from a network of field meteorological stations **(http://w3.avignon.inra.fr/carto/)** and soil sensors **(http://www.agriscope.fr/)** are integrated in PHIS via the Web Service API. Similarly, features extracted from image analyses via a **Python** and **R** pipelines are routinely integrated in PHIS through the Web Service.

PHIS can also export data to external databases, in particular those dedicated to genetic analyses or modelling. For example, export to the **GnpIS** information system[1], member of the **ELIXIR European infrastructure**, allows genome-wide association studies based on the phenotypic datasets organized in PHIS via the collaborative **Breeding API**.

Export to the modelling platform **OpenAlea**[2,3] has allowed calculation of the light interception and radiationuse efficiency of hundreds of maize plants using data obtained in the **M3P installation**[4].

Finally, data search and advanced queries can be performed to remote databases thanks to the Web Service APIs and the inference engines that use the semantics and rules represented in the ontologies.

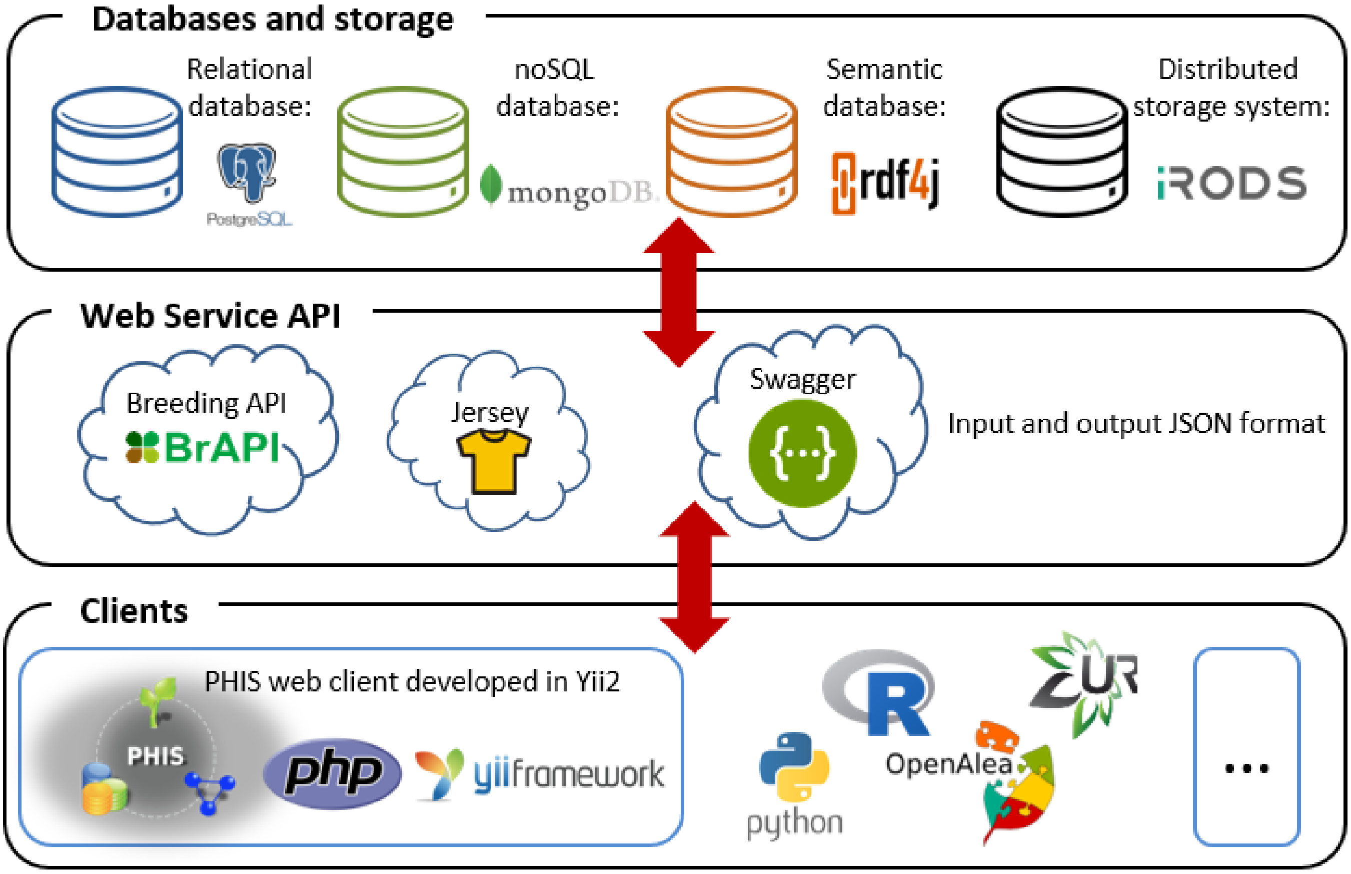


The web service is based on RESTFul (Representational state transfer) developed using **Swagger framework** and all services are available by using URIs. It is developed in Java with Jersey implementation of JAX-RS (Java API for RESTful Web Services) standard. It implements the **Breeding API (BrAPI)**, which specifies a standard interface for plant phenotype databases to serve data to crop breeding applications. Web service outputs use the data-interchange format JSON (JavaScript Object Notation). The Web service is available at **http://www.phis.inra.fr:8080/phenomeapi/api-docs/** to authorized client programs.

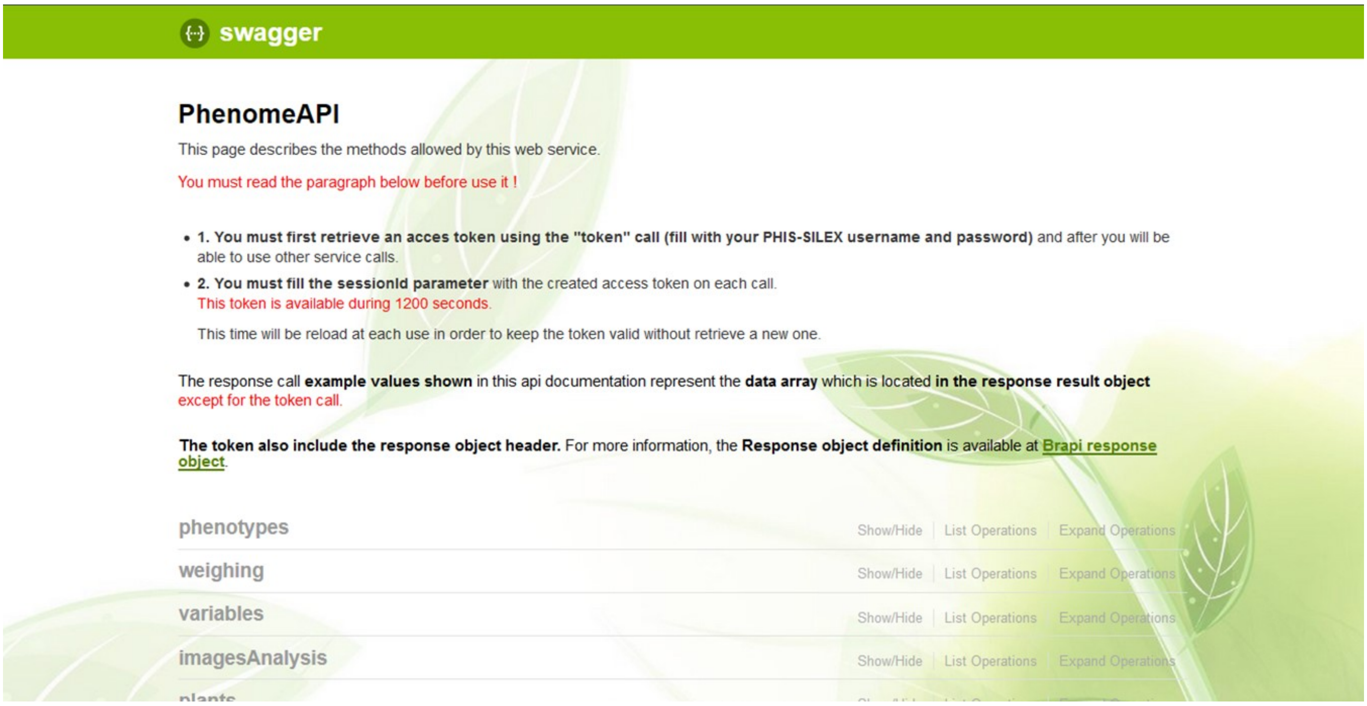

# Getting access rights

The first step to get access to web service consists in retrieving an acces token using the "token" call. A PHIS username and password is necessary to get the token. The guest account "guestphis@supagro.inra.fr" and the password "guestphis" give access to data from public experiments.

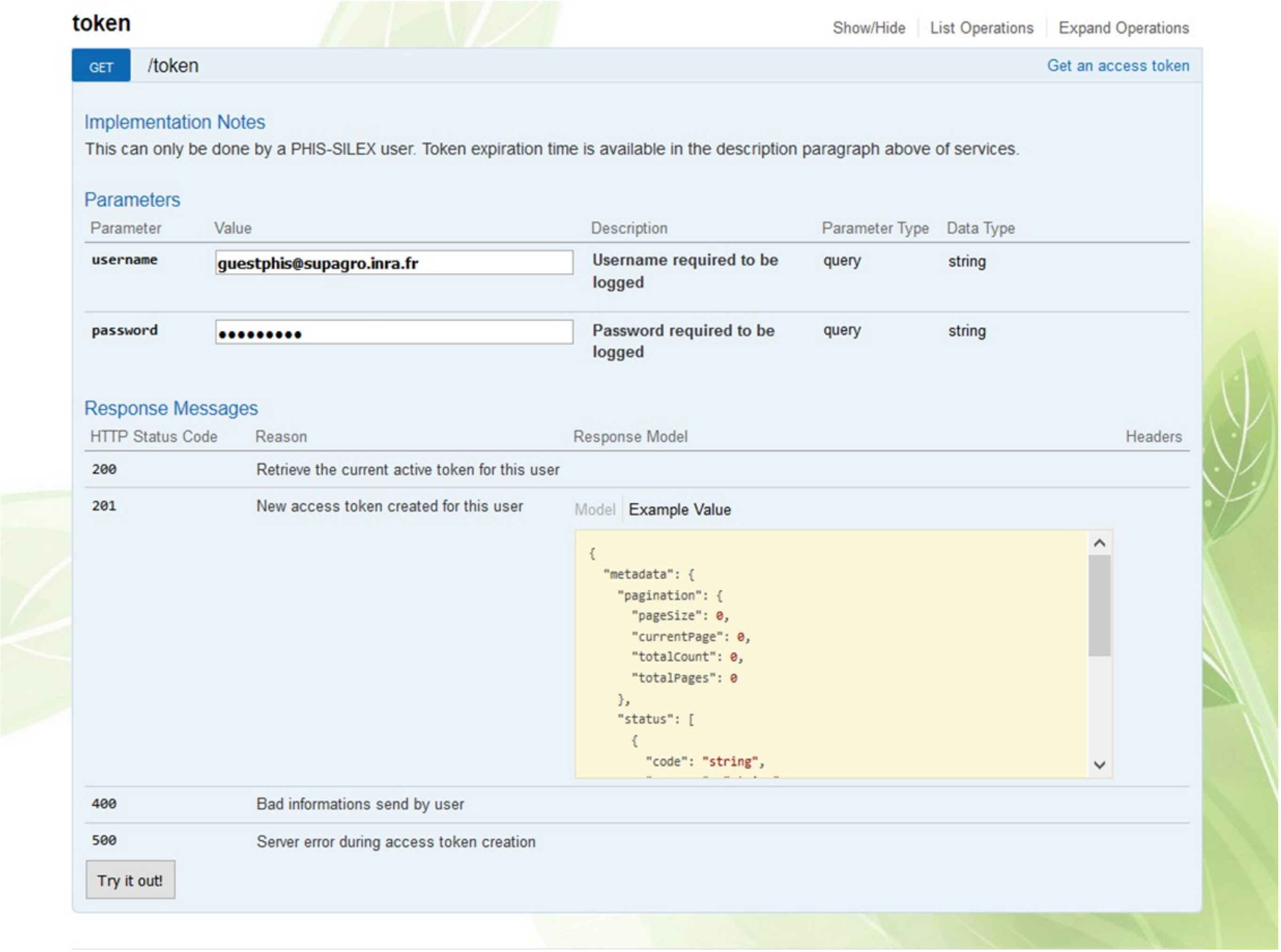


### cURL (client URL request library)

```
curl -X GET --header 'Accept: application/json'
'http://www.phis.inra.fr:8080/phenomeapi/resources/token?
username=guestphis%40supagro.inra.fr&password=guestphis'
```

### Request URL

```
http://www.phis.inra.fr:8080/phenomeapi/resources/token?
username=guestphis%40supagro.inra.fr&password=guestphis
```

### Response body

```
```

```
{
  "metadata": {
    "pagination": null,
    "status": null,
    "datafiles": []
  },
  "session_token": "b3c857e499fd52c51ad70fc64ffafde9"
}
```


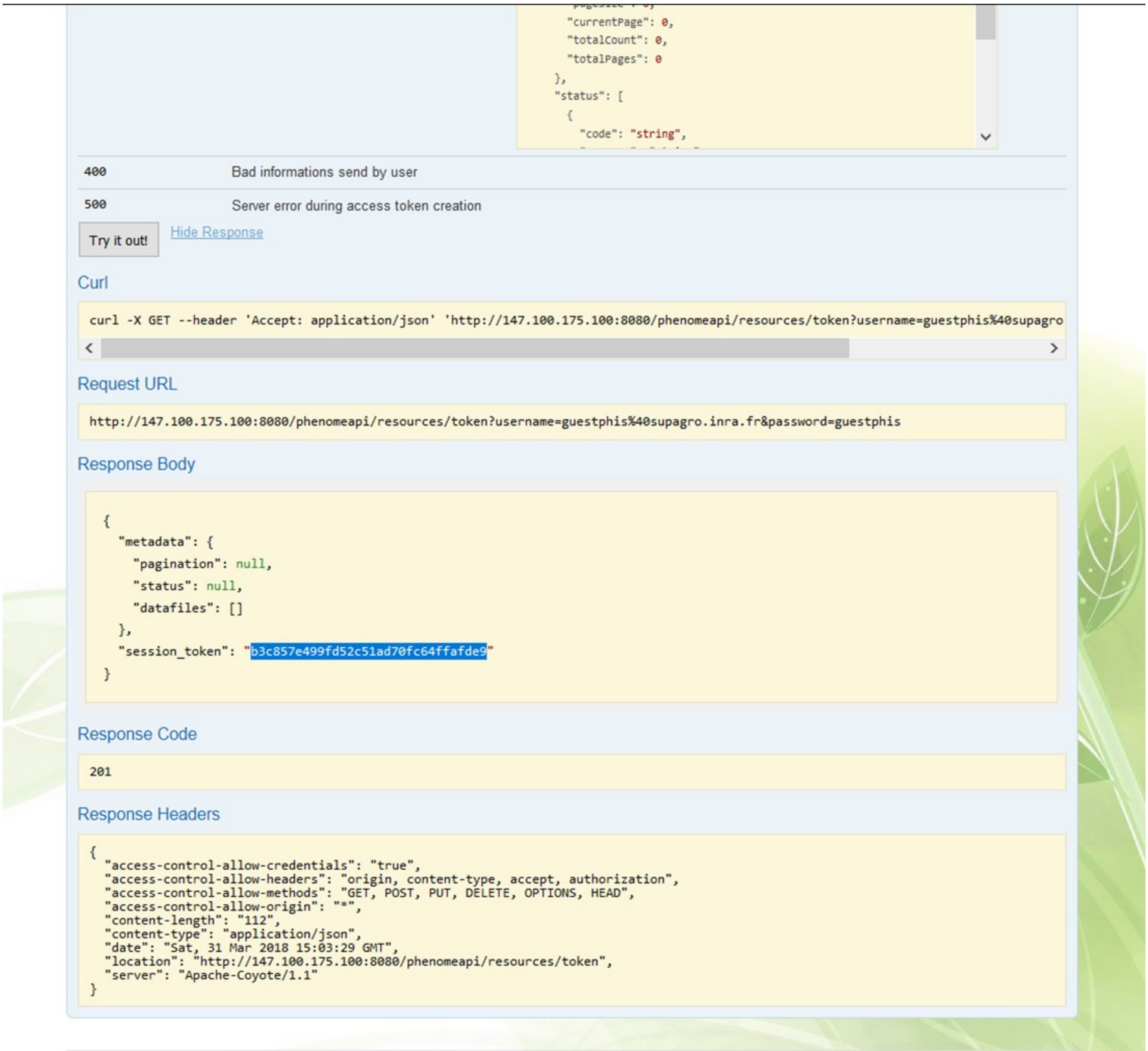


In the example shown here the "session_token": "b3c857e499fd52c51ad70fc64ffafde9" is obtained. This token is available during 1200 seconds. This time is reloaded at each use in order to keep the token valid without necessity to retrieve a new one.

## GET, POST and PUT data

The Web Service allows to **GET**, **POST** and **PUT** different available data including:

- Projects
- Environment
- Plants
- Experiments
- Weighing and Watering data
- Phenotypes
- Image analysis
- Phenotypic observations
- Variables
- Environmental data
- Documents
- Events and annotations

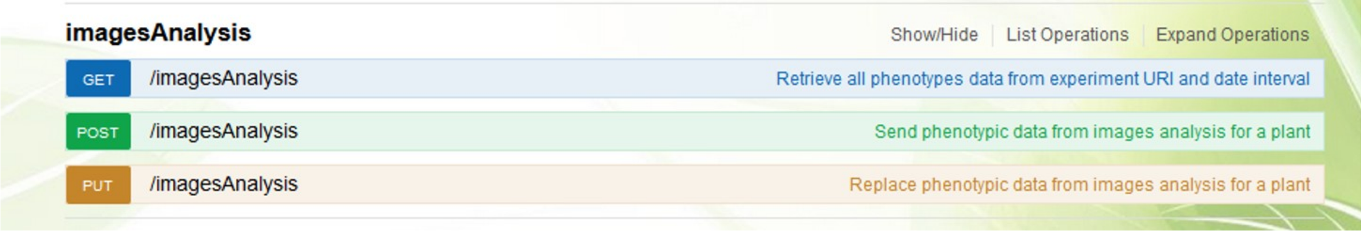


Tu **GET**, **POST** or **PUT** data, the sessionid parameter needs to be filled with the *session_token*.

# Use case

In this example the Web Service is used to **GET** *convexHullArea* data from images captured in the *http://www.phenome-fppn.fr/m3p/ARCH2017-03-30* experiment corresponding to *labelView* "side90".

**cURL (client URL request library)**

```
curl -X GET --header 'Accept: application/json'
'http://www.phis.inra.fr:8080/phenomeapi/resources/imagesAnalysis?
experimentURI=http%3A%2F%2Fwww.phenome-fppn.fr%2Fm3p%2FARCH2017-
0330&labelView=side90&variablesName=convexHullArea&pageSize=5&page=0&sessionId=7d4df
8b9e0892977f99046fe2e434c87'
```

**Request URL**

```
http://www.phis.inra.fr:8080/phenomeapi/resources/imagesAnalysis?
experimentURI=http%3A%2F%2Fwww.phenome-fppn.fr%2Fm3p%2FARCH2017-
0330&labelView=side90&variablesName=convexHullArea&pageSize=5&page=0&sessionId=7d4df
8b9e0892977f99046fe2e434c87
```

**Response body**

As a response, a JSON is obtained. A total count of 49643 results is obtained.

```
{
  "metadata": {
    "pagination": {
      "currentPage": 0,
      "pageSize": 5,
      "totalCount": 49653,
      "totalPages": 9931
    },
    "status": null,
    "datafiles": []
  },
  "result": {
"data": [
      {
        "plantURI": "http://www.phenome-fppn.fr/m3p/arch/2017/c17000100",
        "experimentURI": "http://www.phenome-fppn.fr/m3p/ARCH2017-03-30",
        "imageUri": "http://www.phenome-fppn.fr/m3p/arch/2017/ic17002281946",
"labelView": "side90",
        "date": "2017-05-18 05:55:05.568+02:00",
        "variableCodeId": "convexHullArea_computed_square pixels",
"value": 1412764,
        "confidence": "unspecified",
        "stationId": 6
      },
      {
        "plantURI": "http://www.phenome-fppn.fr/m3p/arch/2017/c17000120",
        "experimentURI": "http://www.phenome-fppn.fr/m3p/ARCH2017-03-30",
        "imageUri": "http://www.phenome-fppn.fr/m3p/arch/2017/ic17002250263",
"labelView": "side90",
        "date": "2017-05-16 07:57:15.540+02:00",
        "variableCodeId": "convexHullArea_computed_square pixels",
"value": 145187,
        "confidence": "unspecified",
        "stationId": 6
      },
      {
        "plantURI": "http://www.phenome-fppn.fr/m3p/arch/2017/c17000119",
        "experimentURI": "http://www.phenome-fppn.fr/m3p/ARCH2017-03-30",
        "imageUri": "http://www.phenome-fppn.fr/m3p/arch/2017/ic17002250250",
"labelView": "side90",
        "date": "2017-05-16 07:56:40.498+02:00",
        "variableCodeId": "convexHullArea_computed_square pixels",
"value": 680346.5,
        "confidence": "unspecified",
        "stationId": 6
      },
      {
        "plantURI": "http://www.phenome-fppn.fr/m3p/arch/2017/c17000118",
        "experimentURI": "http://www.phenome-fppn.fr/m3p/ARCH2017-03-30",
        "imageUri": "http://www.phenome-fppn.fr/m3p/arch/2017/ic17002250237",
"labelView": "side90",
        "date": "2017-05-16 07:56:05.074+02:00",
        "variableCodeId": "convexHullArea_computed_square pixels",
```

```
          "value": 1436071,
          "confidence": "unspecified",
          "stationId": 6
        },
        {
          "plantURI": "http://www.phenome-fppn.fr/m3p/arch/2017/c17000117",
          "experimentURI": "http://www.phenome-fppn.fr/m3p/ARCH2017-03-30",
          "imageUri": "http://www.phenome-fppn.fr/m3p/arch/2017/ic17002250224",
          "labelView": "side90",
          "date": "2017-05-16 07:55:29.719+02:00",
          "variableCodeId": "convexHullArea_computed_square pixels",
          "value": 1012235,
          "confidence": "unspecified",
          "stationId": 6
        }
      ]
    }
  }
```


# R Client

The Web Service is accessible to R clients through a R Package "phisWSClientR". The package and user and developper documentation are available at https://github.com/OpenSILEX/phis-ws-client-R under a GNU General Public License.

# References


- [1]Steinbach D, Alaux M, Amselem J, Choisne N, Durand S, Flores R, Keliet AO, Kimmel E, Lapalu N, Luyten I, et al. 2013. GnpIS: an information system to integrate genetic and genomic data from plants and fungi. Database: The Journal of Biological Databases and Curation 2013: bat058.

- [2]Pradal C, Dufour-Kowalski S, Boudon F, Fournier C, Godin C. 2008. OpenAlea: a visual programming and component-based software platform for plant modelling. *Functional Plant Biology* 35: 751-760.

- [3]Pradal C, Fournier C, Valduriez P, Cohen-Boulakia S 2015. OpenAlea: Scientific Workflows Combining Data Analysis and Simulation.In Gupta A, Rathbun S. 27th International Conference on Scientific and Statistical Database Management (SSDBM 2015). San Diego, CA, USA: ACM - Association for Computing Machinery, New York 978-1-4503-3709-0.

- [4]Cabrera-Bosquet L, Fournier C, Brichet N, Welcker C, Suard B, Tardieu F. 2016. High-throughput estimation of incident light, light interception and radiation-use efficiency of thousands of plants in a phenotyping platform. *New Phytologist* 212: 269-281.
