# Supplementary material for: Dealing with multi‐source and multi‐scale information in plant phenomics: the ontology‐driven Phenotyping Hybrid Information System
Source: New Phytol. 2018 Aug 28;221(1):588–601. doi: 10.1111/nph.15385 (PMC6585972; doi:10.1111/nph.15385)
Supplement: Supplementary file 7 — Notes S7 OEPO and OEEv ontologies and annotations. [file NPH-221-588-s007.pdf]

Ontologies

PHIS relies on two application ontologies compliant with the standards of the **Ontology Web Language**. The ontologies are available at the **Agroportal**.

OEPO

The **Ontology for Experimental Phenotypic Objects (OEPO)**, allows assigning types (e.g. Experimental organization and context, Environment type, Germplasm, Physical Object) to objects involved in phenotyping experiments and defining specialization hierarchy between them according to the specificities of the installations and experiments.

The OEPO ontology contains three main groups of objects, namely *Experimental Condition*, *Scientific Object* and *Germplasm Descriptor*.

Experimental condition

The *Experimental Condition* describes the set of environmental conditions, infrastructure and supporting equipment or resources necessary to conduct a given experiment in which plants, plots or group of plants are raised.

A number of the terms and definitions follow recommendations adopted by the ESFRI listed project **EMPHASIS** and other resources such as the **Plant Experimental Conditions Ontology** and the **Plant Phenotype Experiment Ontology**, are used for establishing relations and describing experiments.

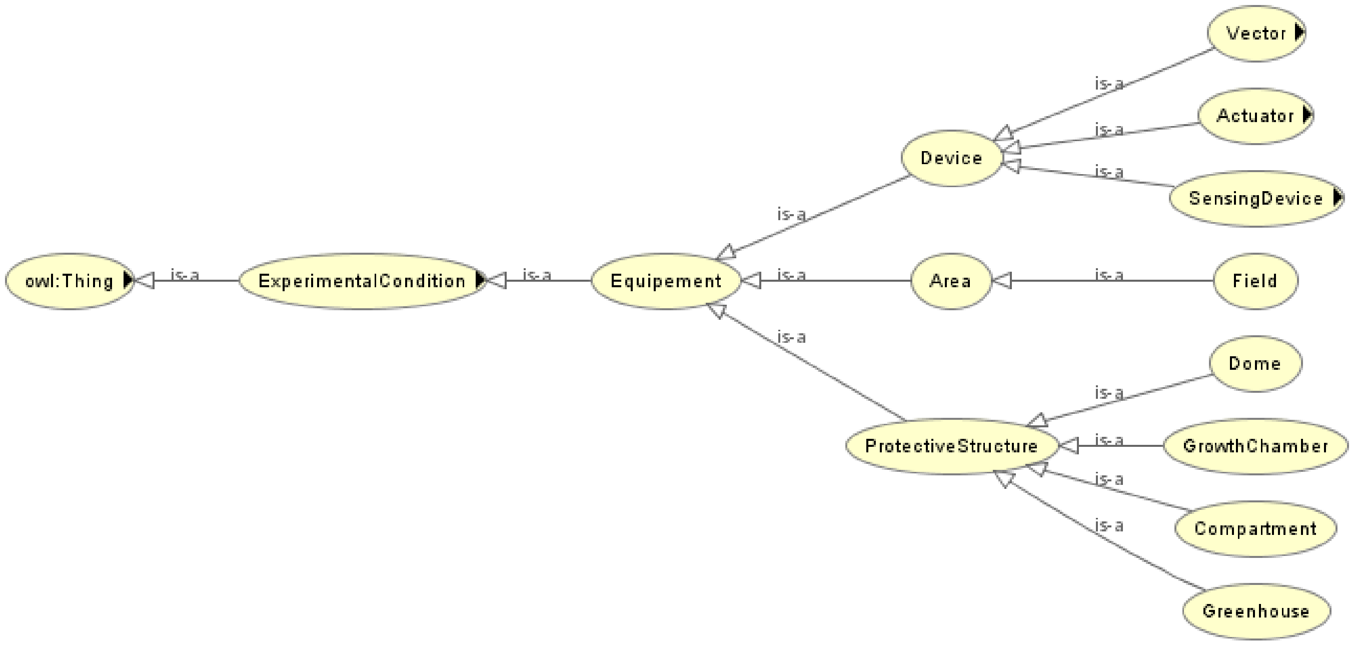

For instance, a *device* is a sub-category of *equiment* containing *vectors*, *actuators* and *sensing devices*

- *Vectors* are *devices* that either transport plants to a given site for phenotyping, or carry sensors in a greenhouse (e.g. conveyor belt, gripping arm, robot) or in the field (e.g. UAV, gantry, field robot). By extension, a vector can also be a group of persons producing hand observations.

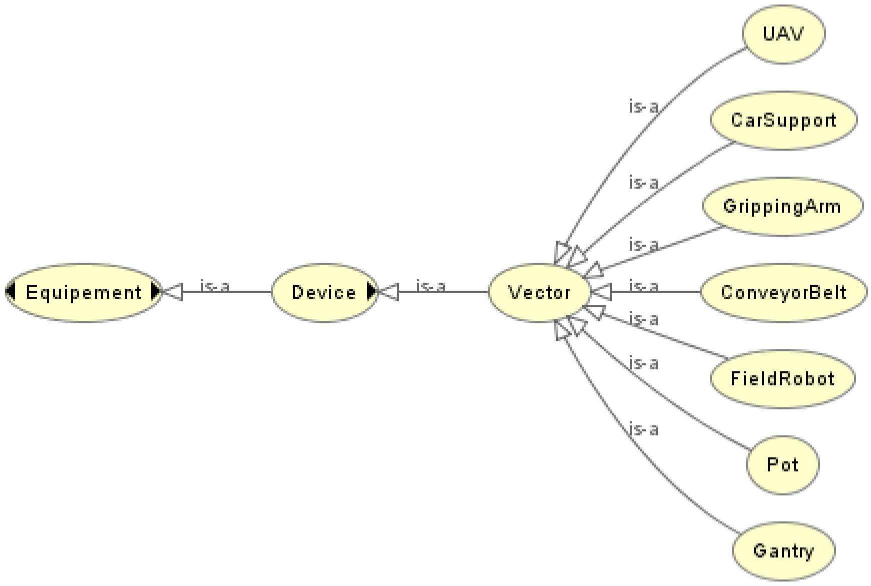

- *Actuators* are *devices* that usually are responsible for moving and controlling a mechanism or system, for example by opening a valve or opening shadows in a greenhouse.

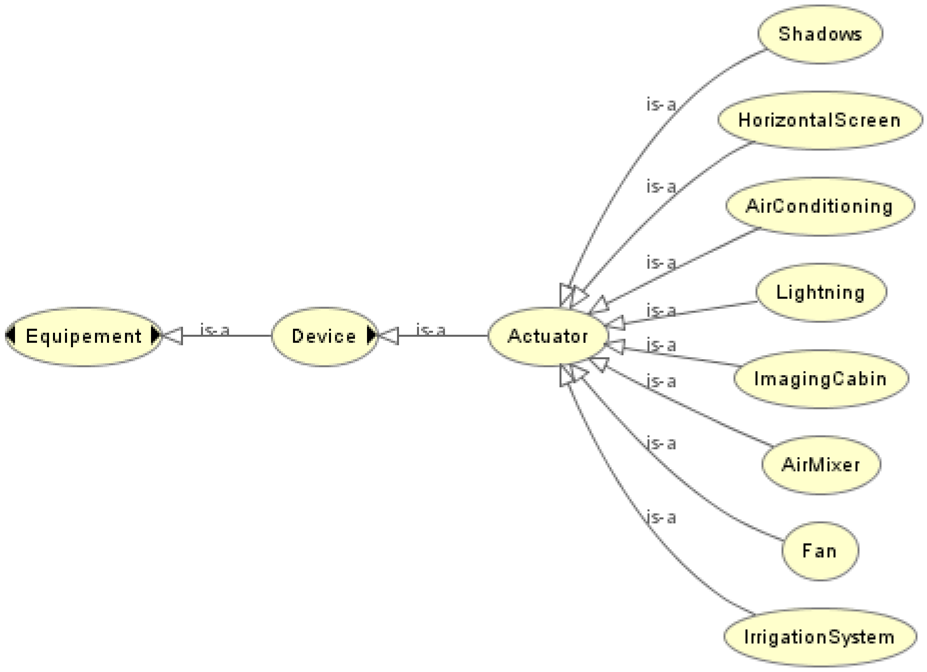

- *Sensing devices* are *devices* providing numerical data, e.g. environment sensors or cameras. Environmental sensors follow the **Semantic Sensor Network Ontology**. For instance, a *Quantum sensor* is a *sensing device* and a sub-type of *Radiation sensor*.

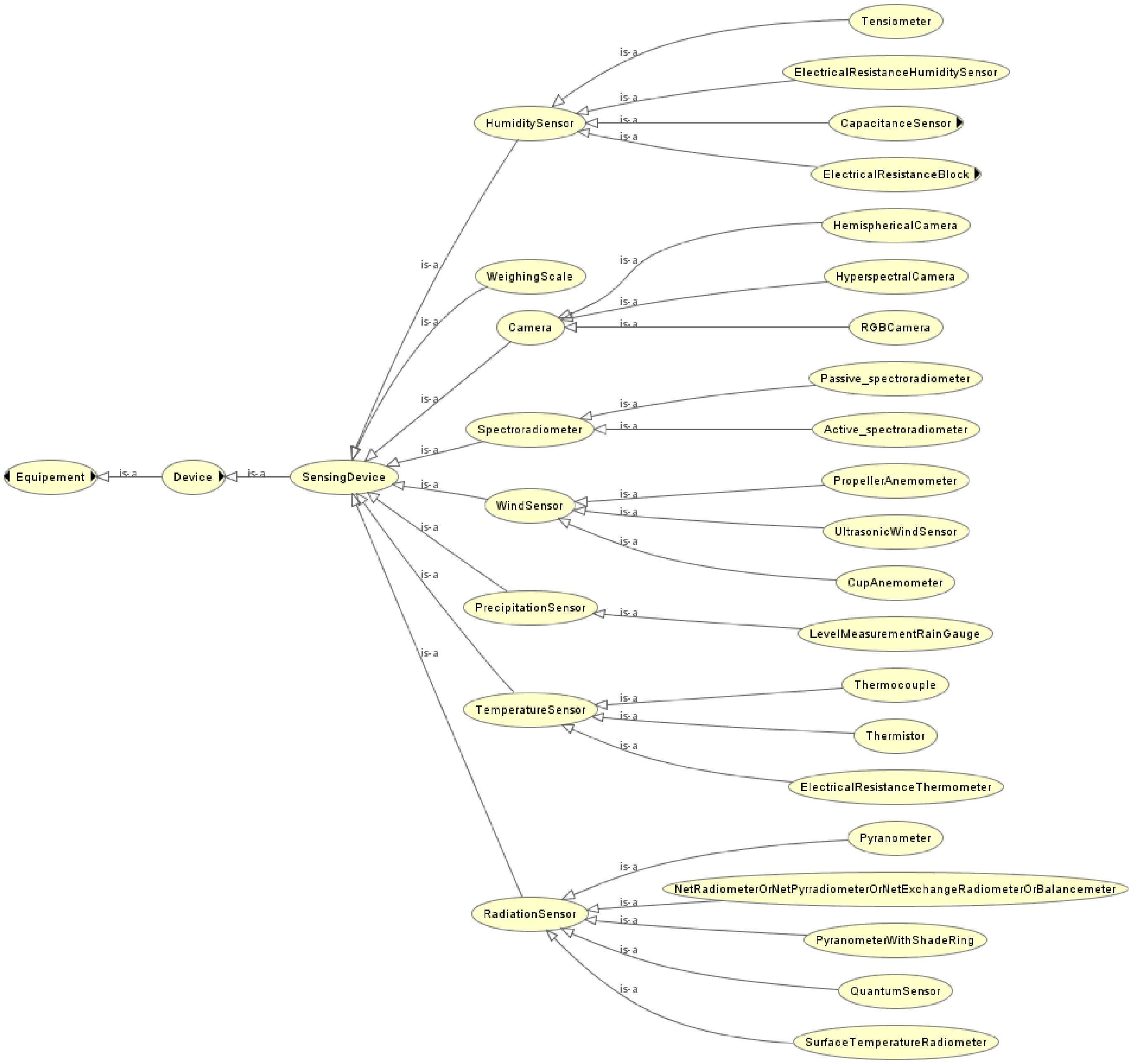

Scientific objects

Scientific objects correspond to the different plant organization levels studied such as *whole plants* (the organism originating from a single seed, callus, rhizome or any other propagation mean), or *plots* (a community of plants located on a defined area of a field), but also other concepts defined at lower spatial scale (e.g. *leaf*, *silk*, *ear Inflorescence*). Terms present in this ontology map onto existing ontologies providing relevant concept hierarchies related to the anatomy, structure and phenotype of plants described in the **Planteome project**[1] such as the **Crop Ontology**[2], the **Plant Ontology**[3,4,5], the **PATO**, and other ontological resources such as the **AGROVOC**[6].

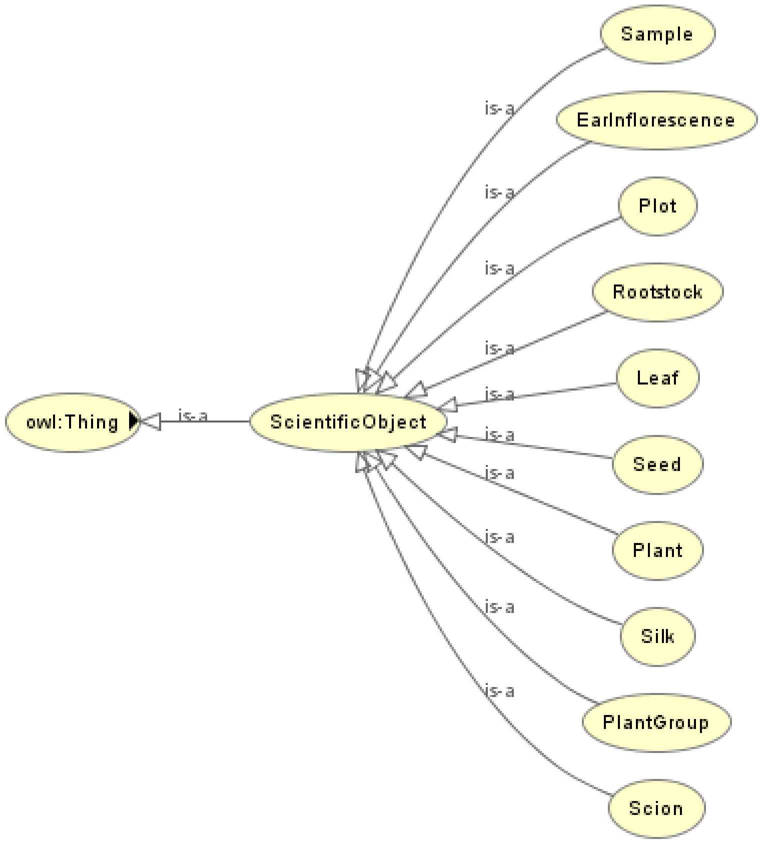

Different semantic ressources such as the **Relations Ontology**[7] and the **OGC GeoSPARQL** are also used to describe object properties.

For instance, the **RO:0000056** term is used do describe => *<Plant>* *<partcipatesIn>* *<Experiment>*, or the **http://www.opengis.net/ont/geosparql#hasGeometry** is used to describe => *<Plot>* *<hasGeometry>* *<"GPS coordinates">*

Germplasm Descriptor

GermplasmDescriptor refer to the different attributes related to a germplasm entity incuding the *Genus*, *Species*, *AccessionName*, *AccessionNumber*, *Variety*, *Seedlot* and *SeedLotSample*. These attributes follow the **FAO/Bioversity Multi Crop Passport Descriptors**[8,9].

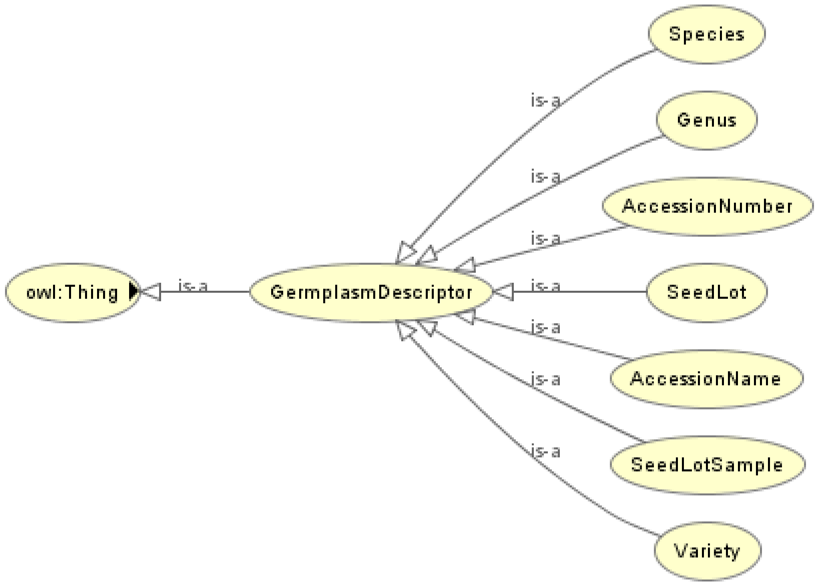

OEEv

The **Ontology of Experimental Events (OEEv)**, characterises events that occur during an experiment, e.g. dates of sowing, application of a given treatment, harvesting, measurements or sampling for -omic measurements, or any category of technical problem. The OEEv ontology contains **four** groups of events, namely *Facicility Management*, *Scientific Object Management*, *Trouble* and *Displacement*.

Facility Management

The *Facility Management* refers to the set of actions related to the management and maintenance infrastructure and supporting equipment. It describes events related to *servicing*, *maintenance* and *calibration* of *equipment* or *installations*.

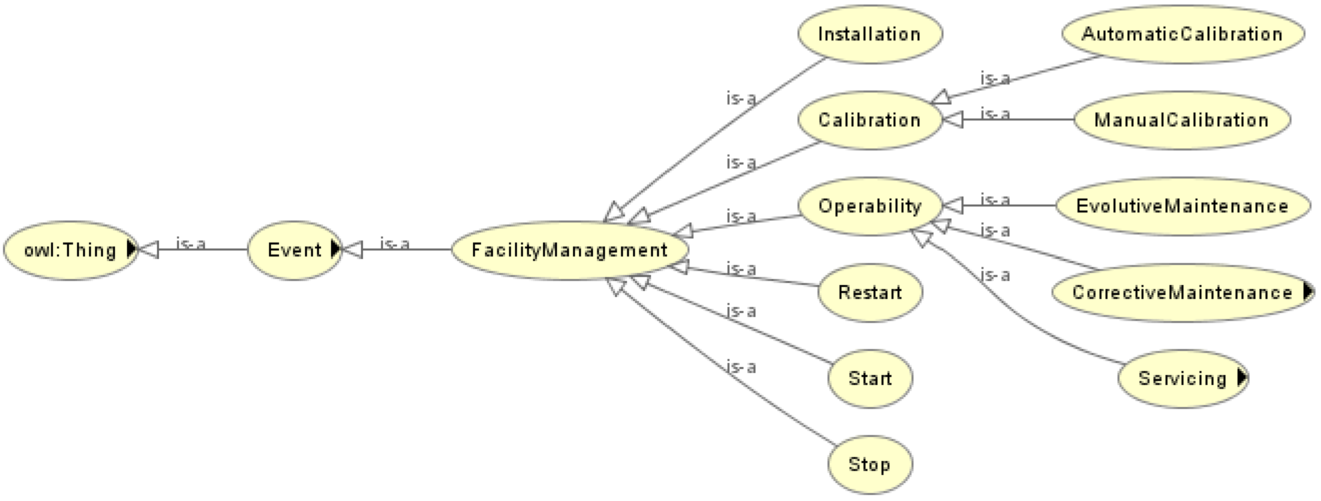

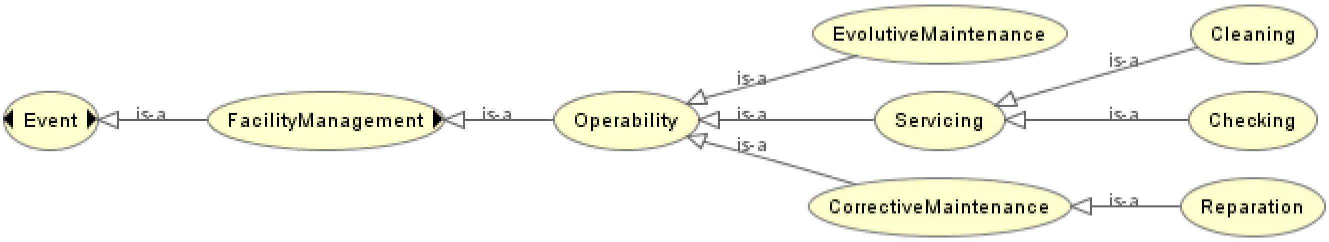

Scientific Object Management

The *Scientific Object Mangament* contains different sub-categories of events related to the typical operations conducted on plants or plots in experiments:

- *AddingProduct* refers to the application of products such as fertilizers
- *Clipping* is related to the installation of accessories surch as embeded sensors
- *Detasseling* refres to removal of the tassel of maize
- *Harvesting* refers to the harvest of the plant
- *Irrigation* describes irrgation events
- *Thinning* refers to the removal of some plants, or parts of plants, to make room for the growth of others
- *Treatment* related to either *curative* or *preventive* treatments applied to plants
- *PhenolgyScoring* refers to the set of events related to the different phenological stages of plants.
- *Potting* aplies to pot experiments
- *Sowing* applies for both field and pot experiments

- *Stacking* refers to the action of putting plant tutors

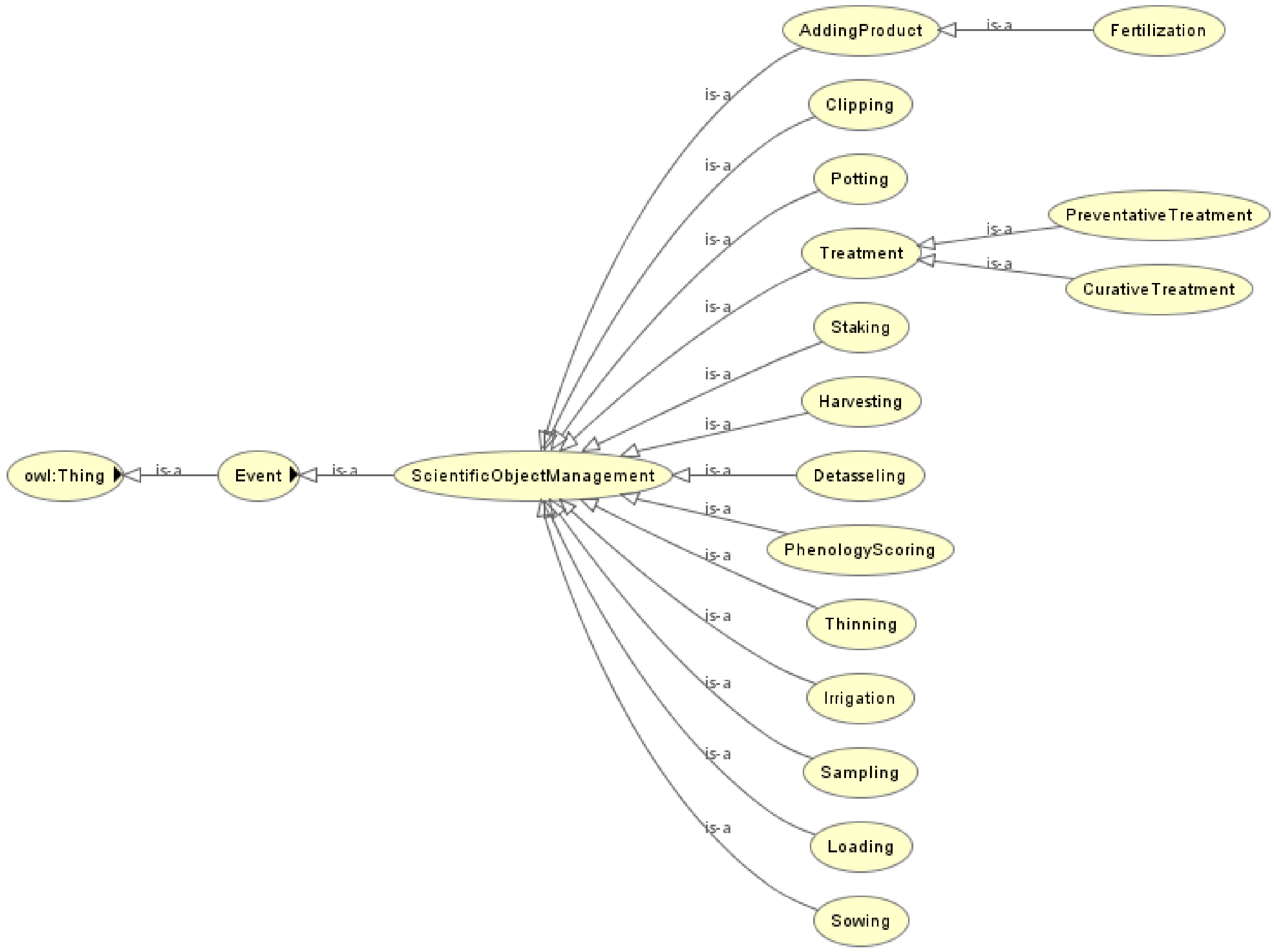

Trouble

The event *Trouble* is defined by three different sub-categories:

- *Breakdown* (sensor or conveyor)
- *Dysfunction* (sensor fault, irrigation trouble)
- *Incident* (a pot falls down, a leaf is blocked in an imaging cabin, lodging of a plot, human error, etc.)

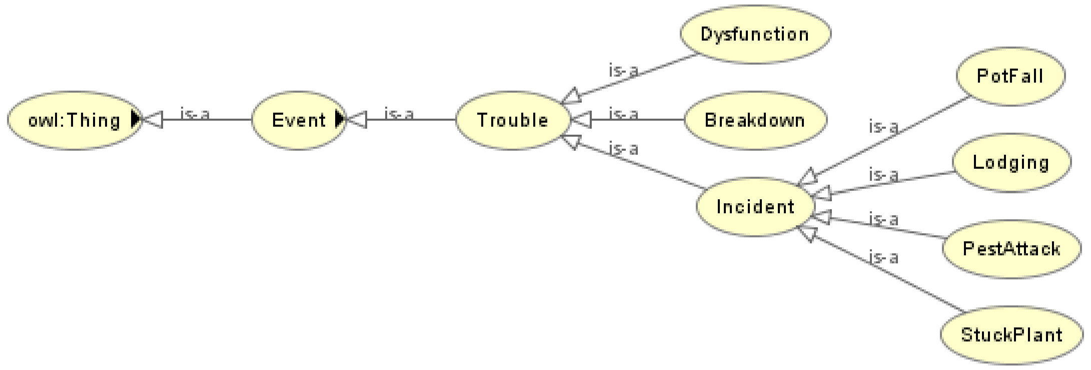

Several subtypes of incidents that ofter occur in platfortm or field experiments have been declared including:

- *PotFall*
- *Lodging*

- *StuckPlant*
- *PestAttack*

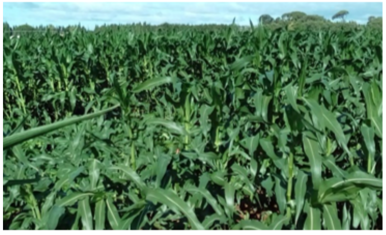

Lodging

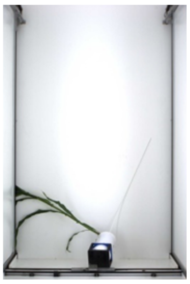

Stuck plant

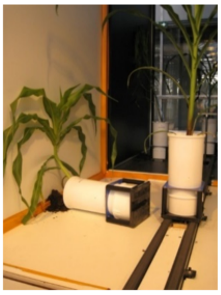

Pot fall

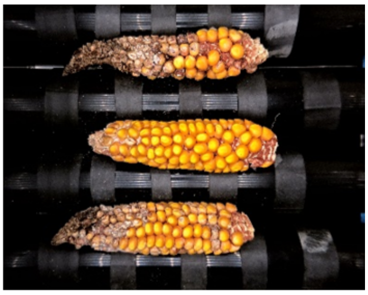

Pest attack

Displacement (object tracking)

The *Displacement* event has been created to follow the position in time and space of the different objects participating in experiments. For instance, plants are often transferred, during a single experiment, between installations or compartments with different environmental conditions. Similarly, sensors can be replaced or moved to different positions in the field or greenhouse. The *MoveTo* and *MoveFrom* concepts allow tracking objects:

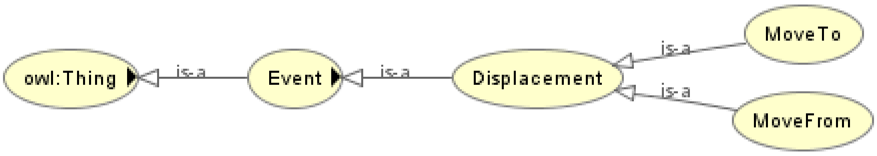

Events or simple annotations are annotated as shown in the following diagram following the **Open Annotation Data Model** that allows assigning motivation and purpose attributes to annotations (e.g. oa:describing, oa:identifying, oa:linking, oa: replying, etc.). **Dublin Core** and **foaf** specifications such as *dcterms:created*, *dcterms:creator*, *foaf:Agent* are also used.

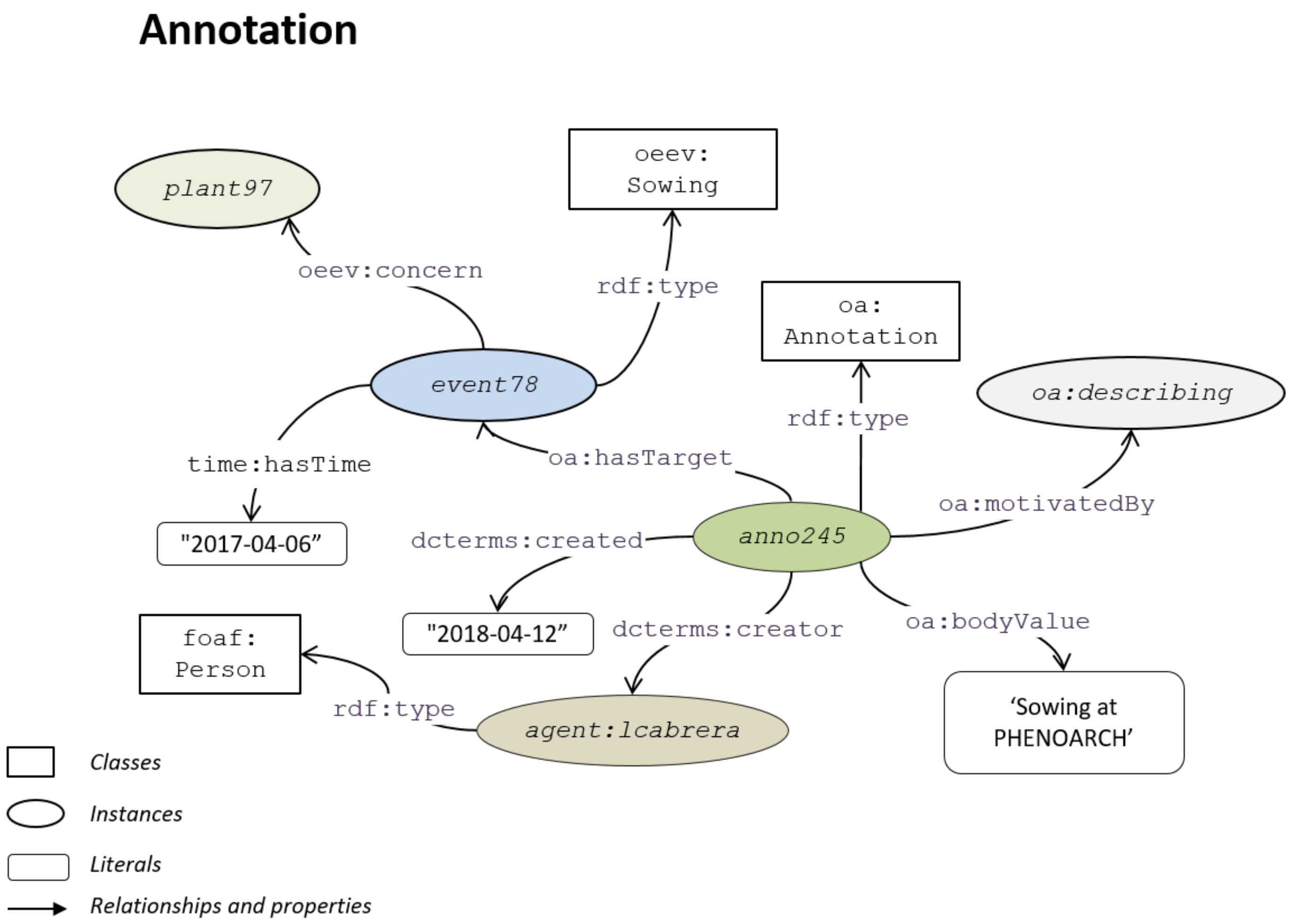

- Instances are depicted as coloured ellipses.
- Classes are depicted as white rectangles.
- Literals are depicted as white lozenges.
- Relationships and properties are depicted as black lines.
- The different prefixes stand for:
    - oeev: http://www.phenome-fppn.fr/vocabulary/2018/oeev#
    - oa: http://www.w3.org/ns/oa#
    - foaf: http://xmlns.com/foaf/spec/
    - rdf: http://www.w3.org/1999/02/22-rdf-syntax-ns#
    - time: http://www.w3.org/2006/time#
    - dcterms: http://purl.org/dc/terms/
    - agent: http://www.phenome-fppn.fr/id/agent/

Use case

The following semantic graph represents how **OEPO** and **OEEv** ontologies interact for representing some of the characteristics and events of plot 97 in the field experiment DIA2017-05-19.

Thus, the ***plot97*** is of type ***plot***, participates in the experiment ***DIA2017-05-19***, and has a spatial geometry (***GPScoord***) defined following the **Well-known text standard**. ***plot97*** contains the ***plant144*** (which is of type ***plant***) which in turn contains ***leaf461*** (which is of type ***leaf***). All these temrms and semantic relationships between objects are defined following the **OEPO** ontology. On the other hand, **OEEv** ontology allows describing events cocerning the different objects involved in an experiment. In the example presented here,

the ***event54*** that concerns ***plot97*** is of type ***Incident*** and occurred tfssn he **"2017-06-29"**. ***event54*** is associated to the ***anno65*** which is of type ***Annotation***. This annotation, has a creator (***Romain***), a date of annotation (**"2017-08-10"**) and a body value describing the event (**"Plot lodged after the storm"**).

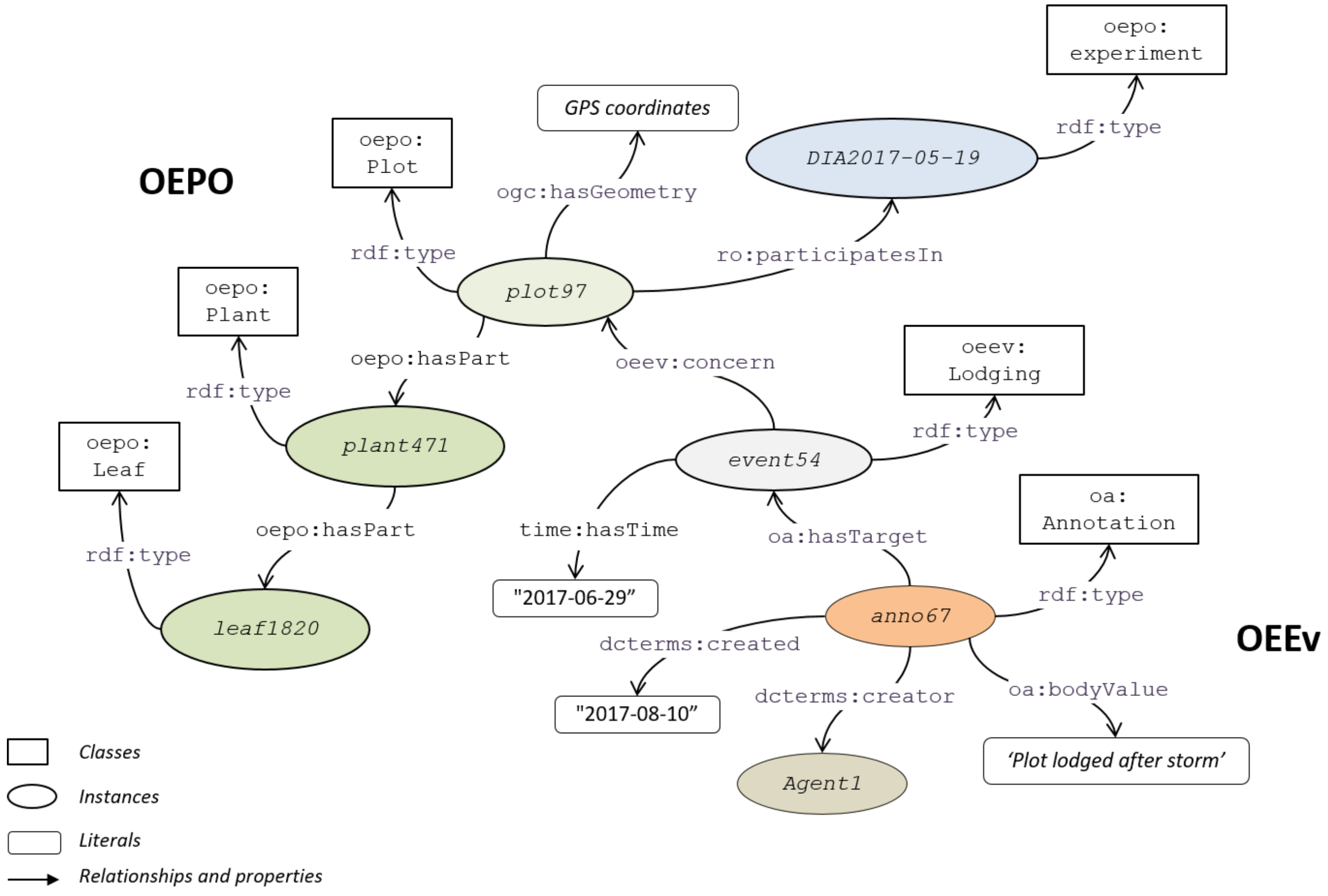

- Instances are depicted as coloured ellipses.
- Classes are depicted as white rectangles.
- Literals are depicted as white lozenges.
- Relationships and properties are depicted as black lines.
- The different prefixes stand for:
    - oeev: http://www.phenome-fppn.fr/vocabulary/2018/oeev#
    - oepo: http://www.phenome-fppn.fr/vocabulary/2018/oepo#
    - oa: http://www.w3.org/ns/oa#
    - foaf: http://xmlns.com/foaf/spec/
    - rdf: http://www.w3.org/1999/02/22-rdf-syntax-ns#
    - time: http://www.w3.org/2006/time#
    - dcterms: http://purl.org/dc/terms/
    - agent: http://www.phenome-fppn.fr/id/agent/

For better legibility objects are labelled with shortened alias names rather than using full URIs:

- ***plot97***: http://www.phenome-fppn.fr/diaphen/2017/o17000097
- ***plant471***: http://www.phenome-fppn.fr/diaphen/2017/17000471
- ***leaf1820***: http://www.phenome-fppn.fr/diaphen/2017/l17001820
- ***DIA2017-05-19***: http://www.phenome-fppn.fr/diaphen/DIA2017-05-19
- ***GPScoord***: Polygon ((3.973276 43.61203, 3.973306 43.61201, 3.973354 43.61205, 3.973324 43.61207, 3.973276 43.61203))

- ***Agent1***: http://www.phenome-fppn.fr/id/agent/rchapuis
- ***event54***: http://www.phenome-fppn.fr/id/event/571e22a0-7cd7-42aa-92be-dc68d1f62faa
- ***anno65***: http://www.phenome-fppn.fr/id/annotation/3fdcada7-a532-4914-8e9e-889aa1c53459

Note that not all objects and events associated to ***plot97*** are represented in this graph. Fulls details can be found online in the PHIS user interface by searching for plot 97 in the different menus (e.g. **Events**, **Scientific objects**).

References

[1]Cooper L, Meier A, Laporte MA, Elser JL, Mungall C, Sinn BT, Cavaliere D, Carbon S, Dunn NA, Smith B, et al. 2018. The Planteome database: an integrated resource for reference ontologies, plant genomics and phenomics. *Nucleic Acids Research* 46: D1168-D1180.

[2]Shrestha R. 2012. Bridging the phenotypic and genetic data useful for integrated breeding through a data annotation using the CropOntology developed by the crop communities of practice. *Frontiers in Physiology* 3.

[3]Cooper L, Walls RL, Elser J, Gandolfo MA, Stevenson DW, Smith B, Preece J, Athreya B, Mungall CJ, Rensing S. 2013. The plant ontology as a tool for comparative plant anatomy and genomic analyses. *Plant and Cell Physiology* 54: e1-e1.

[4]Ilic K, Kellogg EA, Jaiswal P, Zapata F, Stevens PF, Vincent LP, Avraham S, Reiser L, Pujar A, Sachs MM, et al. 2007. The plant structure ontology, a unified vocabulary of anatomy and morphology of a flowering plant. *Plant Physiology* 143: 587-599.

[5]Walls RL, Athreya B, Cooper L, Elser J, Gandolfo MA, Jaiswal P, Mungall CJ, Preece J, Rensing S, Smith B. 2012. Ontologies as integrative tools for plant science. *American Journal of Botany* 99: 1263-1275.

[6]Caracciolo C, Stellato A, Morshed A, Johannsen G, Rajbhandari S, Jaques Y, Keizer J. 2013. The AGROVOC linked dataset. *Semantic Web* 4: 341-348.

[7]Smith B, Ceusters W, Klagges B, Köhler J, Kumar A, Lomax J, Mungall C, Neuhaus F, Rector AL, Rosse C. 2005. Relations in biomedical ontologies. *Genome Biology* 6: R46.

[8]Alercia A, Diulgheroff S, Mackay M 2015. FAO/Bioversity Multi-Crop Passport Descriptors V. 2.1 [MCPD V. 2.1]-December 2015: Bioversity International.

[9]Yeumo ED, Alaux M, Arnaud E, Aubin S, Baumann U, Buche P, Cooper L, Ćwiek-Kupczyńska H, Davey RP, Fulss RA. 2017. Developing data interoperability using standards: A wheat community use case. *F1000Research* 6:1843.
